# Synthesis and anticancer potential of glycosides

**DOI:** 10.1039/d5ra07626a

**Published:** 2025-12-19

**Authors:** Shaukat Ali, Gul Rukh, Hanan A. Ogaly, Rahaf Ajaj, Abdur Rauf, Syed Adnan Ali Shah

**Affiliations:** a Organic Synthesis and Catalysis Research Laboratory, Institute of Chemical Sciences, University of Peshawar Peshawar 25120 Khyber Pakhtunkhwa Pakistan drshaukatali@uop.edu.pk; b Chemistry Department, College of Science, King Khalid University Abha 61421 Saudi Arabia; c Department of Environmental and Public Health, College of Health Sciences, Abu Dhabi University Abu Dhabi United Arab Emirates; d Department of Chemistry, University of Swabi Anbar 23561 Khyber Pakhtunkhwa Pakistan; e Faculty of Pharmacy, Universiti Teknologi MARA Cawangan Selangor Kampus Puncak Alam Bandar Puncak Alam 42300 Selangor D. E. Malaysia syedadnan@uitm.edu.my; f Atta-ur-Rahman Institute for Natural Product Discovery (AuRIns), Universiti Teknologi MARA Cawangan Selangor Kampus Puncak Alam Bandar Puncak Alam 42300 Selangor D. E. Malaysia

## Abstract

Cancer drug development faces significant challenges, primarily owing to the resistance of cancer cells. Recent efforts have focused on creating drugs with effective IC_50_ values and acceptable cytotoxicity standards as defined by organizations such as the IARC, UICC, AACR, WHO, and NCI. The synthesis of *S*-, *N*-, *C*-, and *O*-glycosidic linkages, protection of the glucose moiety, and modification tactics *via* alkylation, acylation, and esterification, α-glycosyl imidate formation, glycosyl radical functionalization, CuAAC reaction, and tagging and editing methods are also investigated. These efforts have streamlined the progress in the production of glycosidic compounds as antitumour agents because of their effectiveness and potency in MDR cancer cell lines. Despite their ultra-features, advanced cytotoxic analysis with robotics requires a deep understanding of their kinetics, which are studied by employing FID, TUNEL, ELISA, FRET, DNA cleavage, caspase, linear regression, and SRB assays. Meanwhile, the synthesised products were characterized using micro-FTIR, UFLC-DAD, cryogenic electron microscopy, elemental analysis, and molecular imaging techniques. Docking simulation, molecular dynamics simulations, and QSAR analyses helped in understanding the binding of the designed compounds to the active sites of the targeted cells. In this perspective, we explored the essential role of glycosidic compounds in synthesizing target-based drugs and their application in cancer treatment.

## Abstract 2

Glycosides as a tool against cancer cells resistance!

## Graphical abstract

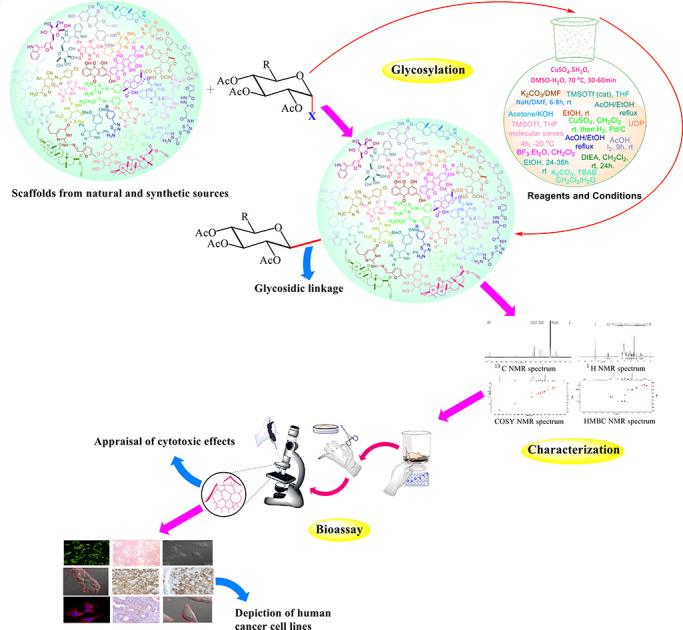

## 1. Introduction

The intersection of medicinal chemistry and various disciplines plays an important role in developing and modernizing anticancer drugs. Prehistoric drugs have been empirically discovered from plant-based natural products.^1^ Millenia have passed,^2^ but plant-derived medications are still vital in modern therapeutic systems.^3^ The recent century has witnessed the emergence of chemically diverse molecules in fighting against the outrageous spread of cancer. Statistics have projected cancer as the second leading cause of death after cardiovascular ailments.^4^ Current treatment methods often damage normal cells alongside cancer cells, driving ongoing development of anticancer agents that selectively target abnormally proliferating cells.^5^

Robert Bentley was the first person to detect an extract (obtained from *Podophyllum peltatum*) for its localized anticancer power in 1816,^6^ which was considered the first crude approximation toward advancement in this field. Gradually, this revolutionary idea encompassed new concepts and adopted the name chemotherapy in the late 1940s and the 1950s. This breakthrough led to the development of a new class of chemotherapeutic drugs, which includes several selective medicines such as adriamycin (1970), cytoxan (1958), 5-FU (1957), cisplatin (1971), and vinblastine (1960).^7^ In 1955, researchers conducted a clinical trial authorized by the National Cancer Institute, inspired by this groundbreaking idea. This era is now known as the ‘golden age’ of chemotherapy.^8^ Later, around 2011, the World Health Organization estimated that 12 million fatalities would occur by 2030.^9^ Subsequently, a prediction was made that 27.5 million deaths would occur by 2040.^10^

To address this dilemma, small molecules^11^ demonstrating extreme chemotherapeutic behaviour were discovered and identified. In the 1990s, GLUT-facilitated treatment gained widespread attention because glycoconjugates exhibited enhanced solubility and reduced local toxicity.^12^ The US FDA and the National Medical Products Administration (NMPA) have approved 89 small molecules for use as anticancer drugs, one of which is a glycoside.^13^ Glycosides can be used in natural form or in conjugation with other promising compounds,^14^ owing to the Warburg effect.^15^

The latest derivatives of carbohydrates have been designed using the sugar-tail methodology,^16^ in which the glycosidic linkage is the core of carbohydrate chemistry. The biological activity of glycosides can be improved by changing the stereochemistry of their sugar units.^17^ Here, we present some of the crucial steps for glycosylation. (1) Formation of alkyl and aryl glycosides (at non-classical sites^18^), (2) etherification with alcohol or phenol in the presence of silver, mercury or cadmium (Koenigs–Knorr synthesis) or with alkyl metal salt (Michael synthesis),^19^ (3) activation of glucose to generate α-glycosyl imidate,^20^ (4) ring-closing metathesis (RCM),^21^ (5) atom-economic step towards the generation of 2-deoxy sugars, and (6) installation of directing groups to control α/β anomeric mixtures (on classical sites^18^) selectivity. (7) Removal of protecting or directing groups *via O*-alkylation or acylation facilitates the production of stereoselective products.^22^ (8) Stereoselective glycosyl radical functionalization using a Ti catalyst.^23^

Eventually, all these approaches produce glycosidic linkages, including *S*- (oppose hydrolysis),^24^*N*- (frequently hydrolysed), *C*- (can withstand metabolic hydrolysis), and *O* (susceptible to hydrolysis)-glycosidic bonds.^17,25^ In short, promoieties are attached to hinder the chemoenzymatic transformation of the drug or to adjust its pharmacokinetic profile.^26^

The bioavailability of carbohydrate-derived drugs is often increased by protecting their polar hydroxyl groups such as hydrophobic acyl group.^27^ After reaching the target organs, these esters undergo enzymatic hydrolysis^28^ to release the active compounds. Furthermore, several attempts have been made to improve the water solubility and reduce the toxicity of pro-drugs *via* the formation of glycoconjugates.^29^

All the above-mentioned factors are important considerations, but they have some limitations, such as the susceptibility of *O*-glycosides to enzymatic degradation. Consequently, the insertion of a methylene group to substitute the glycosidic oxygen can improve its effectiveness. Notably, *C*-glycosides are resistant to enzymatic action,^30^ and thus alternatives to natural *O*-glycosides.^25^ Likewise, the orientation, location, and number of hydroxyl substituents can affect the breakage of glycosidic bonds.^31^ Besides these facts, nanoencapsulation systems, such as polymeric micelles,^32^ gold nanoparticles,^33^ mesoporous silica,^34^ magnetic carbon-based nanoparticles,^35^ cyclodextrins,^32^ hydrogels, microneedles,^36^ and cost-effective as well as harmless liposomes (as nanocarriers or lipid nanoparticles)^37^ are fortunately satisfactory for the beneficial transportation of specific products. This underlines the need for improved oncology research to successfully revive the previously collapsed area of medical chemistry and overcome the challenges of the massive global cancer burden.

Glycosides exhibit anticancer properties through various mechanisms, often involving specific interactions with cellular targets. For example, cardiac glycosides such as digoxin and ouabain inhibit Na^+^/K^+^-ATPase by binding to its α-subunit (*K*_d_ ∼ 1–10 nM, forming hydrogen bonds with Asp121 and Asn122), which disrupts the ion balance and triggers apoptosis through calcium-dependent signalling pathways.^38^ Similarly, gallic acid-based glycoconjugates, attach to the colchicine site on tubulin (*K*_a_ ∼ 10^5^ M^−1^, forming hydrogen bonds with Lys352), preventing polymerization and leading to G2/M phase arrest in breast cancer cells.^39^ This review explores the binding properties of glycosides using advanced techniques, such as docking, molecular dynamics (MD), and cryogenic electron microscopy (Cryo-EM), to analyse their interactions with targets, such as topoisomerase II, EGFR, and lysosomal proteins.

Acetylated glycosides function as prodrugs to enhance the lipophilicity, thereby facilitating cellular uptake. Their acetyl groups assist in membrane translocation, with subsequent deacetylation producing active forms that bind cytosolic targets.^41^

## 2. Synthesis of aloe-emodin glycosides

**Scheme 1:**
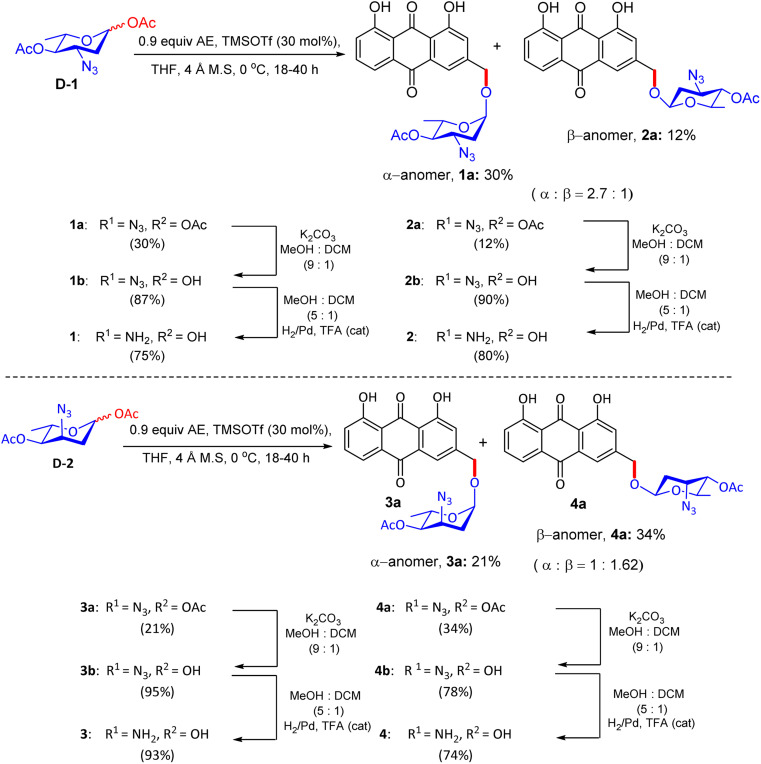
Schematic of the preparation of AEGs 1–4.

One of the appealing methods for the synthesis of aloe-emodin glycosides (AEGs) stems from the procedure adopted by Fridman *et al.* in 2011.^41^ They synthesized two 3-azido-4-*O*-acetyl-protected glycosyl acetate donors (acosamine and ristosamine derivatives) using literature procedures.^40^ These derivatives vary in absolute configuration at C-3 azides from the accessible 3,4-di-*O*-acetyl-l-rhamnal in three consecutive steps (Scheme 1). Glycosylacetates were first reacted with AE in THF at 0 °C for 18–40 h to afford α/β anomeric mixtures of AEGs 1a–4a in the presence of Lewis acid-catalyzed activation and were easily separated by reverse-phase HPLC. In the next phase, AEG compounds 1b–4b were obtained by deacetylation under basic conditions. Extraction was carried out as size exclusion column chromatography on Sephadex LH-20. The azido groups were eventually converted to their corresponding free amines through catalytic hydrogenation with Pd/C and trifluoroacetic acid in the mixed solvent of methanol and dichloromethane. Finally, reverse-phase HPLC dispensed the four products of AEGs 1–4 in relatively high yields. The variations in these compounds are based on the configuration of the glycosidic linkages and position of the amine moiety on the carbohydrate C-3. The library of compounds was characterized by ^1^H, ^13^C NMR spectroscopy and HRMS.

### 2.1. Findings through supercoiled plasmid DNA unwinding gel assay

Fridman reported^41^ that the amino sugar moiety attached to the anthraquinone core significantly improved the cytotoxic activity of aloe-emodin (AE), which was examined in the MOLT-4, OVCAR, SKOV-3, and NAR cancer cell lines, as shown by the data given in Table 1. Among the newly synthesized AEG compounds, AEG 1 (synthesized by combining an α-glycosidic bond with an equatorial C-3 amine). AEG 1 was twice as active against many DOX-resistant cells as AE and anthracycline doxorubicin (DOX). The supercoiled plasmid DNA unwinding gel assay confirmed that AEG did not intercalate DNA but instead used a different mechanism of action. The enhanced cytotoxic effect of AEG was confirmed by cell viability test results, which showed only a decrease in cell proliferation by DOX, whereas the death of cells was less than one cell cycle by AEGs. Fridman *et al.* also studied the cytotoxicity of AEG 1 in normal human lymphocytes. It was shown that AEG 1, with an IC_50_ value of approximately 3 µM, was significantly cytotoxic to human lymphocytes even after a short 3 h incubation period, compared to DOX and AE. The hemolytic effect of these compounds on RBC was also observed, and it was found that AEGs do not cause cell lysis as detergents; therefore, rapid cell death is not caused by lysis. Confocal microscopy was used to confirm the improved potency of the synthetic AEGs. The results of confocal microscopy proved that AEG 1 was able to penetrate DOX-resistant cancer cells, as a significant amount of this compound was detected inside the cells, while DOX accumulated in the cell membrane. Fridman and colleagues concluded that anthracycline-resistant tumors caused by P-gp efflux pumps can be overcome by cytotoxic agents such as AEGs. Binding studies have demonstrated that the α-glycosidic bond and equatorial C-3 amine of AEG 1 facilitate enhanced cytosolic penetration, effectively circumventing P-gp efflux in DOX-resistant cells (NAR and OVCAR). Docking simulations indicated that AEG 1 engages with cytosolic targets, potentially including mitochondrial proteins or signalling kinases, through the formation of hydrogen bonds with polar residues such as Ser/Thr residues. Confocal microscopy confirmed the cytosolic localization of AEG 1, which was distinct from the membrane accumulation observed with DOX, thereby supporting a non-DNA-intercalating mechanism that induces apoptosis, possibly *via* mitochondrial pathways. Micro-FTIR analysis verified the presence of the glycosidic bond, while molecular imaging elucidated the subcellular distribution of AEG 1.

**Table 1:** Cytotoxicity of AEGs 1–4 in different cancer cell lines

Compound no.	IC_50_ (µM)			
	MOLT-4	OVCAR	SKOV-3	NAR
DOX	0.2	>20	>20	>100
AE	>20	>20	>20	>100
AEG 1	5.8	5.2	6.9	8.6
AEG 2	7.6	6.4	>20	>100
AEG 3	5.4	15.7	13.5	18
AEG 4	12.8	>20	>20	28.3

## 3. Synthesis of solasodine glycosides

**Scheme 2:**
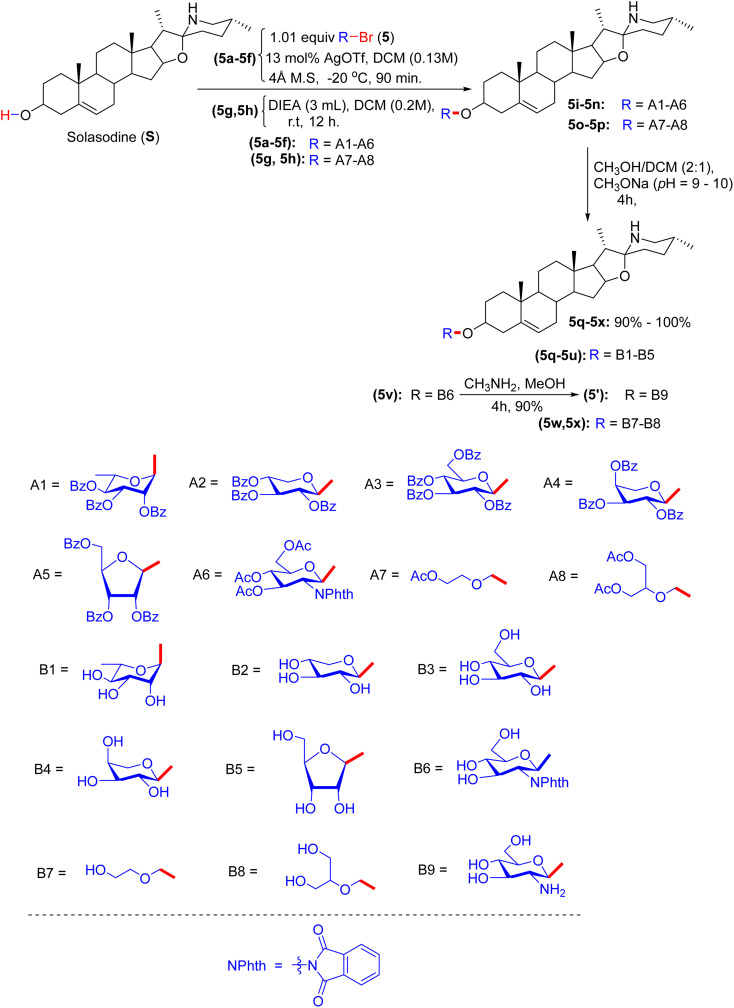
Synthesis of solasodine glycoside derivatives.

In early 2012, the Lou group realized progress by synthesizing solasodine glycosides. According to their report, the process started with the preparation of solasodine (S) using a known procedure over five consecutive reactions.^3^ However, the actual pathway was systematically initiated by treatment of solasodine with glycosyl bromides 5a–5f, which produced the first series of solasodine glycosides *via* the Koenigs–Knorr glycosylation method (Scheme 2). Compounds 5g–5h were derived by attaching two open-loop saccharide analogues to solasodine in the presence of *N*,*N*-diisopropylethylamine (DIEA). Donors 5a–5f react with solasodine in silver trifluoromethanesulfonate (AgOTf), acting as a catalyst and a protecting agent, resulting in protected compounds 5i–5n. Similarly, open-loop donors 5g or 5h reacted with solasodine (S) in the presence of DIEA to obtain intermediates 5o and 5p. Consequently, the hydrolysis of tosyl, acetyl, and phthalic anhydride in MeOH/MeONa or CH_3_NH_2_ yielded the desired compounds 5q–5x in good yields. The final compounds were analyzed by IR and ^1^H-NMR spectroscopy.

### 3.1. Findings through MTT assay, SAR and binding characteristics of compounds

Solasodine glycosides (saponins 5q–5u, 5w, 5x, and 5′, and solasodine, S) were evaluated for cytotoxicity in the PC3, K562, MCF-7, ECV304, and HL7702 cell lines by MTT assay, showing marked anticancer activity in PC3 and K562 cells (IC_50_ ∼ 1–3 µM), limited activity in the MCF-7 cell line (∼5–10 µM), and negligible effects in the normal cell lines ECV304 and HL7702 (IC_50_ > 40 µM) (Table 2).^3^ Docking studies revealed that 5q, 5w, and 5′ bind PI3K (PDB: 3L08) or caspase-3 (PDB: 1GFW, *K*_d_ ∼1–3 µM), with acetylated β-d-glucose or rhamnose-forming H-bonds (for example, Asp933 and Arg207) and the solasodine core enabled hydrophobic interactions with Trp812 or Phe256. Triazole-linked glycosides (*e.g.*5′) enhanced the H-bonding with Asp855. Compounds 5r–5u and 5x and solasodine (S) show weaker binding (*K*_d_ ∼3–5 µM) due to their deprotected glycosides or lack of sugars. SAR analyses highlighted the lipophilicity and rigidity of acetylated glycosides, with rhamnose or aryl substituents boosting the potency in PC3/K562 cells. Micro-FTIR confirmed the presence of glycosidic (C–O ∼ 1000–1100 cm^−1^), acetyl (C=O ∼ 1700 cm^−1^), and solasodine (C–N ∼ 1200 cm^−1^) bonds, UFLC-DAD verified their purity, and molecular imaging showed their cytosolic localization. MoA is involved in PI3K inhibition, reduced proliferation, or caspase-3 activation, promoting apoptosis, with high potency in PC3/K562 cells due to target overexpression and selectivity from cancer-specific uptake. The binding characteristics of 5q, 5w, and 5′ position them as promising leads, complementing the Na^+^/K^+^-ATPase and gallic acid glycoconjugate tubulin targeting by cardiac glycosides.^39^

**Table 2:** Cytotoxicity of potent compounds S, 5q–5u, 5w, 5x, and 5′ against different human cancer cell lines

Compound no.	IC_50_ (µM)					
	KB	K562	MCF-7	PC3	ECV304	HL7702
S	ND	ND	ND	13.6	ND	ND
5q	29.1	18.8	14.2	18.4	34	36
5r	ND	51	53	16.5	ND	ND
5s	ND	43	77	27.9	ND	ND
5t	ND	44	64	23.1	ND	ND
9u	ND	ND	ND	66.9	ND	ND
5′	ND	37	98	21.9	ND	ND
5w	28.9	112	12.9	17.4	>40	>40
5x	29.4	17	26	7.2	>40	>40
Solamargine	7.8	8	8.2	5.9	ND	ND

## 4. Synthesis of saponin glycosides

Hongxiang Luo's group^3^ anticipated that saponins with rhamnose (compound 5p) or open-loop saccharide analogs (compounds 5w and 5x) would exhibit much higher anticancer activity than synthesized derivatives and accelerate cell apoptosis at an extraordinarily high speed. One of the observations was the genesis of crescent-shaped figures or globular structures (apoptotic bodies) located near the periphery of the nucleus, which were also observed at a certain point in the analysis.

**Fig. 1:**
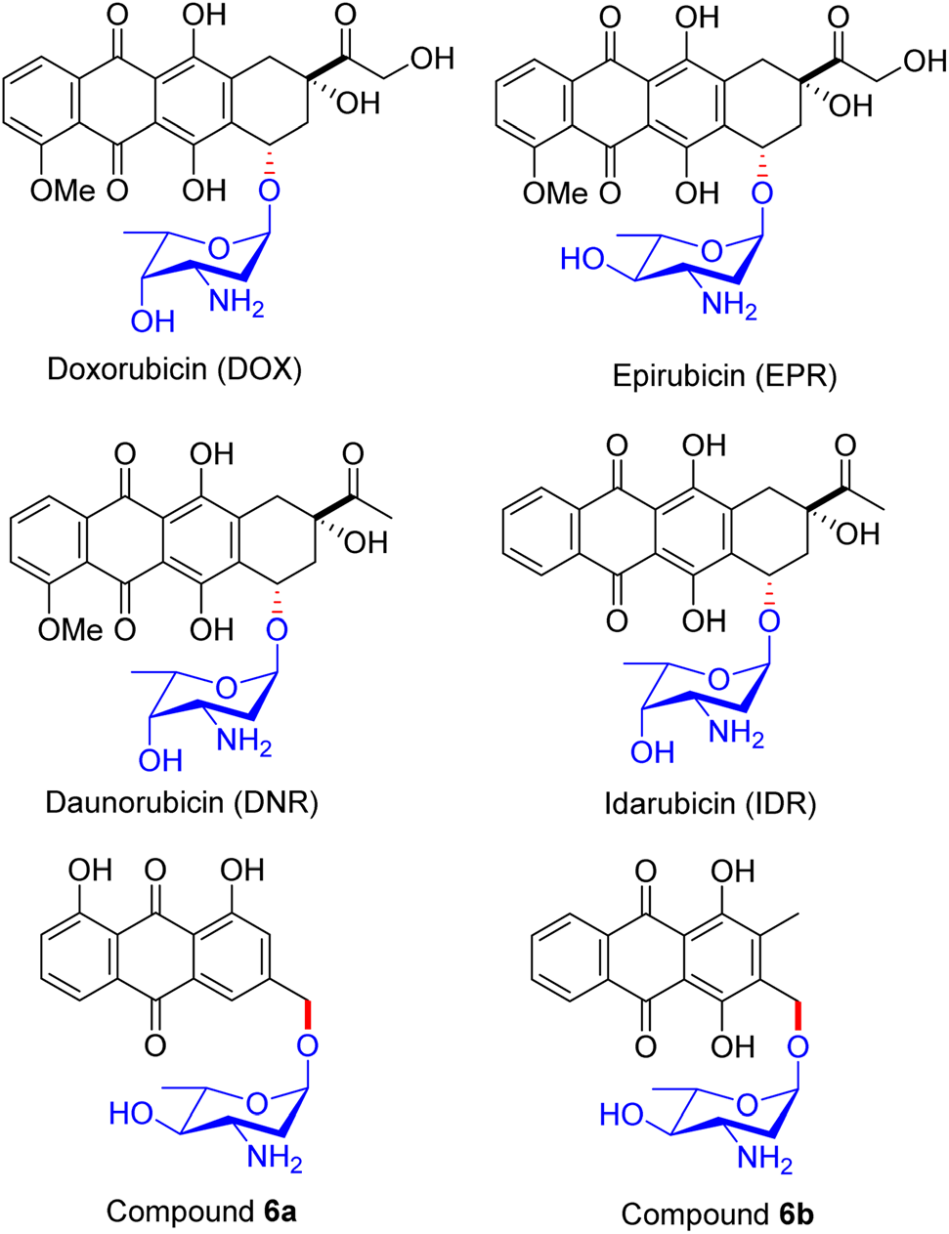
Structures of glycosylated anthracycline derivatives.

**Scheme 3:**
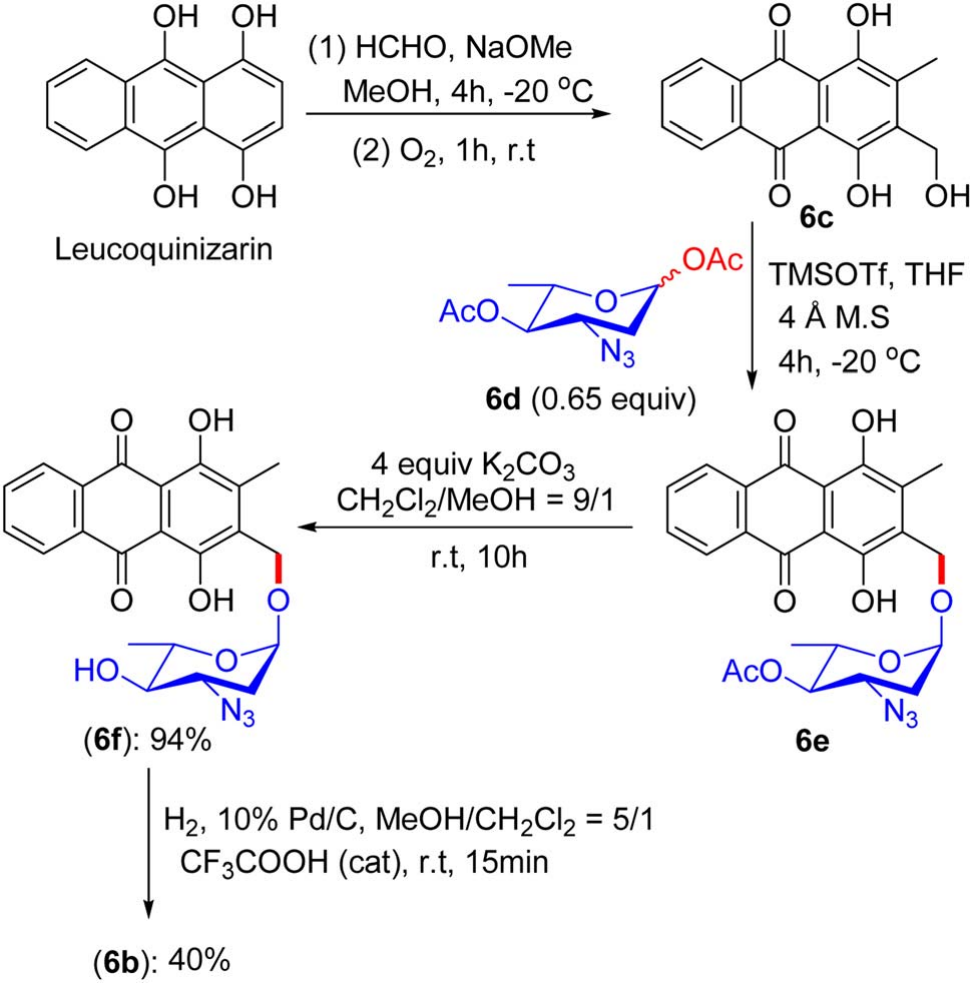
Synthetic route to 3-methyl-digiferrol glycoside 6b.

An alternative fascinating process emerged from Fridman's report in 2013. They devised a proper synthetic scheme by exploiting the existing approach to generate the aloe-emodin glycoside 6a (Fig. 1).^42^ The synthesis of anthraquinone 3-methyldigiferrol 6c was achieved from leucoquinizarin *via* the Marschalk reaction (Scheme 3). Subsequently, compound 6c was treated with acosamine glycosyl acetate derivative 6d with the assistance of trimethylsilyl trifluoromethanesulfonate in tetrahydrofuran at −20 °C to furnish the required glycosylated product. The protected α-glycoside 6e was then purified by C-18 reverse-phase HPLC. Consequently, the deacetylation of 6e in the presence of K_2_CO_3_ in CH_2_Cl_2_/MeOH solution afforded compound 6f, which was eluted by size-exclusion chromatography on Sephadex LH-20. The azido group of 6f was converted to a free amine by catalytic hydrogenation in MeOH/CH_2_Cl_2_ solution at room temperature. Finally, 3-methyldigiferrol glycoside 6b was separated by passing through C-18 reverse-phase HPLC.

### 4.1. Findings through XTT assay, SAR, and binding characteristics of compounds

The synthesized compounds were tested for their cytotoxicity in the SKOV-3, MCF-7, DA3, and ES2 cell lines (the data are summarized in Table 3). Predominantly, DOX is effective against DA3 cells, although compounds 6a and IDR act on almost all the cell lines. Given that compound 6a and IDR attack or evade different modes of action, to combat drug resistance, other cell lines displayed resistance to DOX. The correlation between DOX and compound 6a and IDR has shown impulsive cytotoxic effects in DA3 cells that interact independently of the cell cycle. The location of derivatives inside the cell was ascertained by a confocal microscopic study. Modifications in the central core of the derivatives can intensify the activity and locality inside the cell. IDR, DNR, and other derivatives 6a and 6b (Fig. 1) have also shown colocalization in the lysosome and exclusive DOX agglomerates in the nucleus. Lysosomes incorporated the anthracycline derivatives, which clearly explains their trajectory of invasion. Similarly, subcellular distribution, cytotoxic activity, and mode of action can be affected by minor structural differences in the anthracycline skeleton. The anticancer efficacy of saponin glycoside 6a is attributed to its ability to disrupt lysosomal membranes, as demonstrated by cryo-electron microscopy and confocal microscopy. Docking studies indicate that the glycosidic moiety of 6a forms hydrogen bonds with lysosomal membrane proteins, such as LAMP1, leading to membrane destabilization and the subsequent release of cathepsins, which initiate apoptosis. The structural integrity of 6a was confirmed through UFLC-DAD, while molecular dynamics simulations corroborated its binding stability, underscoring the significance of the glycoside in enhancing lysosomal targeting. These analyses explained that potent novel antitumor drugs (which will not interfere with the normal cell division) could be introduced in the near future based on a remodelled anthraquinone framework or utilizing the three-ring anthraquinone-based anthracyclines.

**Table 3:** Cytotoxicity of anthracycline derivatives

Compoun no.	IC_50_ (µM)			
	SKOV-3	MCF-7	DA3	ES2
6a	11.1	23.6	7.9	6.8
6b	16.3	17.2	17.5	17.7
DOX	55.7	>60	4	>60
IDR	206	33.9	1.9	25.5
DNR	4.5	7.3	2.3	3.2

## 5. Synthesis of 2-thioxoimidazolidin-4-one and benzothiazole thioglycosides

**Scheme 4:**
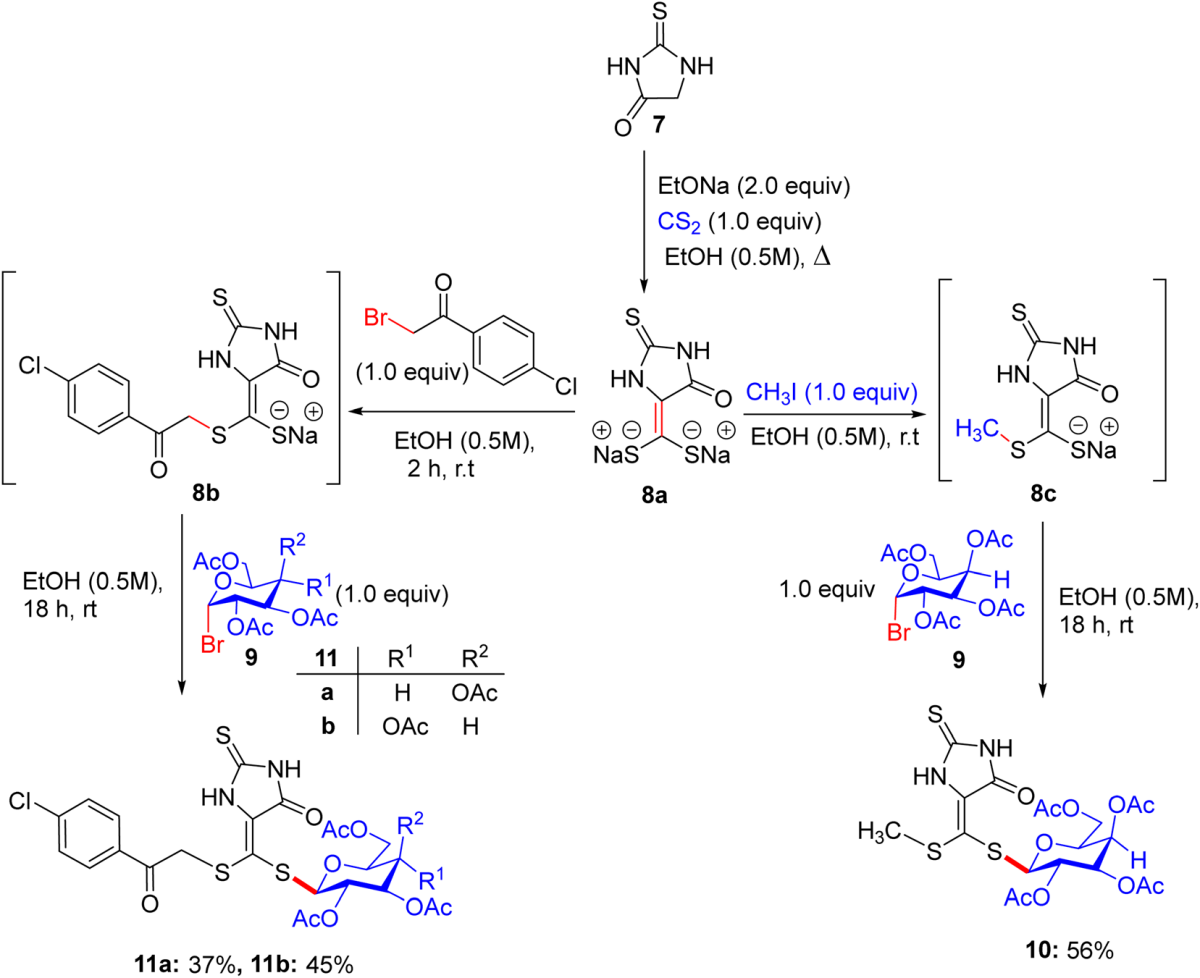
Synthesis of 2-thioxoimidazolidin-4-one thioglycoside derivatives 10 and 11.

In early 2014, Elgemeie GH proposed a new idea for modification in this vast field of research and synthesized α-glycosyl halides by reacting heterocyclic thioglycosides with thiolate salts, in which heterocyclic glycosides were employed for the construction of new carbohydrate derivatives (Scheme 4).^43^ By starting with 2-thioxoimidazolidine-4-one 7, compounds 2-thioxoimidazolidin-4-one thioglycosides 10, 11a, and 11b were obtained. As a pioneering step, compound 7 reacted with carbon disulfide in the presence of sodium ethoxide and formed sodium dithiolate salts 8a. In the following reaction, sodium-2-thioxoimidazolidin-4-one-5-methylenedithiolate 8a was monoalkylated, resulting in the production of stable sodium salts of monoalkylthio derivatives. Hence, an equivalent of methyl iodide or 4-chlorophenacyl bromide supplies the appropriate amount of sodium-2-thioxoimidazolidin-4-one-5-(methylthio)-[2-oxo-2-(4-chlorophenylethyl)thio]-methylenethiolate salts 8b and 8c in crude form. Compounds 8b and 8c were treated with tetra-*O*-acetylated *gluco*-/*galacto*-pyranosyl bromides 9 at ambient temperature in ethanol for the manipulation of *S*-glucoside 11a, *S*-galactoside 11b, and *S*-glucoside 10 in good yields.

**Scheme 5:**
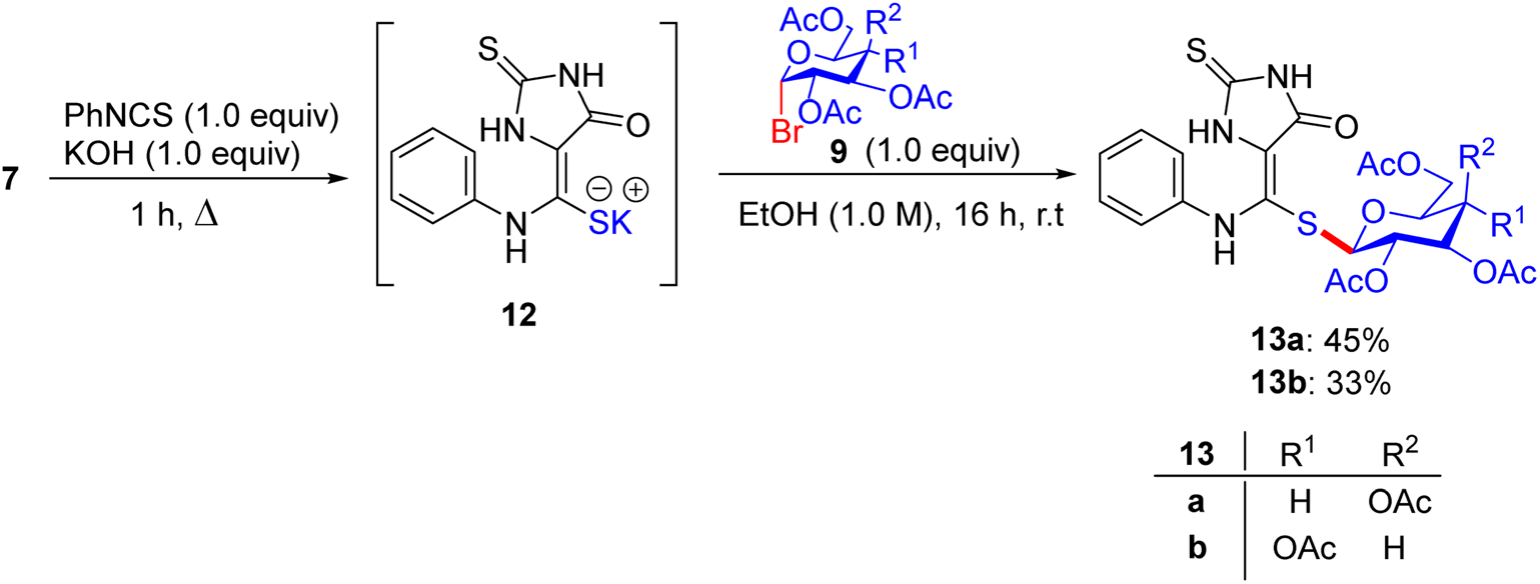
Synthetic route to 2-thioxoimidazolidin-4-one thioglycoside derivatives 13a and 13b.

The starting hydantoin 7, together with phenylisothiocyanate in ethanolic potassium hydroxide, provided an amazing intermediate, potassium-2-thioxoimidazolidin-4-one-5-(phenylamino)-methylenethiolate salt 12, on heating (Scheme 5). Unexpectedly, the reaction of crude 12 with the blocked *gluco*- and *galacto*-pyranosyl bromides 9 in ethanol at room temperature afforded *S*-glucoside 13a and *S*-galactoside 13b in 45% and 33% yields, respectively.

**Scheme 6:**
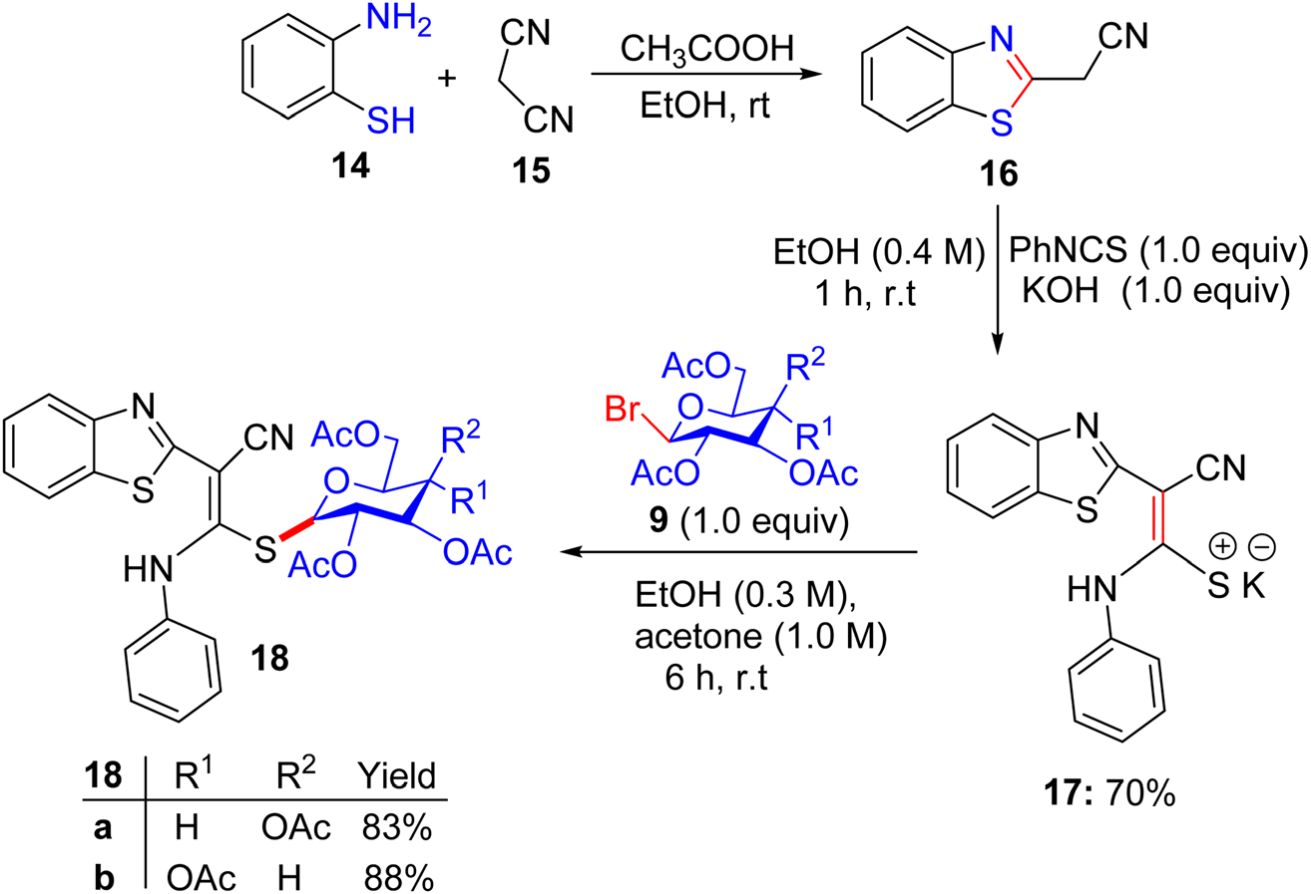
Synthetic pathway for the synthesis of benzothiazole thioglycosides 18a and 18b.

For a good reason, potassium 2-[(2*E*)-2-(phenylamino)-1-(cyano)vinyl]1,3-benzothiazole-2-thiolate salt 17 was chosen as a vital intermediate for the production of a new series of glycosides, *i.e.* benzothiazole thioglycoside derivatives (Scheme 6). First, 2-amino thiophenol 14 was reacted with malononitrile 15 in acetic acid in absolute ethanol to obtain benzothiazole acetonitrile 16. Subsequently, benzothiazole acetonitrile 16 was treated with phenylisothiocyanate in ethanolic KOH to afford the equivalent stable potassium 2-[(2*E*)-2-(phenylamino)-1-(cyano)vinyl] 1,3-benzothiazole-2-thiolate salt 17. Ultimately, compound 17 was reacted with 2,3,4,6-tetra-*O*-acetyl-α-d-*gluco*- and *galacto*-pyranosyl bromides 9a and 9b in ethanol at ambient temperature to give the corresponding *S*-glucoside 18a or *S*-galactoside 18b, respectively.

### 5.1. Findings through MTT assay and binding characteristics

Five synthesized compounds were examined for their antiproliferation effect on MCF-7 cells (Table 4). Elgemeie *et al.*^43^ proposed that each compound exhibited cytotoxicity, with IC_50_ values ranging from modest to high at 3.99–41.00 µM. Compound 11a was the most active compound in terms of functionality, followed by 13a and 10. Conversely, the *S*-glucoside series was more active than compounds 11b and 13b. Furthermore, the assay suggested that benzothiazole *S*-glucosides 18b are more active than 18a. Compound 11a, an *S*-glucoside with a 2-thioxoimidazolidin-4-one moiety, demonstrated higher potency against MCF-7 cells (IC_50_ 3.998 µM) than novobiocin. Docking studies have suggested that it binds to topoisomerase II or Hsp90, forming hydrogen bonds with polar residues. The thioglycosidic linkage enhances binding affinity owing to the electron-donating properties of sulfur. Compounds 13a and 10 showed moderate activity, whereas 11b and 13b (*S*-galactosides) were less active owing to weaker hydrogen bonding. Benzothiazole *S*-glucoside 18b exhibited high activity, likely by binding to Hsp90 or EGFR with enhanced π–π stacking. Various techniques, including micro-FTIR, UFLC-DAD, and docking simulations, have been used to characterise these compounds and elucidate their binding mechanisms. The primary mode of action of these compounds involves inhibition of topoisomerase II or Hsp90, leading to apoptosis in cancer cells.

**Table 4:** Cytotoxicity of heterocyclic thioglycosides

Compound no.	IC_50_ (µM)
	MCF
10	41
11a	3.998
11b	4.291
13a	6.215
13b	14.673
18a	4.212
18b	23.623
Novobiocin	418.313

## 6. Synthesis of triazolyl 18β-glycyrrhetinate glycosides

**Scheme 7:**
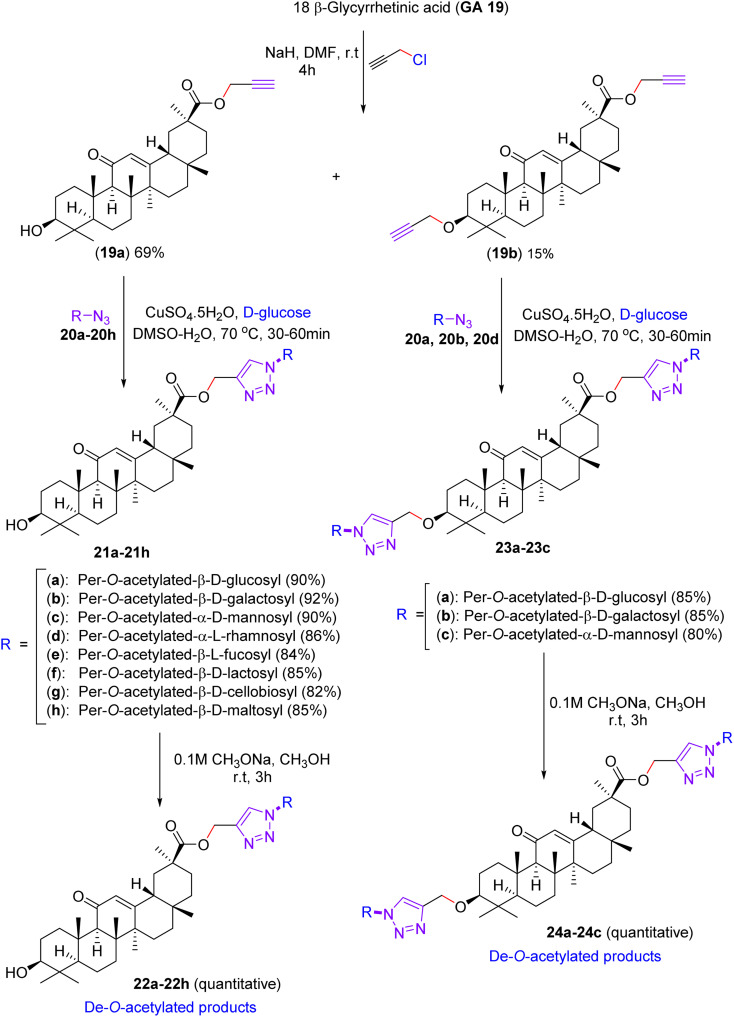
Synthesis of triazole-linked glycosylated glycyrrhetinic acids (22a–22h) and (24a–24c).

Later, in June 2014, in terms of advancement, an alternative synthesis was disclosed by Jana, Biswas, and Misra for the development of a library of glycosylated triazolyl 18β-glycerrhetinic acid derivatives.^44^ This process was accomplished by condensing glycosyl azide derivatives with propargyl ester of 18β-glycerrhetinic acid *via* a copper-catalyzed click reaction approach, in which the 18β-glycyrrhetinic acid compound (GA 19) was treated with propargyl chloride in the presence of sodium hydride in DMF to afford the propargyl ester of 18β-glycyrrhetinic acid 19a in 69% yield and dipropargylated 18β-glycyrrhetinate derivative 19b in 15% yield (Scheme 7). Then 19a was reacted with glycosyl azides (20a–20h) in the presence of aq. CuSO_4_·5H_2_O and aq. d-glucose in DMSO–H_2_O (1 : 2) at 70 °C for 30 min to give glycosyl 1,2,3-triazolyl GA derivatives (21a–21h) in excellent yields. Similarly, dipropargylated GA derivative 19b was reacted with 20a, 20b, and 20d, affording bis-triazolyl derivatives 23a–23c in good yields. The de-*O*-acetylation of compounds 21a–21h and 23a–23c with the help of sodium methoxide in methanol at ambient temperature ultimately resulted in the synthesis of compounds 22a–22h and 24a–24c, respectively, having free hydroxyl groups on their sugar moieties, in quantitative yields.

### 6.1. Findings through ELISA, TUNEL assays, and binding characteristics of compounds

The anti-cancer properties of 18β-glycyrrhetinic acid (GA 19), glycyrrhizin (GN), and others were appraised by testing them on the HeLa and NKE cell lines. In contrast, resveratrol was used as a reference drug (a summary of the tests is presented in Table 5). The results suggest that compound GA 19 and compound 22d were more effective in the case of HeLa cells but did not exhibit any appreciable effects on the NKE cancer cell line. Compound 22g and GN exhibited almost similar IC_50_ values in both cases, and compound 24c showed moderate IC_50_ values for both cell lines.

**Table 5:** Cytotoxicity of the glycyrrhetinic acid derivatives

Compound no.	IC_50_ (µM)	
	HeLa	NKE
GA 19	12.22	47.38
GN	37.77	29.32
22a	>40	>40
22b	>40	>40
22c	>40	31
22d	13.76	61
22e	>40	27.89
22f	>40	>40
22g	22.49	22.41
22h	>40	>40
24a	>40	34.77
24b	>40	>40
24c	21.5	50.56
Resveratrol	20.35	46.57

Compounds GA 19 and 22d and resveratrol were further evaluated for their ability to induce apoptosis. Compound 23d exhibited higher apoptotic activity compared to GA 19, as substantiated by ELISA and TUNEL assays. This study was focused on finding the role of mitochondria in apoptosis in cancerous and normal cells. After numerous considerations, it was validated that the symmetry of compound 23d was such that it could cause observable mitochondrial damage in HeLa cells (cancer) and GA 19-affected NKE (normal) cells. Considering this evidence, we suggest that the synthesized compounds are potent anti-cancer agents plainly against cervical cancer, and advanced studies are underway. Triazolyl glycosides (21a–23c) deacetylate to enhance mitochondrial or kinase binding.^44^ The compounds in Table 5 include triazolyl 18β-glycyrrhetinic acid glycosides (22d, 22g, 23d, and 24c), glycyrrhizin (GN), and GA 19. Compound 22d showed potent activity against HeLa cells (IC_50_ = 13.76 µM) with lower toxicity to NKE cells (IC_50_ = 61 µM). Docking studies suggest that 22d binds to mitochondrial proteins, such as Bax and VDAC, with triazole and glycoside forming hydrogen bonds. GA 19 exhibited similar potency in HeLa cells but lower selectivity. Compound 23d demonstrated the highest apoptotic activity, likely owing to enhanced mitochondrial targeting. GN and 22g showed moderately equivalent activities in both cell lines. Compound 24c showed moderate activity, whereas GA 19 (aglycone) was less effective than the glycosides. Triazolyl glycosides generally induce mitochondrial apoptosis, with their binding affinities and cellular effects correlating with their structural features. Analytical techniques, such as micro-FTIR, UFLC-DAD, and molecular imaging, were used to characterise the compounds and their interactions.

## 7. Synthesis of podophyllotoxin glycosides

**Scheme 8:**
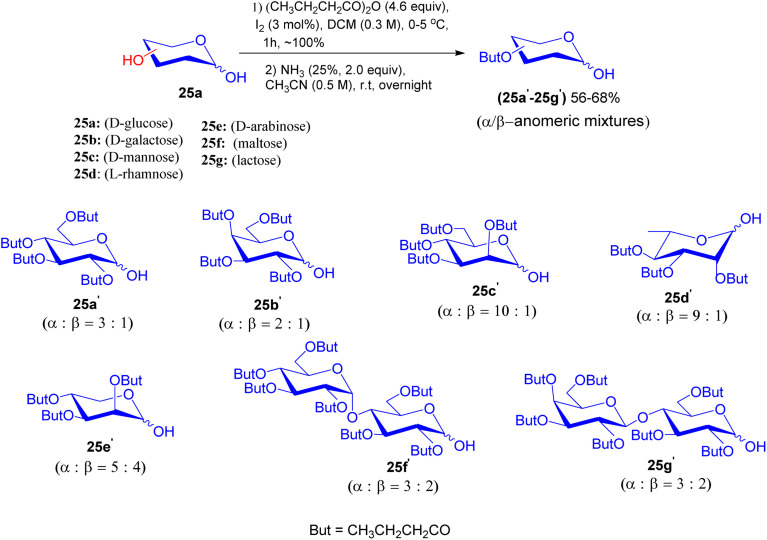
Synthesis of per-butyrylated sugar units 25a′–25g′.

In 2015, Jiang-Miao Hu *et al.* disclosed an experimental procedure that gives the glycosidic linkages of podophyllotoxin glycosides using glycosyl iodide and silyl glycoside donors.^45^*O*-Butyryl protected sugar units 25a′–25g′ were prepared from distinct sugar units 25a–25g with butyric anhydride promoted by iodine and were further subjected to 25% ammonia solution in acetonitrile to generate 25a′–25g′ as anomeric mixtures in 56–68% yield, respectively (Scheme 8).

**Scheme 9:**
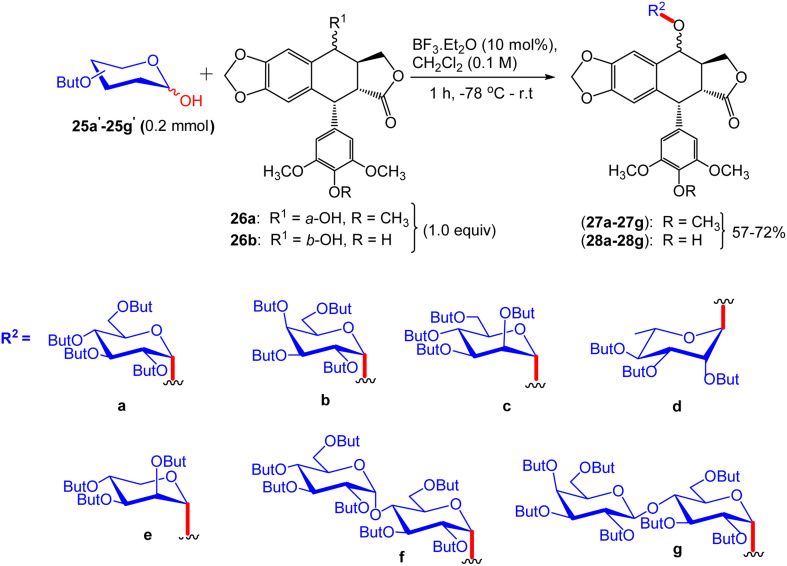
Glycosylation of podophyllotoxin and its derivatives 26a and 26b with per-butyrylated sugar units.

The second step involves glycosylation of podophyllotoxin (26a) with 25a′–25g′ in the presence of trifluoroboron etherate (BF_3_·Et_2_O) to give glycosylated epipodophyllotoxin 27a–27g, respectively (Scheme 9). Under similar conditions, compound 4′-demethylepipodophyllotoxin (26b) with 25a′–25g′ afforded α-glycosidic compounds 28a–28g, respectively (Scheme 9).

**Scheme 10:**
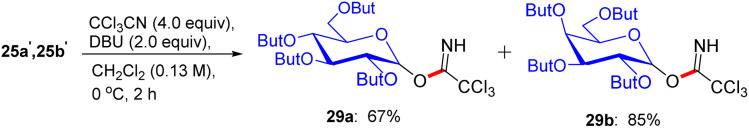
Protection of *O*-butyryl-protected sugar units 25a′ and 25b′ with imidates.

**Scheme 11:**
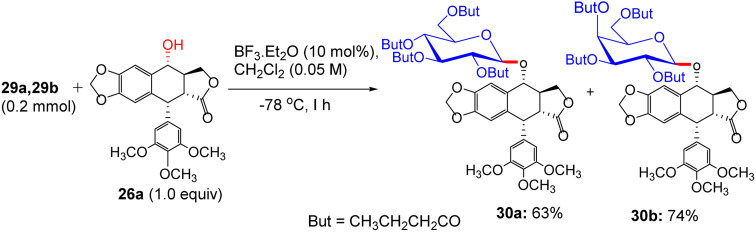
Glycosylation of podophyllotoxin 29a and 29b with glycosylated imidates.

The synthesis of trichloroacetimidates 29a and 29b was accomplished from their corresponding 1-OH compounds 25a′ and 25b′ with trichloroacetonitrile (CCl_3_CN) and 1,8-diazabicyclo[5.4.0]undec-7-ene (DBU) as a catalyst (Scheme 10). Glycosylation of 26a in the presence of a catalytic amount of BF_3_·Et_2_O at −78 °C to ambient temperature produced podophyllotoxin β-glycosides 30a and 30b in 60% and 68% yields, respectively (Scheme 11). Finally, the desired products were characterized using the ^1^H NMR, ^13^C NMR, ESI-MS, and HRESI-MS techniques.

### 7.1. Findings through MTT assay and binding characteristics of compounds

Recognizing their significant anticancer properties, epipodophyllotoxin glycosides were evaluated across five human cancer cell lines (HL-60, SMMC-7721, A-549, MCF-7, and SW480), with the findings detailed in Table 6. d-Galactopyranoside (27b) and d-arabinopyranoside (27e) demonstrated notable effectiveness (IC_50_ = 0.14–1.65 µM), with docking analyses showing their robust interaction with the DNA-binding cleft of topoisomerase II (*K*_d_ ∼0.1–1.5 µM), forming hydrogen bonds with Arg503, Gln778, and Asp479.^45^ Similarly, β-d-glucopyranoside (30a) with podophyllotoxin as the aglycone showed strong activity (IC_50_ = 0.61–1.69 µM), with glucose hydroxyl groups enhancing its hydrogen bonding with Thr821 (*K*_d_ ∼ 0.3–1 µM). Conversely, other glycosides, such as those based on mannose, exhibited reduced activity (IC_50_ > 5 µM) due to their weaker binding (*K*_d_ ∼ 5–10 µM), highlighting the importance of sugar residues. 4′-Demethylepipodophyllotoxin glycosides showed similar activity, with docking studies indicating their improved hydrogen bonding (*e.g.*, with Lys720) due to their increased hydrophilicity. Micro-FTIR confirmed the presence of glycosidic bonds (C–O stretch ∼ 1000–1100 cm^−1^), UFLC-DAD confirmed their purity, and QSAR linked the β-linkage and C-4 α-configuration with their potency. Molecular imaging suggests their nuclear localization, supporting the inhibition of topoisomerase II, which stabilizes DNA strand breaks and triggers apoptosis. These binding characteristics underscore the crucial impact of the glycosidic linkage (α *vs.* β) and the C-4 configuration on anticancer efficacy.

**Table 6:** Cytotoxicity of podophyllotoxin glycosides across various cancer cell lines

Compound no.	IC_50_ (µM)				
	HL-60	SMMC-7721	A-549	MCF-7	SW480
27a	>40	>40	>40	>40	>40
27b	0.14	0.62	0.61	1.27	1.65
27c	>40	>40	>40	>40	>40
27d	5.32	15.21	7.62	13.48	16.83
27e	0.6	0.78	0.61	1.42	1.11
27f	>40	>40	>40	>40	>40
27g	>40	>40	>40	>40	>40
28a	16.87	16.82	16.04	39.13	38.71
28b	4.89	3.78	5.7	5.67	10.65
28c	2.71	3.68	3.1	7.5	4.85
28d	2.54	2.68	3.52	4.71	5.05
28e	26.49	17.1	23.15	29.32	>40
28f	>40	>40	>40	>40	>40
28g	9.59	20.24	18.64	>40	>40
30a	0.61	0.83	1.12	1.69	1.26
30b	3.15	18.09	17.81	22.11	26.52
Etoposide	0.31	8.12	11.92	32.82	17.11
Cisplatin	1.17	6.43	9.24	15.86	13.42

## 8. Synthesis of pyranopyrazolo *N*-glycosides and pyrazolopyranopyrimidine *C*-glycosides

**Scheme 12:**
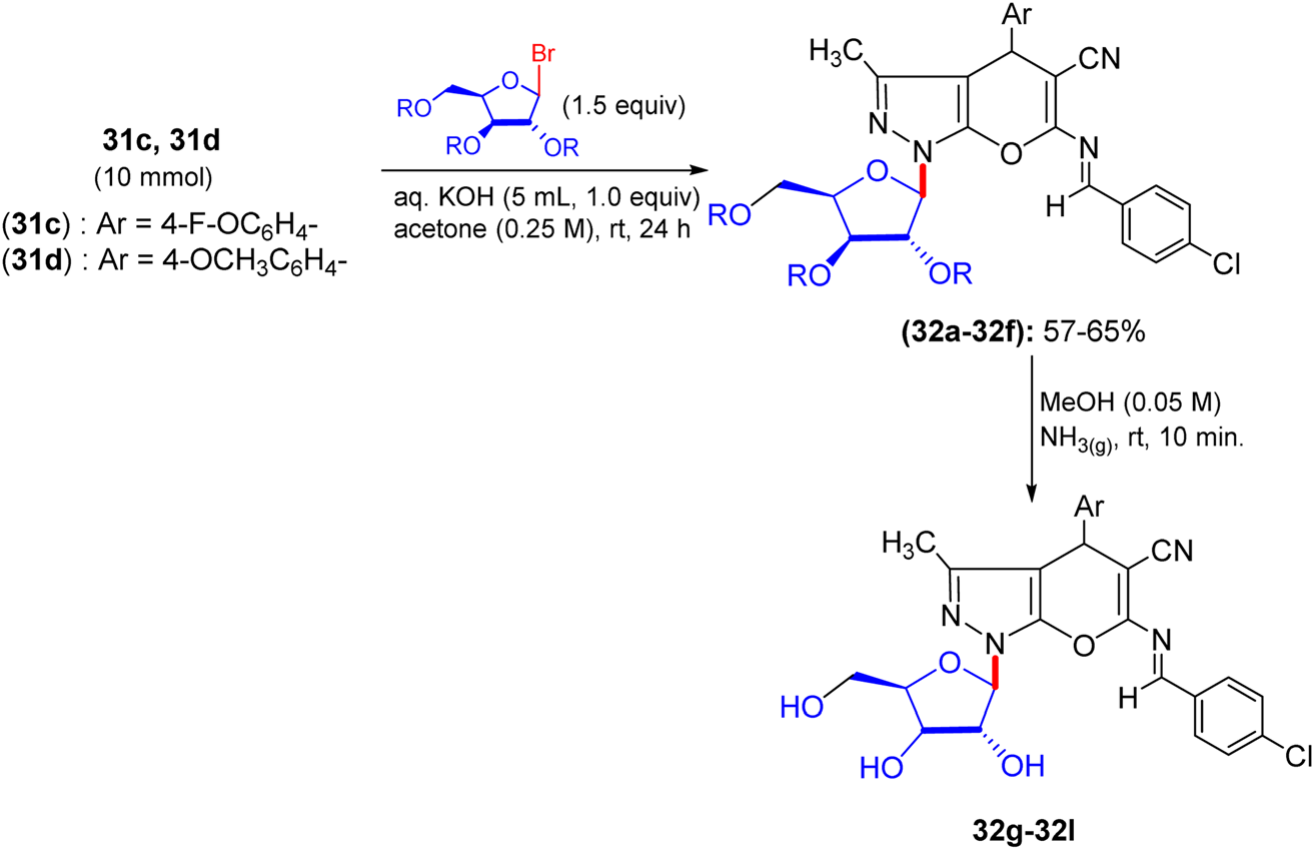
Synthesis of deacetylated nucleosides 32g–32l.

**Scheme 13:**
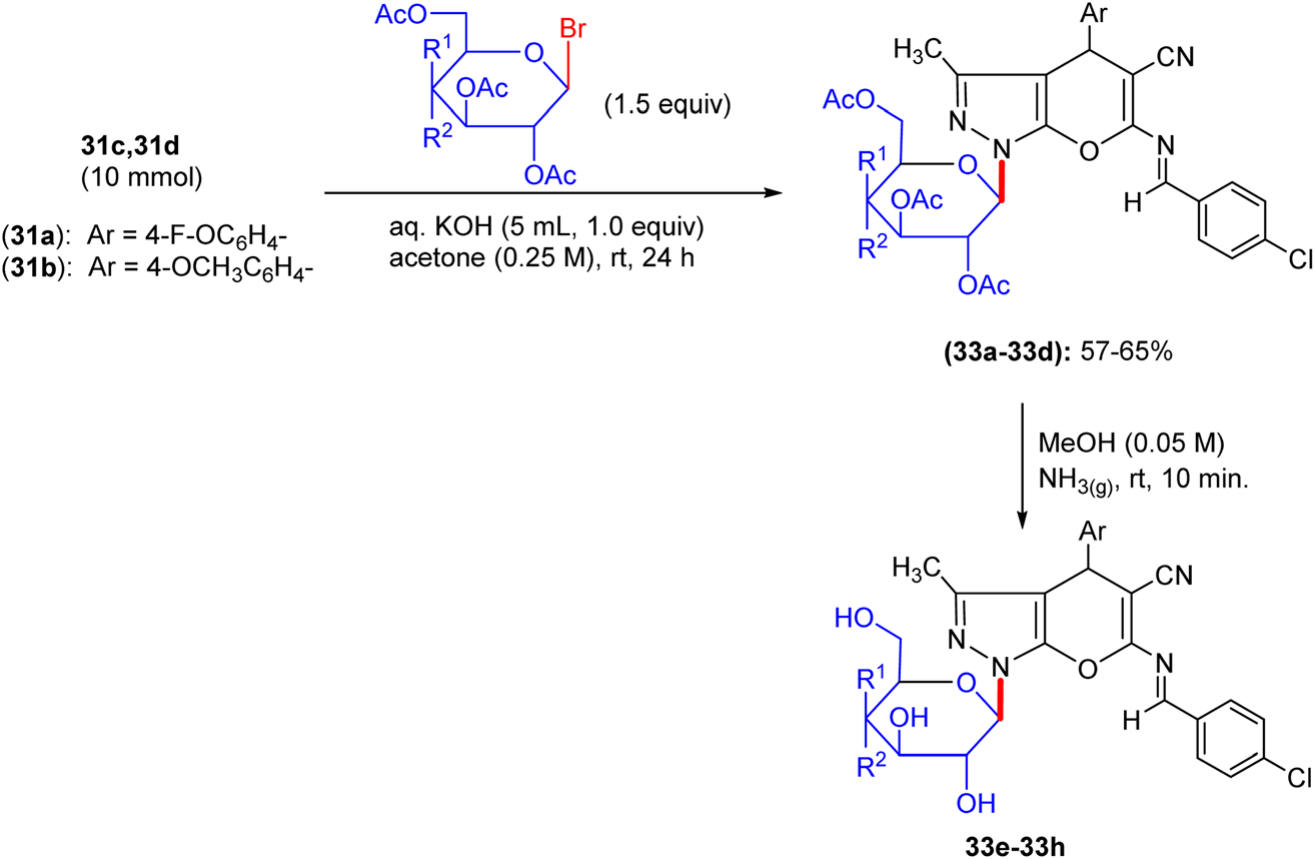
Synthesis of deacetylated nucleosides 33e–33h.

Shortly after the report by Jiang in the same year, El-Gazzar *et al.* proposed a synthetic scheme for pyranopyrazolo *N*-glycoside and pyrazolopyranopyrimidine *C*-glycoside derivatives.^46^ They prepared precursors 31a and 31b, which on glycosylation yielded acyclic nucleosides 32a–32f and 33a–33d, respectively (Schemes 12 and 13). Elemental analyses and spectral data confirmed the structure of the nucleosides. The anomeric proton of the glucose moiety in the ^1^H NMR spectrum of compound 33a appears as a doublet at *δ* 6.03 ppm with coupling constant *J* = 10.70 Hz, indicating the β-configuration at the anomeric centre (Scheme 13).

The deprotection of acyclic nucleosides 32a–32f and 33a–33d was done by stirring in methanolic ammonia solution at ambient temperature, affording the corresponding deacetylated nucleosides 32g–32l and 33e–33h, respectively (Scheme 12). In addition, spectral data and elemental analyses confirmed the structures of the free nucleosides. Specifically, the ^1^H NMR spectrum of compound 32g featured the anomeric proton as a doublet at 6.88 ppm.

**Scheme 14:**
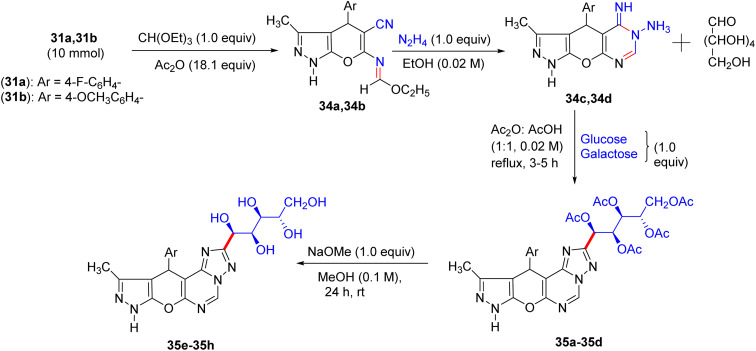
Synthesis of compounds 35e–35h.

**Scheme 15:**
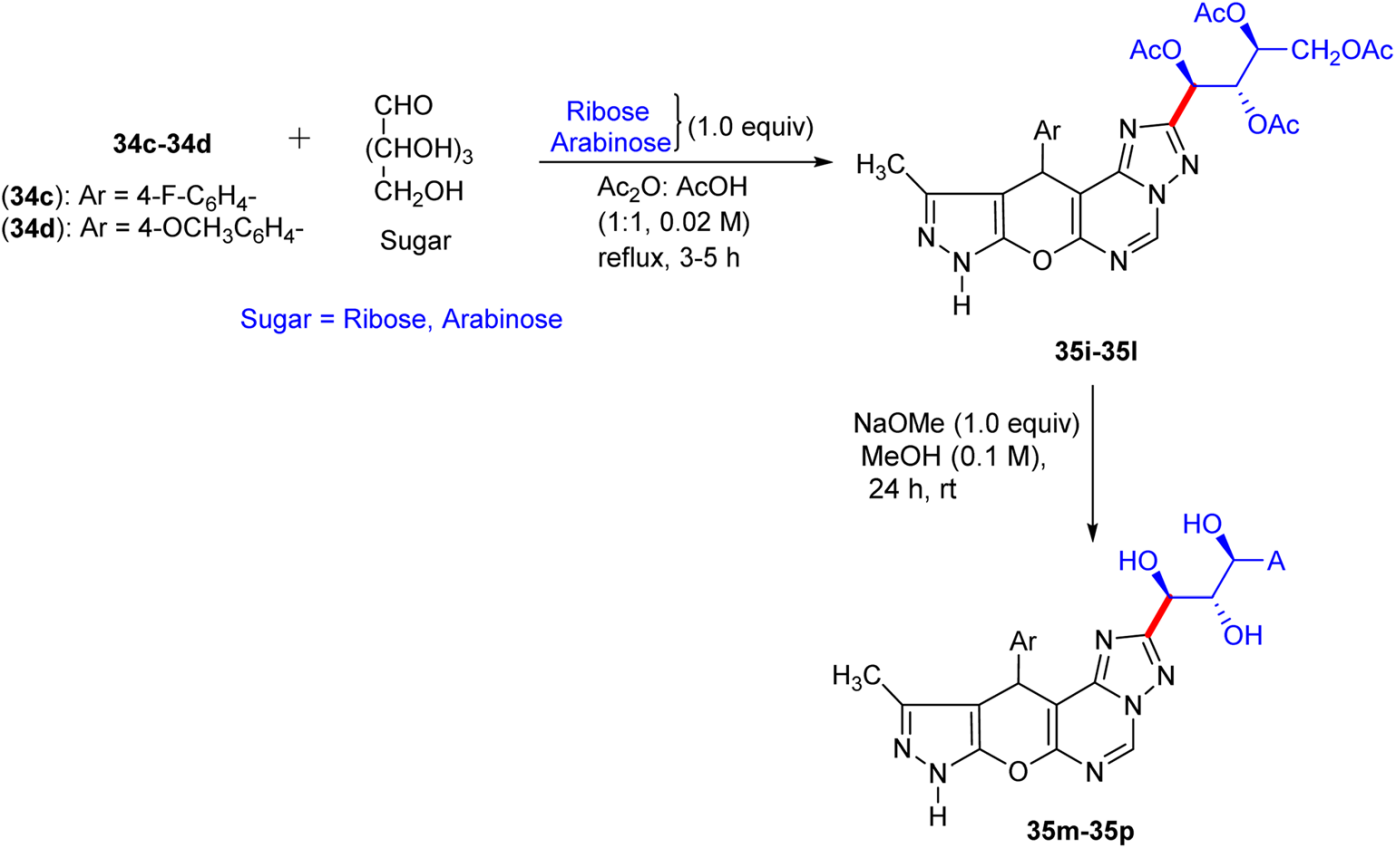
Synthesis of compounds 35m–35p.

Compounds 31a and 31b were reacted with triethylorthoformate and acetic anhydride on heating to form pyrano[2,3-*c*]pyrazole imidoformate derivatives 34a and 34b, which on cyclization with hydrazine hydrate gave the three fused rings compounds 34c and 34d, respectively. The prepared compounds were afterwards heated with aldohexoses (d-glucose and d-galactose) or aldopentoses (d-ribose and d-arabinose) in acetic anhydride-acetic acid, producing the corresponding intermediates 35a–35d and 35i–35l, respectively. Deprotection of the intermediates in methanolic sodium methoxide solution afforded (pyrano[3,2-*e*][1,2,4]triazolo[1,5-*c*]pyrimidin-2-yl) butane-1,2,3,4-tetraol derivatives 35e–35h and 35m–35p in excellent yields (Scheme 14 and 15), respectively.

### 8.1. Findings through *in vitro* growth analysis and binding characteristics of compounds

Cytotoxicity tests for compounds 32g, 32h, 32i, 33e, 33h, 35e, and 35m were carried out with three human cancer cell lines, *viz.* HepG2 (liver cancer), HT29 (colon cancer), and MCF-7 (breast adenocarcinoma), in a dose-dependent mode (refer to the results in Table 7).^46^ The control drug doxorubicin displayed penetrating cytotoxicity in accordance with the outcomes of the compounds 32g and 32h. The high cytotoxicity activity of 32g and 32h can be attributed the incorporation of *N*-(β-d-ribofuranosyl), *N*-(β-d-xylofuranosyl), and 4-fluorophenyl in the pyrane ring, as directed by the structural analyses, reflecting that the presence of *N*-furan prompted compounds 32g and 32h to illustrate higher activity than 33e and 33h, respectively. Furthermore, compound 35m (derived from aldopentoses) turned out to be much more active than compound 35e (derived from aldohexose).

**Table 7:** Cytotoxic effects of the synthesized compounds against the HePG2, HT29, and MCF-7 tumor cell lines

Compound no.	GI_50_ (µmol L^−1^)		
	HePG2	HT29	MCF-7
32g	5.8	3.9	6.2
32h	6.7	4.5	6.8
32j	14.5	11.9	16.7
33e	11.9	20.3	15.2
33h	20.6	22.3	24.1
35e	25.6	20.5	23.2
35m	18.6	24.3	20.3
Doxorubicin	0.05	0.09	0.05

The structure–activity relationship (SAR) and docking analyses indicated that *N*-furanosyl and 4-fluorophenyl substitutions in compounds 32g and 32h enhanced their binding affinity to mitochondrial proteins, forming hydrogen bonds with residues such as Ser184 of Bax (*K*_d_ ∼ 1–5 µM), respectively. These interactions facilitate the generation of reactive oxygen species (ROS) and induce mitochondrial apoptosis, as corroborated by enzyme-linked immunosorbent assay (ELISA) results. Furthermore, micro-Fourier transform infrared (micro-FTIR) spectroscopy and elemental analysis confirmed the incorporation of sugar and fluorine, thereby supporting their structural contributions to the binding affinity.

## 9. Synthesis of curcumin glycosides

A study on the anticancer activity of curcumin glycosides by Sohng in 2017 envisaged the development of curcumin derivatives with enhanced physiological properties through glycosylation. UDP-α-d-glucose and UDP-α-d-2-deoxyglucose were articulated by the OPME chemoenzymatic system, and were then screened by UFLC-DAD (ultra-fast liquid chromatography-diode array detector) and ESI/MS. Curcumin monoglucoside (Cmg) and curcumin diglucoside (Cdig) were the two main products, designed by attaching the corresponding one and two glucosyls to curcumin aglycone and purified to extend the investigation.

**Fig. 2:**
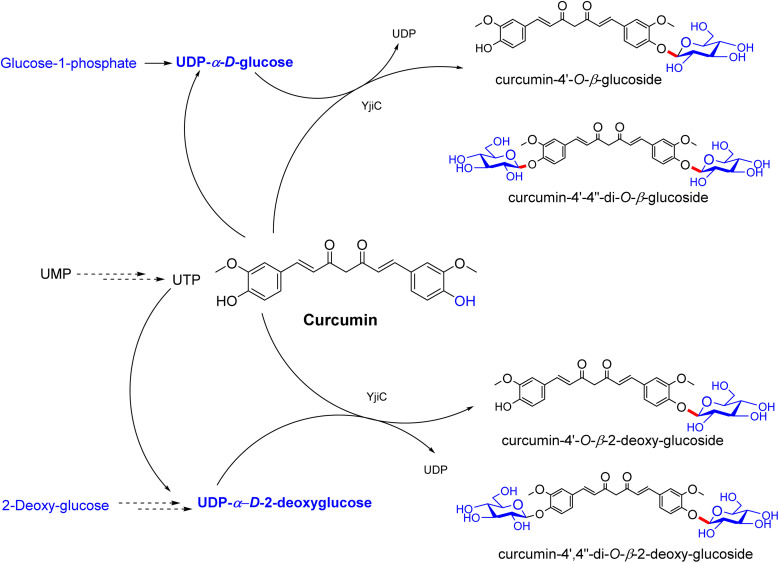
Schematic of the synthesis of curcumin glycosides and curcumin 2-deoxyglycosides through one-pot multi-enzyme reactions.

The OPME chemoenzymatic reaction is discriminatory for furnishing the 2-deoxyglucose of curcumin.^47^ The two products formed during the OPME chemoenzymatic synthesis act as substrate molecules with two hydroxyl groups. These reactions were carried out on a large scale and supplied ample quantities of the products, which were characterized and assessed with ease. Although this system is cost-effective, it can recycle UDP and regenerate ATP (Fig. 2). Both systems were combined with a resilient UDP-glycosyltransferase (UGT) (extracted from *Bacillus licheniformis*) (modification). However, the formation of products in OPME reaction systems can be regulated by adjusting the concentration of substrate molecules and pioneering materials for sugar nucleotide donors. It was corroborated by surveys that an elevated substrate concentration favored the development of monoglycosides, even though lower substrate concentrations resulted in the formation of diglycosides. The OPME arrangements sustained their production by magnificently glycosylating α-mangostin and nargenicin.

### 9.1. Binding characteristics and mechanisms of action of curcumin glycosides in anticancer activity

Curcumin glycosides were tested in tumour cell lines (MCF-7, HepG2, and A549), and the results are shown in Table 8, revealing that growth inhibition is both dose- and time-dependent. Curcumin 4′-*O*-β-glucoside (Cmg) and curcumin 4′-*O*-β-2-deoxyglucoside (Cdg a) exhibited notable cytotoxicity (IC_50_ ∼ 5–12 µM), surpassing that of curcumin (IC_50_ ∼ 10–20 µM), with Cmg being the most potent. Docking studies indicated that Cmg binds to NF-κB and mitochondrial Bax (*K*_d_ ∼2–5 µM), with the β-glucoside forming hydrogen bonds with Ser536 and Asp residues, enhancing solubility and specificity.^47^ Cdg showed slightly weaker binding (*K*_d_ ∼ 3–6 µM) due to the lack of the C-2 hydroxyl group, as confirmed by molecular dynamics. Diglycosides were less effective (IC_50_ ∼ 15–30 µM), with docking studies showing reduced affinity (*K*_d_ ∼ 8–15 µM) due to steric hindrance from multiple sugar units. SAR analyses highlighted the significance of β-glucoside in boosting the binding and potency, with monoglycosides outperforming the bulkier diglycosides. Micro-FTIR confirmed the presence of glycosidic bonds (C–O stretch ∼1000–1100 cm^−1^), UFLC-DAD verified their purity, and molecular imaging showed their cytosolic/mitochondrial localization. The mechanism of action involves NF-κB/AP-1 inhibition, ROS generation, and mitochondrial apoptosis, with glucosylation-enhancing their activity and specificity in the target cells.

**Table 8:** Cytotoxicity of curcumin glycosides

Compound	IC_50_ (µM)					
	AGS	HCT116	HepG2	HeLa	U87MG	B16F10
Cur	9.77	5.51	36.77	22	7	7.49
Cmg	7.11	5.27	41.94	25	23.32	21.6
Cdig	18.09	17.3	>100	93	99.1	66.57
Cdg a	5.86	5.4	18.16	12	19.9	18.6
Cdg b	6.9	10.35	13.04	13	1077	20.86

## 10. Synthesis of pyrimidine and triazolopyrimidine glycosides

**Scheme 16:**
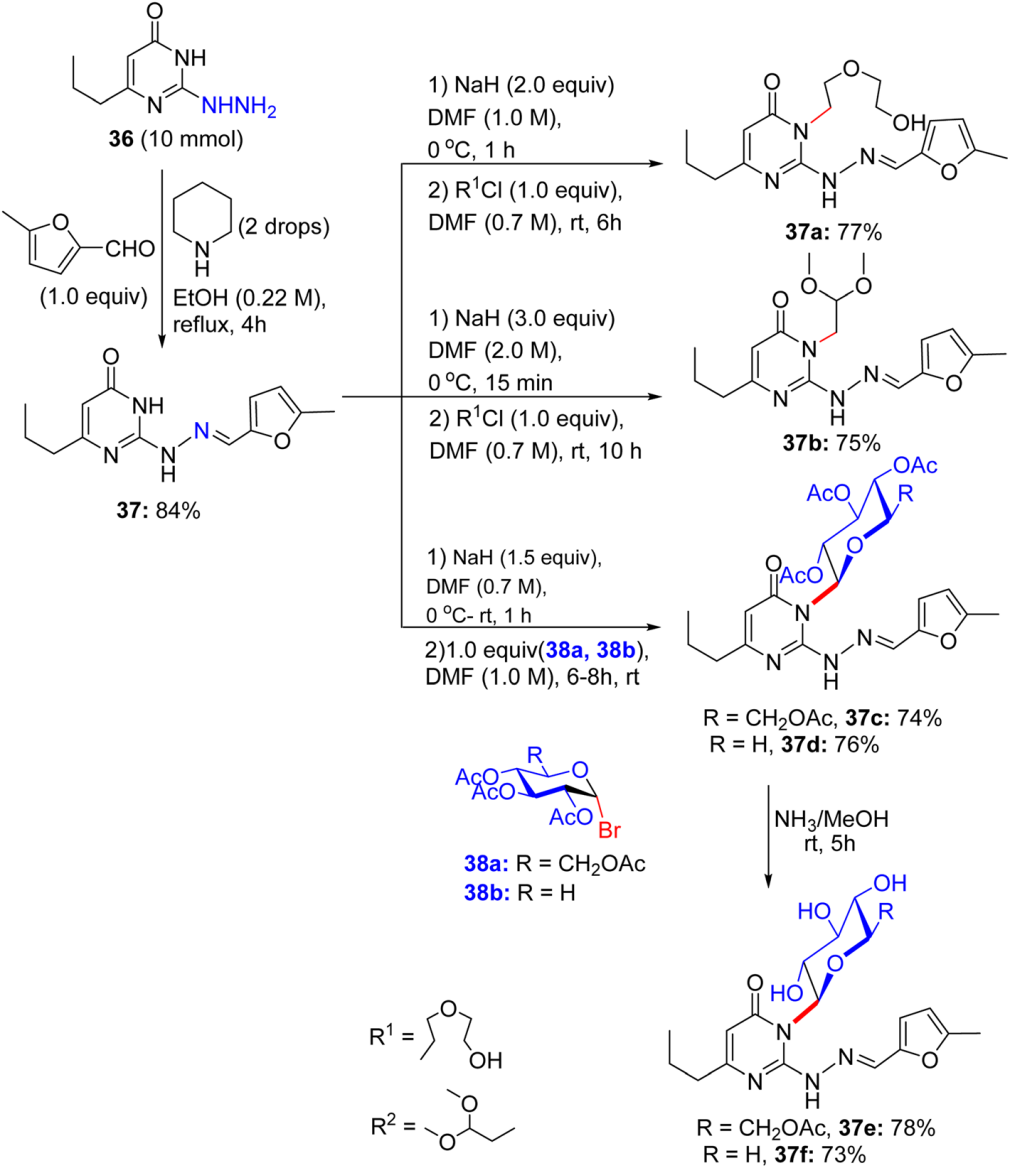
Synthesis of pyrimidine glycosides and acyclic analogs.

Later, in 2017, El-Sayed and Mohamed took advantage of the pre-existing methods and synthesized 2-hydrazine pyrimidine derivative 36, which turned out to be the basis for the formation of free hydroxyl glycoside derivatives 39e and 39f.^48^ The starting material 36 was reacted with 5-methylfurfural in the presence of ethanolic acetic acid to afford (furylmethylene)hydrazinyl derivative 37, and then it was modified to access pyrimidine *N*-substituted acyclic oxygenated alkyl compounds and *N*-glycosides. The reaction of substituted pyrimidine 37 with 2-(2-chloroethoxy)ethan-1-ol and 2-chloro-1,1-dimethoxyethane with the assistance of sodium hydride in *N*,*N*-dimethylformamide for 6 h and 10 h resulted in the construction of *N*^3^-substituted pyrimidine compounds with acyclic oxygenated alkyl chain, 37a in 77% and 37b 75% yields, respectively (Scheme 16). Substituted pyrimidine derivative 37 proceeded the remaining sequence by reacting with *O*-acetylated glycopyranosyl bromide 38a and 38b for 5 h in the presence of sodium hydride under ambient temperature, supplying the acetylated *N*^3^-pyrimidine glycosides 37c or 37d, respectively, in an accessible 74% and 76% yields, respectively. Deacetylation of the products was accomplished in saturated methanolic ammonia at near room temperature, providing deacetylated glycosides 37e and 37f, respectively.

**Scheme 17:**
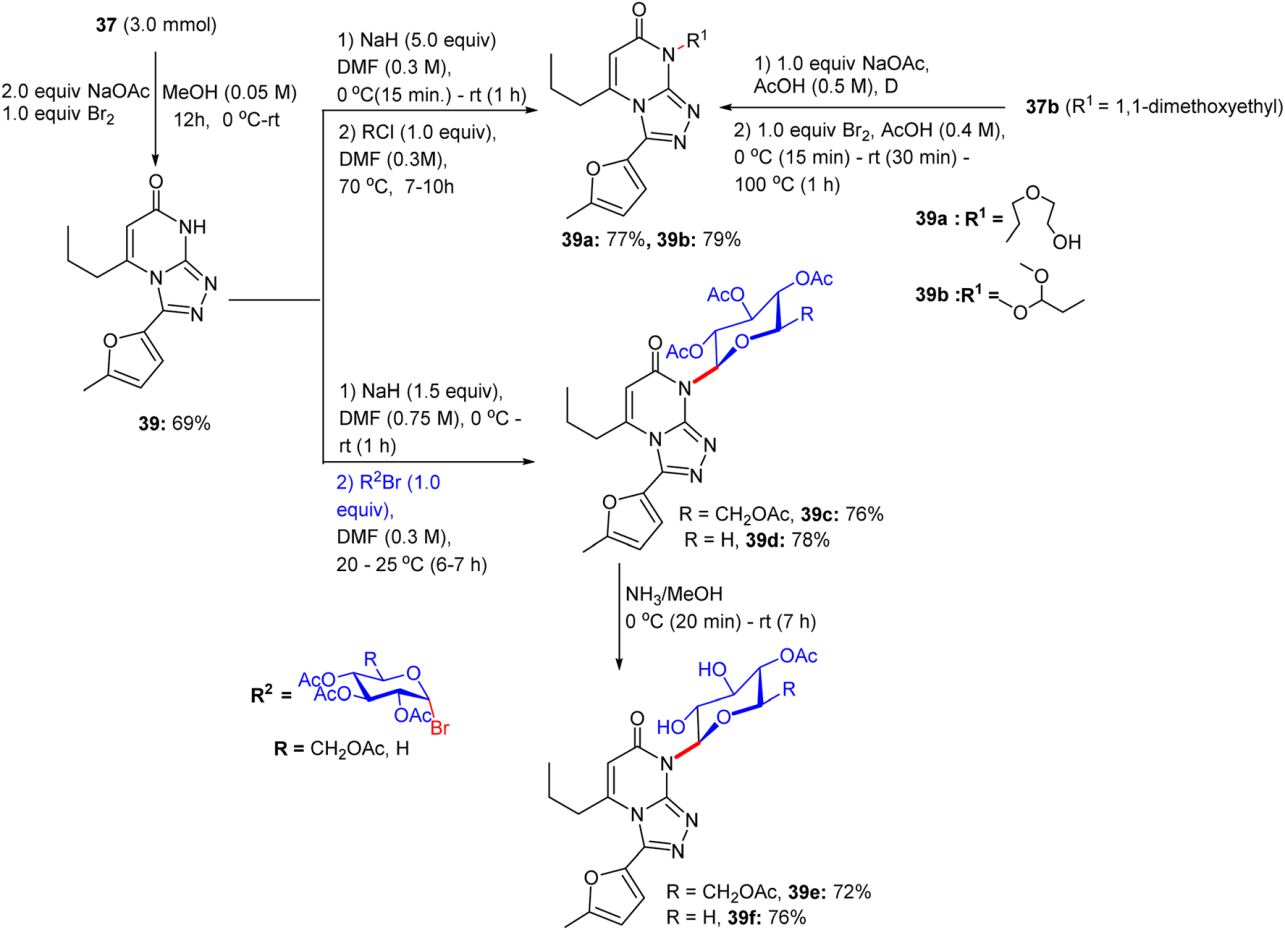
Synthesis of triazolopyrimidine glycosides.

Subsequently, the abridged pyrimidine derivative with a free NH in the pyrimidine core of compound 37 was reacted with bromine/NaOAc in methanol for 12 h at room temperature, giving [1,2,4]triazolo[4,3-*a*]pyrimidine derivative 39 in excellent yield (Scheme 17). An additional *N*-substituted triazolopyrimidine derivative 39a was prepared in 77% from triazolopyrimidine system 39. At this point, triazolopyrimidine system 39 was glycosylated with α-glycosyl bromides in sodium hydride and *N*,*N*-dimethylformamide solution for 7–10 h and 6–7 h to develop the corresponding *N*^3^-triazolopyrimidine glycosides 39c and 39d, respectively. The presence of alkyl, aryl, and sugar protons was confirmed by ^1^H NMR spectra, indicating the existence of a β-glycosidic linkage in glycosides 39c and 39d, respectively. As a result, the deacetylation of the acetylated *N*-glycosides using ammonia solution in dehydrated methanol at room temperature within 7 h formed the free hydroxyl glycoside derivatives 39e and 39f, respectively. IR spectral analyses confirmed the presence of hydroxyl bands and the weakening of the acetyl-carbonyl bands in these deprotected glycosides.

### 10.1. Anticancer activity and mechanism of action of pyrimidine glycosides and triazolopyrimidine derivatives

The anticancer potential of the compounds was evaluated using a panel of human cancer cell lines (PC3, MCF-7, and HepG2), and the results are presented in Table 9 and 10. Wael and Ashraf reported that *N*-substituted pyrimidine glycosides (39d and 39e) and triazolopyrimidine glycosides (39 and 39b) exhibited moderate activity (IC_50_ ∼ 8–30 µM), with 39 showing low toxicity in normal cells (IC_50_ > 50 µM), indicating their cancer cell specificity.^48^ Docking studies revealed that 37d and 37e bind to thymidylate synthase (TS) and dihydrofolate reductase (DHFR), respectively, with glycosyl moieties (glucose/xylose) forming hydrogen bonds with Asp218 or Ser residues (*K*_d_ ∼ 5–10 µM), thereby enhancing their solubility and specificity. Triazolopyrimidine 39 binds TS or EGFR (*K*_d_ ∼ 3–8 µM), with the triazole ring enabling π–π stacking with Phe and the glycoside forming H-bonds with Lys. The N-1 acyclic oxygenated alkyl in 39b enhanced the binding (*K*_d_ ∼ 2–6 µM) and potency in PC3 cells through additional hydrogen bonding with Ser. SAR analyses highlighted the glycosyl and triazole enhancements, with the glucose/xyloside glycosides outperforming their non-glycosylated analogues. Micro-FTIR confirmed the presence of glycosidic and triazole bonds (C–O ∼ 1000–1100 cm^−1^ and C=N ∼ 2100 cm^−1^), UFLC-DAD verified their purity, and molecular imaging showed their cytosolic localization. MoA is involved in TS/DHFR or EGFR inhibition, which disrupts DNA synthesis or signalling, leading to apoptosis. These binding characteristics suggested that pyrimidine nucleoside analogues are promising anticancer agents, warranting further design and synthesis studies.

**Table 9:** IC_50_ values in PC3, HCT116, MCF7, and RPE1 cancer cell lines

Compound no.	Cytotoxicity			
	PC3	HCT116	MCF7	RPE1
37	31%	8%	13%	27%
37d	55%	42%	18%	53%
37e	62%	55%	31%	47%
39	48%	40%	46%	26%
39b	515%	14%	31%	55%

**Table 10:** Cytotoxicity data for compounds showing 40% inhibition

Compound no.	PC3		HCT116		MCF7		RPE1	
	IC_50_	IC_90_	IC_50_	IC_90_	IC_50_	IC_90_	IC_50_	IC_90_
37d	75	149	93	155	—	—	66	146.9
37e	70	123	95	144	—	—	93	172
39	98	138	101	169	103	160	—	—
39b	87	177	—	—	—	—	148	245
DOX	6.8	13.8	2.2	5.2	12.8	51.7	—	—

## 11. Synthesis of anthracycline-based glycoconjugates

**Scheme 18:**
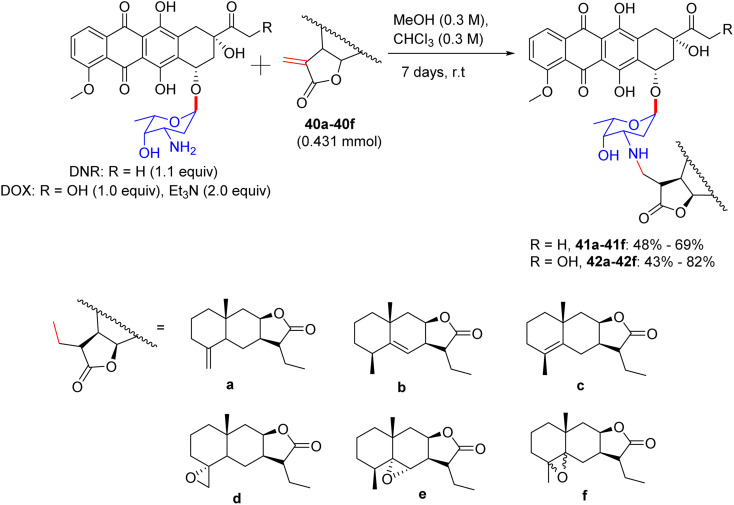
Synthesis of anthracyclin conjugates.

In 2018, a new approach was presented by Semakov in their work on the modification of anthracycline antibiotics, presumably daunorubicin (DNR) and doxorubicin (DOX), with the given sesquiterpene lactones isoalantolactone 40a, alantolactone 40b, alloalantolactone 40c (separated from the elecampane plant extract), and its epoxy derivatives 40d–40f. These compounds were separated from the chloroform extract.^49^ Alloalantolactone 40c was isolated in ample quantities by the isomerization of isoalantolactone in an acidic medium (AcOOH and CF_3_COOH). These lactones can interact with numerous nucleophiles by Michael reaction. The amino group of anthracycline antibiotics with carbohydrate units worked as amines. The acquired amine group was reactive but was easily separated. Doxorubicin was consumed as hydrochloride with sesquiterpene lactones in the presence of a base in CHCl_3_, which altered the doxorubicin hydrochloride into the free amine *via* Michael aza-reaction in 7 days at room temperature, and these free amines can directly react with lactone. Sesquiterpene lactones 40a–40f were then reacted with daunorubicin and doxorubicin under milder conditions to afford adducts (41a–41f and 42a–42f) of the anthracycline antibiotics (DNR or DOX) and sesquiterpene lactones in good to excellent yields (Scheme 18). The lipophilicity and vividness in colors of the formed adducts enable their chromatographic purification.

### 11.1. Findings through MTT assay and cytotoxicity, and mechanism of action of sesquiterpene-lactone anthracycline glycoconjugates

Evidence suggests that sesquiterpene-lactone anthracycline glycoconjugates exhibit significantly greater cytotoxicity towards daunorubicin than doxorubicin across various cancer cell lines (Table 12). Notably, the epoxyisoalantolactone–daunorubicin conjugate (41d) demonstrated enhanced cytotoxicity in HCT116 cells (IC_50_ ∼ 0.5–2 µM) compared to daunorubicin, effectively overcoming the resistance in daunorubicin-resistant cells.^49^ Docking studies indicate that 41d binds to topoisomerase II (Topo II, *K*_d_ ∼0.1–1 µM) through the α-l-daunosamine moiety of daunorubicin, forming hydrogen bonds with Arg503 and Gln778, and to P-glycoprotein (P-gp, *K*_d_ ∼ 1–3 µM) *via* the epoxide group of epoxyisoalantolactone, establishing hydrogen bonds with Tyr310 and hydrophobic interactions with Phe983, thereby inhibiting drug efflux. Structure–activity relationship (SAR) analyses highlighted the synergistic interaction between the epoxide of 41d and daunosamine, enhancing its affinity for Topo II and P-gp, with the stereochemistry of α-l-daunosamine optimizing the hydrogen bonding. Micro-FTIR analysis confirmed the presence of epoxide (C–O–C ∼1250 cm^−1^) and glycosidic bonds (C–O ∼ 1000–1100 cm^−1^), while UFLC-DAD verified its purity, and molecular imaging demonstrated its nuclear and membrane localization. Its mechanism of action involves Topo II inhibition, stabilization of DNA strand breaks, P-gp blockade, and sustained toxicity in resistant cells, underscoring the potential of 41d to overcome daunorubicin resistance (Table 11).

**Table 11:** Cytotoxic activity (in µM) for anthracycline conjugates

Compound no	IC_50_ (µM)				
	A549	HCT116	MCF7	RD	HEK293
40a	32.04	11.37	17.51	10.37	74.03
40b	36.73	10.57	13.5	5.48	36.47
40c	23.12	34.52	17.92	8.82	35.87
40d	83.51	21.4	31.87	18.6	105.68
40e	21.49	5.12	11.41	4.81	38.1
40f	50.28	9.75	24.03	8.77	18.47
41a	0.93	0.28	3.95	0.96	3.43
41b	0.66	0.09	3.07	0.71	6.23
41c	1.42	1.01	1.24	0.59	6.37
41d	0.27	0.02	1.99	0.63	11.41
41e	1.19	1.3	1.84	0.8	18.19
41f	0.56	0.26	1.47	0.41	3.68
42a	2.21	2.18	11.11	2.41	19.82
42b	3.29	4.55	26.26	2.67	29.83
42c	3.18	1.26	8.14	2.98	11
42d	1.86	1.07	5.65	2.76	11.73
42e	4.52	2.52	3.03	2.47	4.5
42f	0.88	0.25	2.94	1.18	0.98
DNR	0.33	0.21	1.44	2.45	11.17
DOX	0.38	0.14	0.46	0.29	6.78

**Table 12:** Antiproliferative activity of the anthracyclin conjugates at different time intervals

Compound	IC_50_ (µM)		
	K562		
	24 h	48 h	72 h
DNR	23.48	0.74	0.42
L04-DNR	7.83	2.48	0.69

## 12. Synthesis of flavonoid triazol-glycosides

**Scheme 19:**
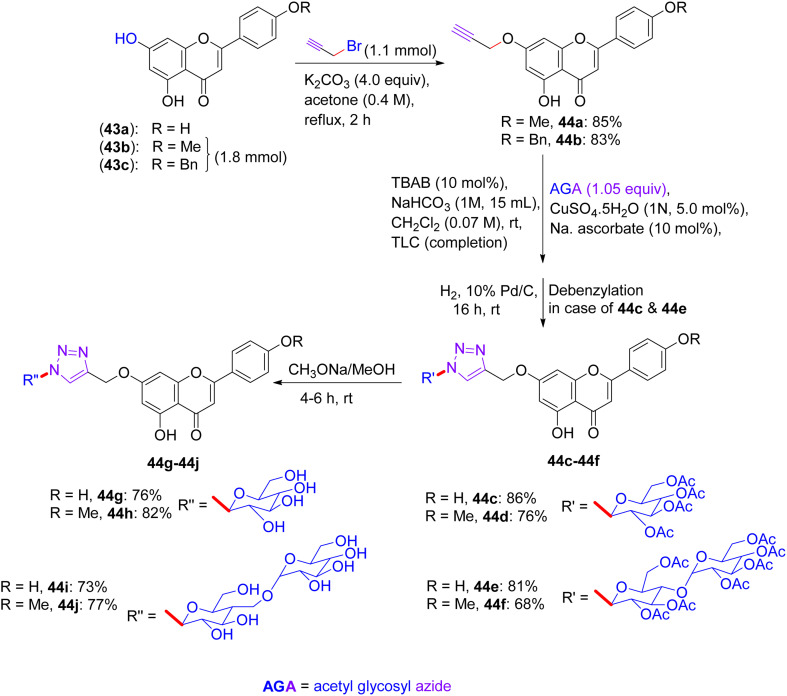
Synthesis of flavonoids and their triazolyl glycosides 44c–44j.

**Scheme 20:**
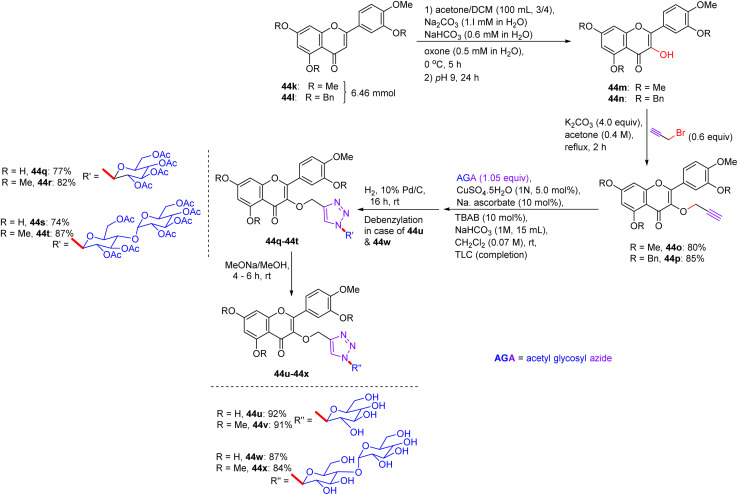
Synthesis of acetylenic flavonoids and their triazolyl glycosides 44q–44x.

In Feb 2018, Wang reported the synthesis of flavonoid triazolyl glycosides.^50^ Click chemistry was used as a synthetic tool. Starting acetylenic flavonoids 44a, 44b, 44o, and 44p and acetylated sugar azides were prepared using known procedures (Scheme 19 and 20). Then, the copper-catalyzed CuAAc reaction of acetylated sugar azides and acetylenic flavonoids and subsequent deacylation afforded the desired flavonoid sugars (44g–44j and 44u–44x). The azide–alkyne cycloaddition reactions gave the triazolyl glycosides in β-configuration exclusively. The desired compounds were confirmed by the large axial–axial coupling constant of 8.0–9.2 Hz linking anomeric atoms H (1) and H (2) in their ^1^H NMR spectra.

### 12.1. Findings through cell counting kit-8 (CCK-8) assay and binding characteristics of compounds

The *in vitro* antitumour potency of flavonoids (43a, 43b and 44m) and novel flavonoid triazolyl glycosides (44c–44j and 44q–44x) was evaluated using the cell counting kit-8 assay in three human cancer cell lines (MCF-7, HepG2, and A549), with cisplatin as a positive control (Table 13). Linear regression analysis of the concentration–response curves revealed significantly higher antitumor activity for triazolyl glycosides (IC_50_ ∼ 0.5–5 µM) compared to cisplatin (IC_50_ ∼ 10–20 µM) and flavonoids (IC_50_ ∼ 5–15 µM).^50^ Docking studies show that 44f (glucose-triazole) and 44q (xylose-triazole) bind EGFR or Bax (*K*_d_ ∼ 1–3 µM), with the triazolyl glycoside forming H-bonds with Lys721 or Ser184, and the flavonoid core engaging in π–π stacking with Phe residues. The triazole linker enhances their affinity through additional H-bonds and π–π interactions, whereas glycosides improve their solubility and GLUT-mediated uptake. Flavonoids 43a–43b and 44m bound less effectively (*K*_d_ ∼ 3–8 µM) because of the lack of glycosides. SAR analyses reveal glucose/xylose and triazole synergy, with B-ring hydroxyls modulating the potency. Micro-FTIR confirmed the presence of triazole (C=N ∼ 2100 cm^−1^) and glycosidic bonds (C–O ∼ 1000–1100 cm^−1^), UFLC-DAD verified their purity, and molecular imaging showed their cytosolic/mitochondrial localization. MoA involves EGFR/PI3K inhibition and Bax-mediated mitochondrial apoptosis, surpassing the damaging effects of cisplatin.

**Table 13:** IC_50_ values for the flavonoid triazolyl glycoside compounds against the HeLa, HCC1954, and SK-OV-3 cell lines

Compound no.	IC_50_ (µM)		
	Hela	HCC1954	SK-OV-3
43a	8.4	13.4	42.54
43b	44.51	39.37	>100
44m, 44c–44f	>100	>100	>100
44f	14.67	>100	>100
44g–44j	>100	>100	>100
44q	36.67	30.56	43.42
44r–44t, 44u	>100	>100	>100
44v	53.33	39.79	>100
44w–44x	>100	>100	>100
Cisplatin	21.3	33.57	12.07

## 13. Synthesis of pyrazole and pyrazolopyrimidine glycosides

**Scheme 21:**
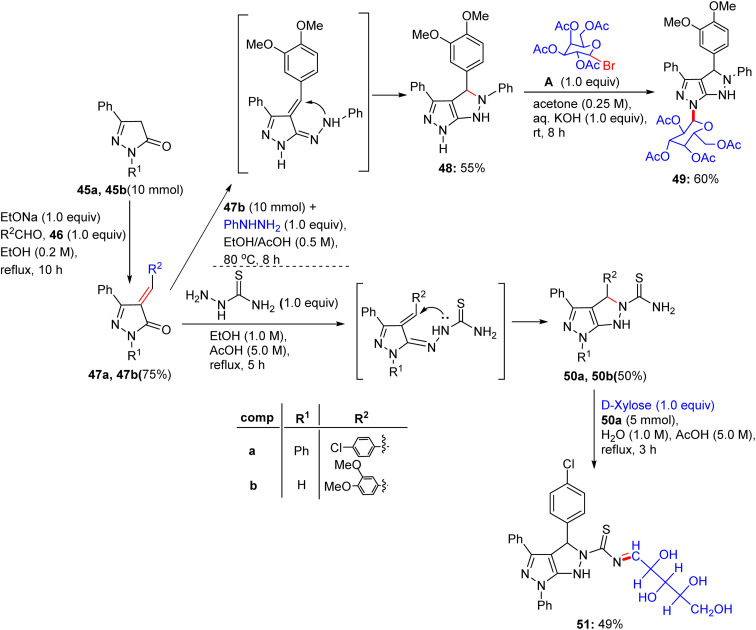
Synthesis of fused pyrazole compounds 49–51.

Later on, in 2018, Nassar and coworkers extended the scope of their previous work^51*a*^ to a pathway for converting pyrazolones into new derivatives with cyclic and acyclic glucose units (Scheme 21). Pyrazolone derivatives 45a and 45b were reacted with 4-chlorobenzaldehyde 46a and 3,4-dimethoxybenzaldehyde 46b in the presence of sodium ethoxide solution to produce the compounds 4-(4-chlorobenzylidene)-2,5-diphenyl-2,3-dihydro-3*H*-pyrazol-3-one 44a and 4-(3,4-dimethoxybenzylidene)-5-phenyl-2,3-dihydro-3*H*-pyrazol-3-one 47b by Claisen–Schmidt condensation reaction, respectively.^51*b*^ The reaction of compound 47b with phenylhydrazine in ethanol and acetic acid generated *N*-phenylpyrazolo[3,4-*c*]pyrazole derivative 48 as a cyclized product. Compound 48 was then cleanly reacted with 2,3,4,6-tetra-*O*-acetyl-α-d-glucopyranosyl bromide A in acetone and aqueous KOH, furnishing *N*-glucoside derivative 49. However, compounds 50a and 50b were condensed and cyclized with thiosemicarbazide in ethanol and acetic acid under reflux conditions to produce pyrazolo[3,4-*c*]pyrazole-2(1*H*)-carbothioamide derivatives 50a and 50b, respectively. Then, compound 50a was glycosylated by means of d-xylose with the assistance of ethanolic acetic acid solution, yielding the desired sugar carbothioamide derivative 51.

**Scheme 22:**
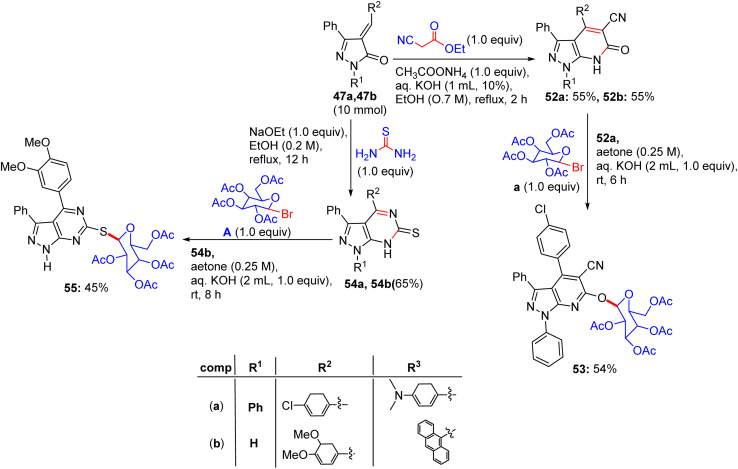
Synthesis of compounds 53–55.

Chalcone derivatives 47a and 47b were correspondingly combined with ethyl cyanoacetate in ammonium acetate by cyclization process, generating pyrazolo[3,4-*b*]pyridine-5-carbonitrile derivatives 52a and 52b, respectively (Scheme 22). Compound 52a can be condensed with 2,3,4,6-tetra-*O*-acetyl-α-d-glucopyranosyl bromide to derive compound 53 on elimination. Compounds 47a and 47b were lastly reacted with thiourea in KOH solution using the same previously mentioned method and provided pyrazolopyrimidine derivatives 54a and 54b, respectively. Finally, compound 54b was reacted with 2,3,4,6-tetra-*O*-acetyl-α-d-glucopyranosyl bromide under the optimized glycosylation conditions to afford the corresponding pyrazolopyrimidine glycoside 55.

### 13.1. Findings through MTT assay and binding characteristics of compounds

Nine derivatives were explored for their antitumour potency against MCF-7 cells, with glycosides 50a and 52 (pyrazole and pyrazolopyrimidine glycosides, respectively) exhibiting superior activity (Table 14). Fathy M. Abdelrazek *et al.* reported dose-dependent activity, with 50a and 51 surpassing doxorubicin (IC_50_ ∼ 2–5 µM) with IC_50_ values of ∼0.5–2 µM.^51*a*^ Docking studies revealed that 50a binds EGFR or topoisomerase II (Topo II, *K*_d_ ∼ 1–3 µM), with its β-d-glucose forming H-bonds with Lys721 or Arg503, and the pyrazole core enabling π–π stacking with Phe residues. Compound 51, a pyrazolopyrimidine glycoside, exhibited similar binding (*K*_d_ ∼ 1–3 µM), with the fused pyrimidine ring enhancing its π–π interactions, thereby improving its affinity over compound 50a. SAR analyses highlighted the glycosyl (glucose/xylose) synergy, which boosts their solubility and GLUT-mediated uptake, with the rigidity of pyrazolopyrimidine optimising their potency. Micro-FTIR confirmed the presence of glycosidic (C–O ∼ 1000–1100 cm^−1^) and pyrazole/pyrimidine bonds (C=N ∼ 1500 cm^−1^), UFLC-DAD verified their purity, and molecular imaging showed their cytosolic/nuclear localization. MoA involves EGFR or Topo II inhibition, reducing proliferation or stabilising DNA breaks, leading to apoptosis, and surpassing the DNA-damaging effects of doxorubicin. These binding characteristics of 50a and 51 position them as promising anticancer agents.

**Table 14:** Antiproliferative activity of compounds 50a and 51 against the MCF cancer cell line

Compound no.	IC_50_ (µM)
	MCF-7
50a	10.7
51	15.4
DOXO	18.6

## 14. Synthesis of furfuryl-oxadiazole and [(furyl)thiadiazolyl] oxadiazole glycosides

**Scheme 23:**
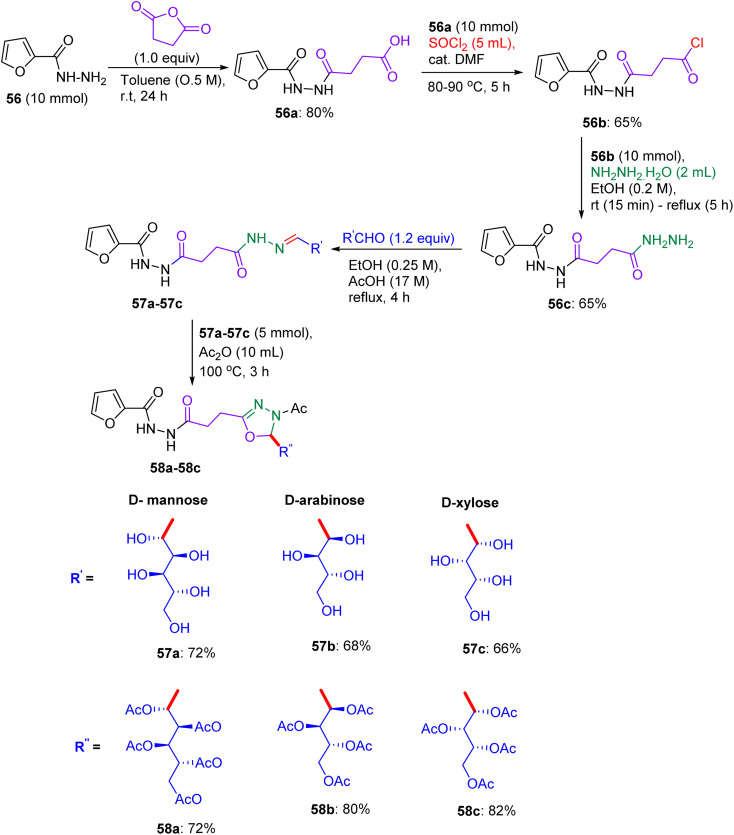
Synthesis of furfuryl-oxadiazole sugar derivatives 58a–58c.

Inspired by innumerable revelations, in 2019, Nassar and El-Sayed presented the systematic design of two series of new furan-assembled 1,3,4-oxadiazole compounds, explicitly for furan-1,3,4-oxadiazole and furan-1,3,4-thiadiazolyl-1,3,4-oxadiazole associated with sugar units.^52^ They utilised furan-2-carbohydrazide 56 as the starting material, which led to the formation of several derivatives as a result of reactions with succinic anhydride, thionyl chloride, and hydrazine hydrate in absolute ethanol to afford acyl hydrazide 56c. An auxiliary heterocycle bonded to furan was afforded by condensation of 56c with monosaccharide aldoses (d-mannose, d-arabinose, and d-xylose) in a catalytic amount of acetic acid, forming sugar hydrazones 57a–57c (Scheme 23) in about 66–72% yields. Finally, reaction with acetic acid anhydride at 100 °C, produced 1,3,4-oxadiazoline-linked acyclic *O*-acetylated-sugar derivatives 58a–58c by heterocyclization.

**Scheme 24:**
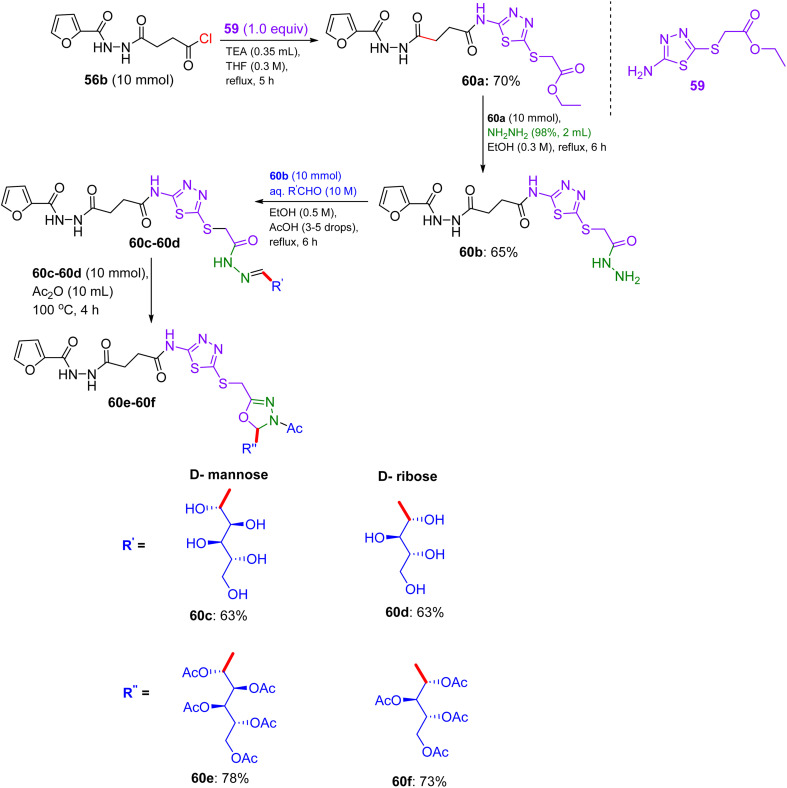
Synthesis of [(furyl)thiadiazolyl] oxadiazole sugar derivatives 60e–60f.

Their designed methodology was applied to introduce an advanced bioactive lead, representing molecular hybridization (insertion of the heterocyclic system into a single molecule). In view of this fact, a five-membered heterocyclic nucleus and 1,3,4-thiadiazole were linked to heterocyclic sugar derivatives owing to the interaction of acid chloride 56b with ethyl 2-((5-amino-1,3,4-thiadiazol-2-yl) thio) acetate 59, generating an ester-functionalized furyl joined with 1,3,4-thiadiazolyl derivative 60a in 70 °C yield, which was then transformed into derived acid hydrazide 60b*via* hydrazinolysis. The intermediate products were characterized by ^1^H-NMR spectroscopy, showing the disappearance of numerous signals and the appearance of new ones. Sugar derivatives were organized with the hydrazide by employing a direct synthetic route, involving the production of sugar hydrazones 60c–60d (Scheme 24). The aforementioned compounds were eventually functionally protected by acetic anhydride treatment at 100 °C to furnish the required sugar furyl-linked 1,3,4-thiadiazole derivatives in excellent yields. The synthesized compounds were analyzed using IR and ^1^H-NMR spectra, which confirmed their established structures.

### 14.1. Findings through lactate dehydrogenase (LDH) assay and binding characteristics of compounds

Compounds 56b, 60a, and 60c, furfuryl-oxadiazole, and [(furyl)thiadiazolyl]-oxadiazole glycosides exhibited remarkable activity against HepG-2 liver cancer cells, with IC_50_ values of 5.5 ± 1.2, 7.29 ± 1.5, and 4.2 ± 1.2 µM, respectively, compared to doxorubicin (IC_50_ ∼ 2–5 µM) (Table 15). Pronounced tumour cell abnormalities underscore their potential as anticancer drugs, with Nassar and El-Sayed highlighting the critical role of the 1,3,4-thiadiazole nucleus.^52^ Docking studies revealed that 56b binds thymidine phosphorylase (TP) or EGFR (*K*_d_ ∼ 2–5 µM) with β-d-glucose, forming H-bonds (for example, His85 and Lys721) and a furfuryl-oxadiazole core, enabling π–π stacking with Phe210 or Tyr residues. Compounds 60a and 60c, which incorporated thiadiazole, showed enhanced binding (*K*_d_ ∼ 1–4 µM), with 60c xylose optimising the H-bonds and thiadiazole boosting the S-mediated interactions. SAR analyses indicated that glycosyl (glucose/xylose) and thiadiazole synergistically enhanced the solubility, GLUT-mediated uptake, and affinity, with 60c substituents minimising steric hindrance. Micro-FTIR confirmed the presence of oxadiazole/thiadiazole (C=N ∼ 1500 cm^−1^ and C–S ∼ 600 cm^−1^) and glycosidic bonds (C–O ∼ 1000–1100 cm^−1^), UFLC-DAD verified their purity, and molecular imaging showed their cytosolic localization. MoA involves TP/EGFR inhibition, reducing angiogenesis/proliferation, and inducing apoptosis *via* ROS, surpassing the DNA-damaging effects of doxorubicin. These binding characteristics position 56b, 60a, and 60c as promising anticancer agents.

**Table 15:** Anticancer activities of 12/7/2025 the oxadiazole sugar derivatives

Compound no.	IC_50_ (µM)	
	HepG-2	RPE-1
56	128.3	55.3
56a	108.5	43.7
56b	5.5	55.4
56c	102.9	68
57a	41.3	128.8
57b	70.6	124.1
57c	31.7	64.9
58a	99.9	62.9
58b	52.1	139.8
58c	115.2	99.5
60a	7.29	63.3
60b	53.7	74.9
60c	4.2	172
60d	30.3	96.8
60f	102.9	90.9
Doxorubicin	3.4	8.9

## 15. Synthesis of diterpenoid isosteviol glycosides

**Scheme 25:**
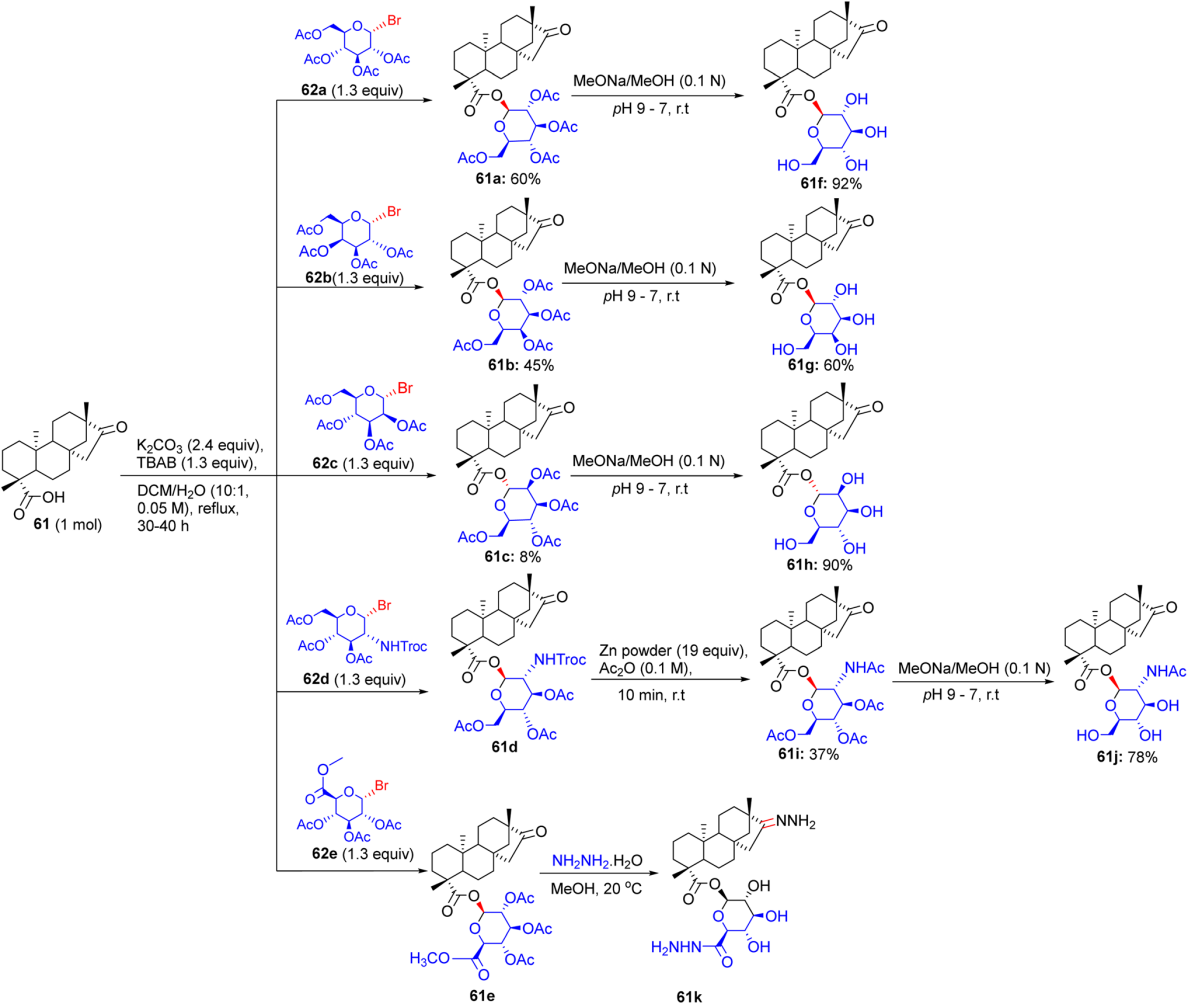
Synthetic routes to isosteviol glycosides 61f–61k.

Almost immediately, an innovative modality was introduced by Sharipova and collaborators in June 2019 for the assembly of glycosides and glycoconjugates of diterpenoid isosteviol.^2^ The synthesis was initiated by reacting the starting material isosteviol 61 with bromo derivatives of monosaccharides 62a–62e in the presence of K_2_CO_3_ and TBAB at 50 °C for 20–48 h, generating glycosides 61a–61e with acetylated hydroxyl groups (Scheme 25). The acetyl protection of glycosides 61a–61c was removed by sodium methoxide at 20 °C for 2 h, producing novel glycosides 61f–61h. The trichloroethylformate deprotection of the amino group of glycoside 61d was performed using powdered Zn in acetic anhydride at room temperature to afford glycoside 61i. The deacylation of 61i was done with sodium methoxide in methanol producing glycoside 61j. The deacetylation of glycoside 61e was completed in the presence of hydrazine hydrate in methanol to afford pharmacophoric hydrazide and hydrazone moieties incorporated glycoside 61kh.

**Scheme 26:**
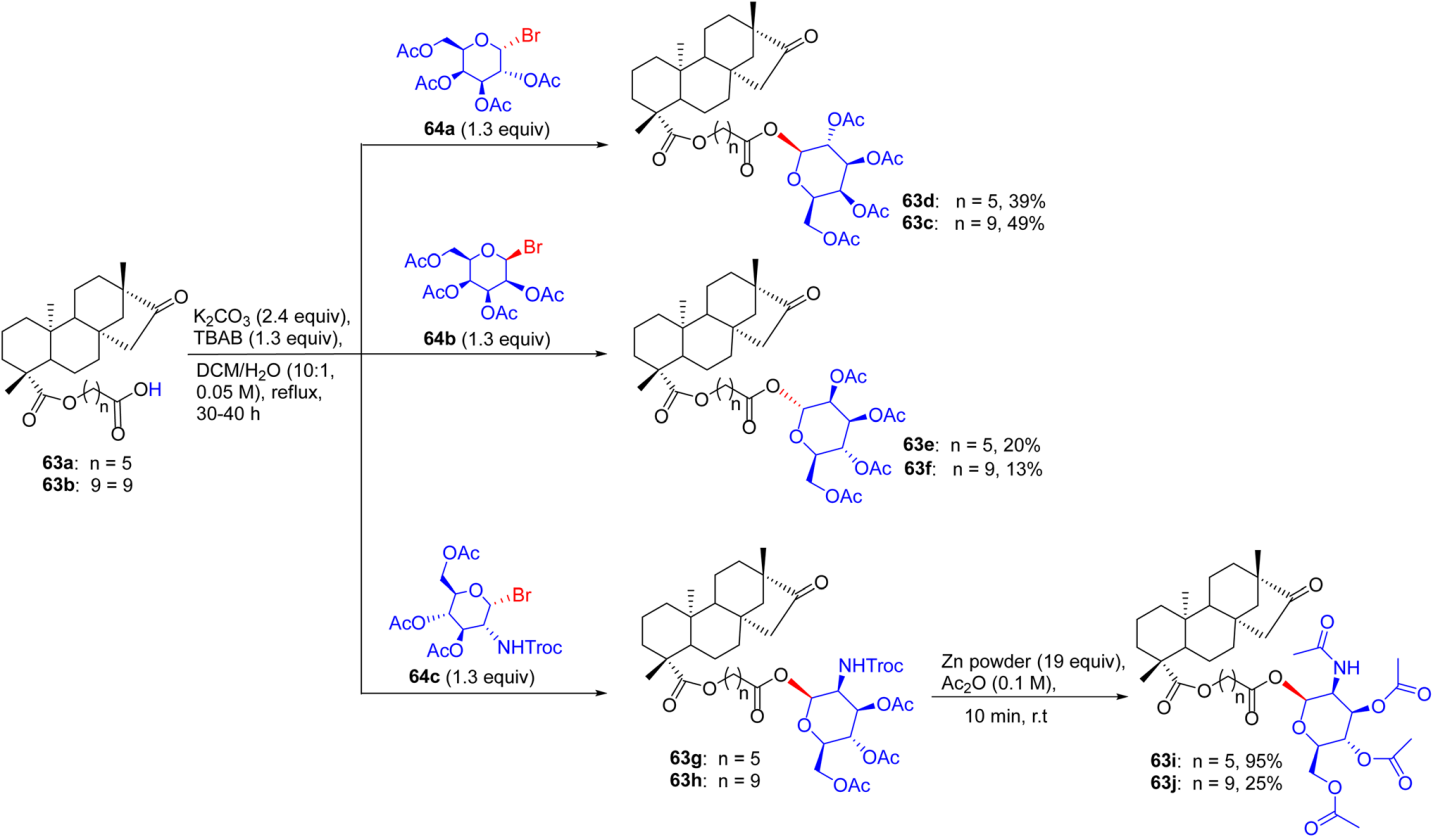
Synthesis of isosteviol glycoconjugates (63d–63j) having monosaccharides distant from the diterpenoid skeleton.

Futhermore, the isosteviol glycoconjugates were prepared as starting materials, having a carbohydrate residue distant from the diterpenoid skeleton *via* a polymethylene linker. Then, these isosteviol derivatives 63a and 63b were subjected to reaction with bromo derivatives of monosaccharides 64a–64c for 20–48 h in the presence of K_2_CO_3_ and TBAB at 50 °C, supplying glycoconjugates 63c–63h with acetylated hydroxyl groups (Scheme 26). The amine groups in glycoconjugates 63g and 63h were deprotected by Zn powder in acetic anhydride at room temperature to furnish glycoconjugates 63i and 63j. The glycosidic bond may be hydrolyzed under the conditions of various deprotection modes for hydroxyl groups in glycoconjugates 63c–63f, 63i, and 63j. The ^1^H NMR spectra of the products indicated the presence of anomeric protons for some glycosides and glycoconjugates, resonating as a doublet; this substantiates the configuration of the β-anomer, whilst the other analyses confirm the α-orientation of the glycosidic bonds.

### 15.1. Findings through *in vitro* cytotoxicity and binding characteristics of compounds

Diterpenoid isosteviol glycosides exhibited tremendous cytotoxic activity against M-HeLa cells, with lead compounds 61c, 61e, 63c, 63e, 71c, and 71e having IC_50_ values of ∼1–3 µM, which are three-fold more selective than tamoxifen (IC_50_ ∼ 10–20 µM), and non-toxic to Chang liver cells (IC_50_ > 40 µM) (Table 16). Docking studies revealed that 61c, with tri-*O*-acetylated d-mannopyranosyl directly linked to the isosteviol backbone, binds Bax or Bcl-2 (*K*_d_ ∼ 1–4 µM), with acetylated mannose forming H-bonds (for example, Ser184 and Asp102), and an isosteviol core that enables hydrophobic interactions with Phe.^2^ Compounds 61e, 63c, 63e, 71c, and 71e exhibited similar Bax/Bcl-2 binding, with variations in their glycosylation (glucose/xylose) and acetylation modulating affinity. SAR analyses highlighted that the proximity of acetylated glycosyl to the isosteviol skeleton enhances the lipophilicity and GLUT-mediated uptake, with the direct mannose linkage of 63c optimizing its potency. Micro-FTIR confirmed the presence of glycosidic (C–O ∼ 1000–1100 cm^−1^) and acetyl (C=O ∼ 1700 cm^−1^) bonds, UFLC-DAD verified their purity, and molecular imaging showed their mitochondrial localization. MoA is involved in mitochondrial apoptosis *via* Bax activation, reducing the mitochondrial membrane potential (Δ*ψ*_m_) and caspase activation, with 65c and 63e inducing early apoptosis. Compounds 61c, 71c, and 71e reduced the Δ*ψ*_m_ in M-HeLa cells, confirming their mitochondrial pathway activation. Haemolytic assays showed that 61c and 63c caused no erythrocyte rupture at 100 µM, whereas 63e exhibited minor haemolysis (∼6% at 100 µM), indicating their low toxicity. These binding characteristics and structural features indicate that 61c, 61e, 63c, 63e, 71c, and 71e are promising anticancer agents.

**Table 16:** Cytotoxicity of the glycosides and glycoconjugates of diterpenoid isosteviol

Compound no.	IC_50_ (µM)		
	M-HeLa	MCF-7	Chang liver
61a	>100	>100	>100
61b	>100	>100	>100
61c	15.1	29.3	>100
61e	26	65	>100
61f	32	>100	>100
61g	52	29	58
61h	>100	>100	>100
61i	18	36	86
61j	>100	>100	>100
61k	>100	>100	>100
63c	10	32.1	>100
63d	27	33.2	>100
63e	11	63.2	>100
63f	18	31	>100
63i	46	>100	46
63j	>100	64.9	>100
Tamoxifen	28	25	46

## 16. Synthesis of pyrazoline and isoxazole-linked indole *C*-glycosides

**Scheme 27:**
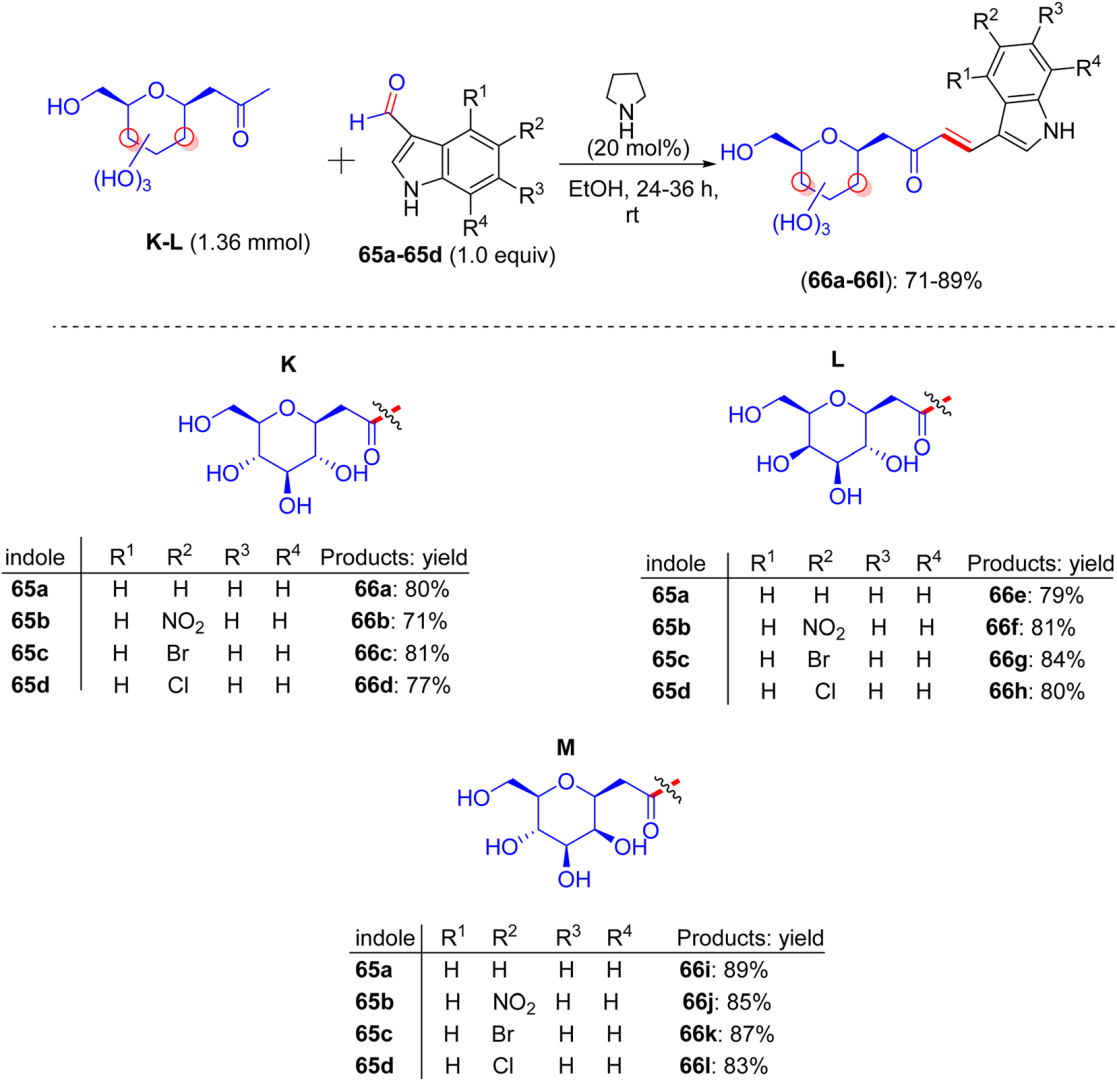
Synthesis of *C*-glycosides of diverse indoles 66a–66l.

Inventions and discoveries in this field of carbohydrate research are constantly in progress. In 2020, Sagar *et al.* presented a modified method for the preparation of pyrazoline- and isoxazole-linked indole *C*-glycoside hybrids (Scheme 27).^53^ In the first stage, *C*-glycosides 66a–66l were used to form α,β-unsaturated-*C*-β glycosidic ketone fragments, and further condensed with hydrazine hydrate or hydroxyl amines at room temperature to higher temperatures, transforming them into pyrazoline or isoxazole-bridged *C*-glycoside of indole. Among the vast list of carbohydrates, d-glucose, d-galactose, and d-mannose (K–L) were employed to react with indole 3-carboxaldehydes 65a–65d, creating α,β-unsaturated ketone *C*-glycosides 66a–66l*via* pyrrolidine-catalyzed aldol condensation in 71–89% yields after 37 h.

**Scheme 28:**
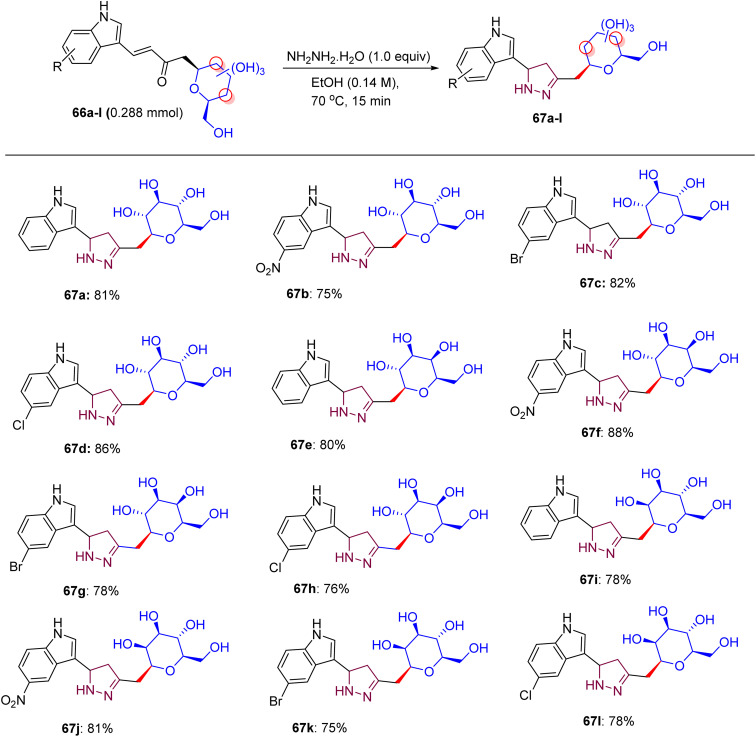
Structures of pyrazoline-linked indole *C*-glycopyranosides 67a–67l.

**Scheme 29:**
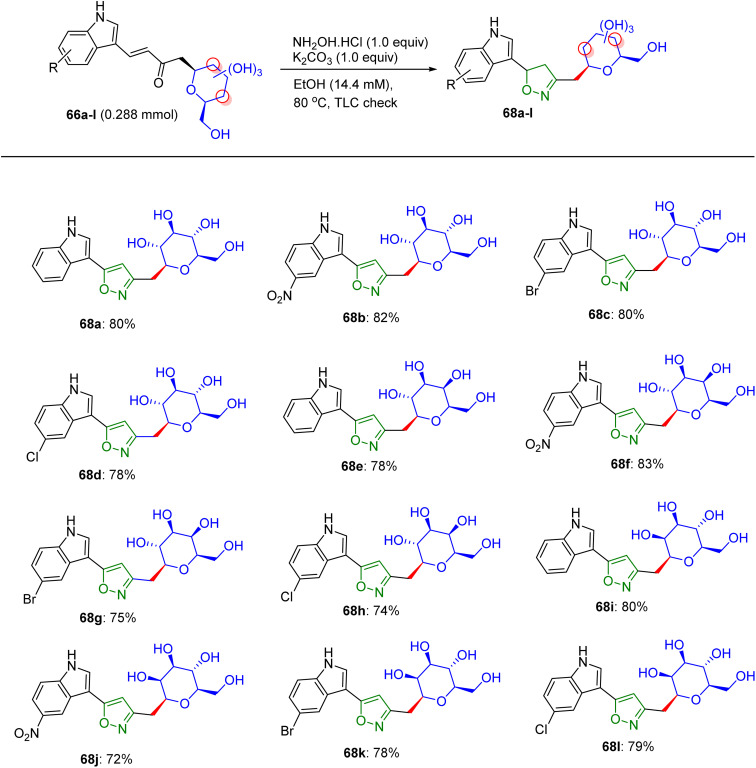
Structures of isoxazole-linked indole *C*-glycopyranosides 68a–68l.

These compounds tend to react with 1,2-dinucleophiles, most likely with hydrazine hydrate and hydroxylamine hydrochloride, and give pyrazole/pyrazoline or isoxazole-linked *C*-glycosides of indole. Subsequently, the initial product *gluco*-linked α,β-unsaturated-*C*-β glycosidic ketone molecule 66a was reacted with hydrazine hydrate at an average temperature of 61 °C, to give pyrazoline (Scheme 28). An analogous reaction can be conducted under microwave conditions to synthesize compound 67a in 81% yield. Numerous *gluco*-, *galacto*-, and manno-derived α,β-unsaturated-*C*-β glycosidic ketone molecules 66a–66l were converted to pyrazoline-linked indole *C*-glycopyranosides 67a–67l upon microwave treatment within ethanoic solvent at 70 °C, respectively. Employing the trial and error method, it was found that *gluco*-linked α,β-unsaturated-*C*-β glycosidic ketone compound 65i and hydroxyl amine hydrochloride in the presence of ethanol under ambient temperature do not produce any detectable quantity of product. Conversely, the equivalent reaction in the presence of K_2_CO_3_ at 60 °C produces isoxazole 68a in 80% yield. Finally, α,β-unsaturated-*C*-β glycosidic ketone molecules 66a–66l were treated with the hydroxyl amine hydrochloride by means of K_2_CO_3_ at 60 °C, and this reaction was accomplished in 2 h, delivering *gluco*-, *galacto*-, and manno-linked isoxazole-bridged *C*-glycosides of indoles 68a–68l in excellent yields (Scheme 29). The compounds were well-characterized by NMR and mass spectrometry, which revealed distinct isoxazole and pyrazoline proton and carbon peaks in the final products.

### 16.1. Findings through MTT assay and binding characteristics of compounds

The activities of α,β-unsaturated *C*-glycosides of indoles (66a–66l), pyrazoline-linked *C*-glycosides (67a–67l), and isoxazole-linked *C*-glycosides (68a–68l) were delineated in MDA-MB-231 cells using the MTT assay (Tables 17–19), respectively. Compounds 66c, 67f, 67g, 68a, 68c, 68d, 68f, 68g, 68j, and 68k showed moderate growth inhibition at 10 µM (IC_50_ ∼ 5–10 µM), whereas 66a, 66f, 66l, 67e, and 68b inhibited cell growth by approximately 50% at 25 µM (IC_50_ ∼ 10–15 µM), with higher potency in MCF-7 cells (IC_50_ ∼ 5–10 µM) and no activity in normal breast epithelial cells (IC_50_ > 40 µM).^53^ Docking studies revealed that pyrazoline-linked 67f and 67g bind EGFR or tubulin (*K*_d_ ∼ 2–5 µM) with β-d-glucose/xylose to form H-bonds (for example, Lys721 and Ser178) and an indole-pyrazoline core, enabling π–π stacking with Phe699 or Phe255, respectively. Isoxazole-linked 68a, 68c, 68d, 68f, 68g, 68j, and 68k showed enhanced binding (*K*_d_ ∼ 1–4 µM) due to the O–N linkage of isoxazole, with 68b showing weaker binding due to its suboptimal substituents. SAR analyses highlighted the *C*-glycoside synergy with pyrazoline/isoxazole, which enhanced the solubility, GLUT-mediated uptake, and affinity. Aryl substituents on pyrazoline/isoxazole (*e.g.*, 67f/g and 68f/g) boosted π–π stacking, respectively. Micro-FTIR confirmed the presence of *C*-glycosidic (C–C ∼ 1100 cm^−1^), pyrazoline (C=N ∼ 1500 cm^−1^), and isoxazole (C=N ∼ 1600 cm^−1^) bonds, UFLC-DAD verified their purity, and molecular imaging showed their cytosolic localization. MOA involves EGFR inhibition, reduced proliferation, or tubulin disruption, inducing G2/M arrest and apoptosis, and is selective for MCF-7 cells over normal cells, outperforming the ER-mediated effects of tamoxifen.

**Table 17:** Cell viability results at different concentrations of the given compounds

Compound no.	Cell viability (%)		
	50 µM	25 µM	10 µM
66a	36.32	54.03	71.14
66b	85.37	84.49	91.4
66c	107.54	100.13	100.25
66d	74.75	89.8	79.24
66e	84.22	94.24	97.58
66f	60.56	58.98	73.61
66g	67.46	113.07	125.13
66h	56.7	89.59	75.04
66i	79.43	80.48	84.1
66j	69.81	78.47	71.68
66k	71.48	89.2	102.51
66l	61.98	54.28	56.7
67a	75.04	76.76	73.46
67b	70.33	68.62	70.4
67c	81.53	88.82	104.77
67d	77.74	77.51	82.23
67e	43.82	54.17	97.58
67f	99.5	98.25	100.58
67g	97.74	90.81	90.05
67h	63.36	95.16	99.5
67i	73.55	76.56	82.22
67j	87.98	92.06	93.84
67k	87.57	90.13	91.41
67l	66.54	78.49	74.63
68a	92.63	106.38	103.51
68b	43.4	46.1	68.37
68c	93.89	95.31	95.45
68d	99.33	96.8	97.84
68e	64.41	76.39	74.75
68f	94.9	94.45	99.1
68g	90.31	89.03	120.42
68h	87.93	96.03	93.7
68i	73.96	81.22	89.23
68j	95.23	93.37	92.62
68k	95.7	85.24	96.59
68l	81.68	82.94	83.9
YM155 (20 nM)	27.39	—	—
Menadione (20 µM)	20.77	—	—

**Table 18:** Cell viability outcomes at different concentrations on breast cancer cell lines

Compound no.	Cell viability (%)								
	MCF-7			MDA-MB-453			MCF-10A		
	25 µM	10 µM	1 µM	25 µM	10 µM	1 µM	25 µM	50 µM	100 µM
66a	53	62	79	96	76	80.6	87	81	84
66f	66	69	100	65	82	80.6	86	84	80
66l	47	58	79.2	66	74	105	85	78	36
67e	19	34	66	69	78	88.8	87	84	82
68b	23	28	41.3	66	80	92.5	87	80	84
68e	19	21	30.8	96	76	95.4	86	83	81
YM155	37	37	37.2	33	33	33	48	48	48

**Table 19:** Cytotoxicity of the pyrazoline- and isoxazole-linked indole *C*-glycoside hybrids^a^

Compound no.	IC_50_ (µM)	
	MCF-7	MDA-MB-231
66a	30	30.6
66l	20.99	ND
67e	4.67	35.5
68b	0.71	22.3
68e	0.67	ND

a ND = not determined.

## 17. Synthesis of thiazole and imidazothiazole *C*-glycosides

**Scheme 30:**
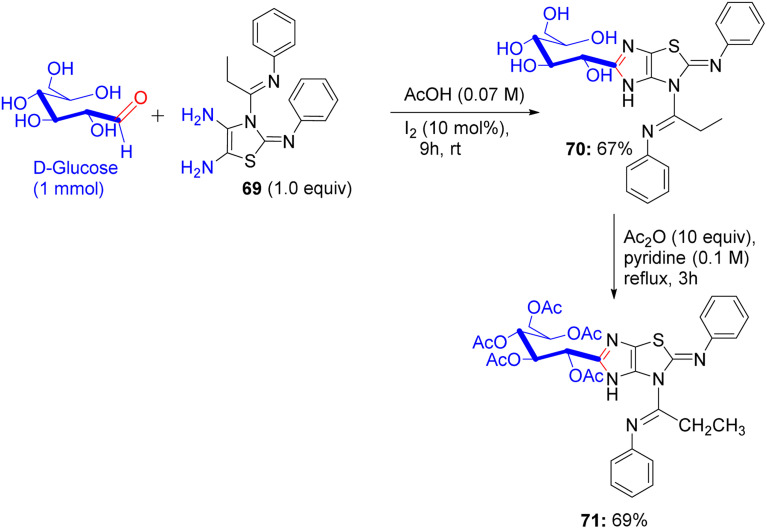
Synthesis of compounds 70 and 71.

About a half year later, in 2020, Ghoneim shed light on the synthesis of acyclic *C*-glycosides. The starting amide was reacted with thionyl chloride at room temperature for 2 h to form the imidoyl chloride derivative (Scheme 30).^54^ Then, the reaction of imidoyl chloride with potassium isothiocyanate and subsequent reaction with aniline afforded thiourea derivatives, which on refluxing with 2,3-diaminomaleonitrile for 10 h underwent cyclization, delivering thiazole derivatives 69. In addition, compound 69 was treated with d-glucose and iodine in acetic acid at room temperature, forming *C*-glycoside imidazo[4,5-*d*]thiazole derivative 70, which was protected with acetic anhydride and pyridine under reflux conditions to provide protected glycosides 71.

**Scheme 31:**
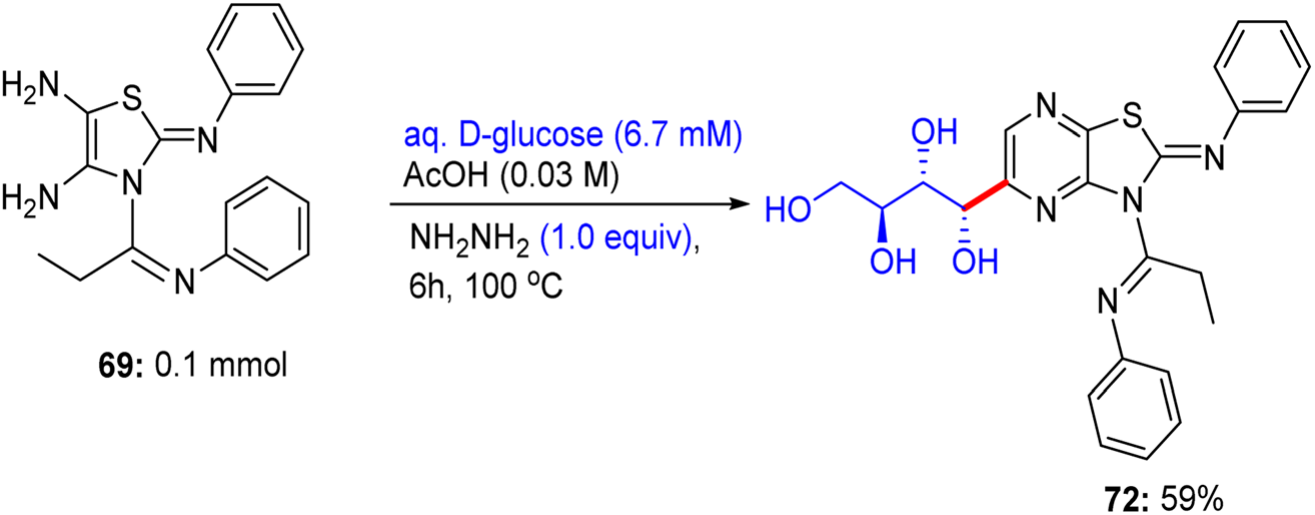
Synthesis of compound 72.

**Scheme 32:**
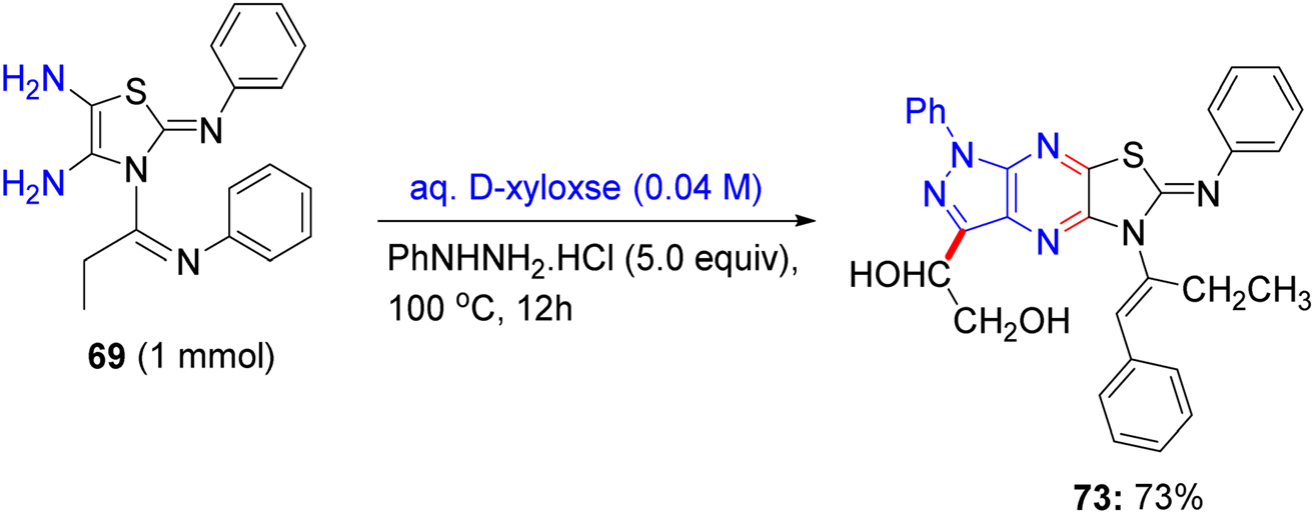
Synthesis of compound 73.

In the following step, the reaction of thiazole-4,5-diamine derivatives 69 with hydrazine hydrate and d-glucose in acetic acid at 100 °C for 6 h afforded acyclic analogs *C*-glycoside derivatives 72 (Scheme 31). Finally, compound thiazole-4,5-diamine derivatives 69 condensed with d-xylose and phenylhydrazine hydrochloride in acidic medium at 100 °C, forming the glucose phenylhydrazone intermediate, which can be converted to acyclic analog *C*-glycoside derivatives 73 (Scheme 32).

### 17.1. Findings through *in vitro* cytotoxicity and binding characteristics of compounds

Bioanalysis revealed that thiazole and imidazo-thiazole *C*-glycosides 70, 71, 72, and 73 exhibited lower IC_50_ values (∼1–3 µM) in HCT-116 cells, with 72 and 73 showing superior potency in PC-3 (∼2–4 µM) and 70 and 72 demonstrating moderate activity in HepG-2 (∼5–8 µM) compared to doxorubicin (IC_50_ ∼ 2–5 µM) (Table 20).^54^ Docking studies indicated that 70 binds to EGFR or topoisomerase II (Topo II, *K*_d_ ∼ 2–5 µM) with its β-d-glucose forming H-bonds (for example, Lys721 and Arg503) and the thiazole core enabling π–π stacking with Phe699 or Phe1122. Compounds 72 and 73, with imidazo-thiazole cores, showed enhanced binding (*K*_d_ ∼ 1–4 µM) due to the rigidity of the fused imidazole ring, with 78 aryl substituents optimising the H-bonds and hydrophobic interactions in PC-3. Compound 71 binds less effectively (*K*_d_ ∼ 3–6 µM) because of its simpler substituents. SAR analyses highlighted the *C*-glycoside synergy with thiazole/imidazo-thiazole, enhancing the solubility, GLUT-mediated uptake, and affinity, with the aryl groups of 72 boosting its potency. Micro-FTIR confirmed the presence of *C*-glycosidic (C–C ∼ 1100 cm^−1^), thiazole (C=N ∼ 1500 cm^−1^ and C–S ∼ 600 cm^−1^), and imidazo-thiazole bonds, UFLC-DAD verified their purity, and molecular imaging showed their cytosolic/nuclear localisation. MoA involves EGFR inhibition, reducing proliferation, or Topo II inhibition, stabilising DNA breaks, leading to apoptosis, with 72 and 73 excelling in HCT-116 and PC-3 cells, respectively. These binding characteristics suggest that 70, 71, 72, and 73 are promising anticancer agents.

**Table 20:** Cytotoxicity of the acyclic *C*-glycosidic compounds

Compound no.	IC_50_ (µM)		
	HCT-116	PC-3	HepG-2
69	175.1	182.1	507.3
70	119	2229	133.5
71	94.8	280.7	284.1
72	91.2	119.8	175.9
73	106.2	111.5	333.9
Doxorubicin	126.8	129.6	116.9

## 18. Synthesis of triazolo-pyrimidine glycosides

**Scheme 33:**
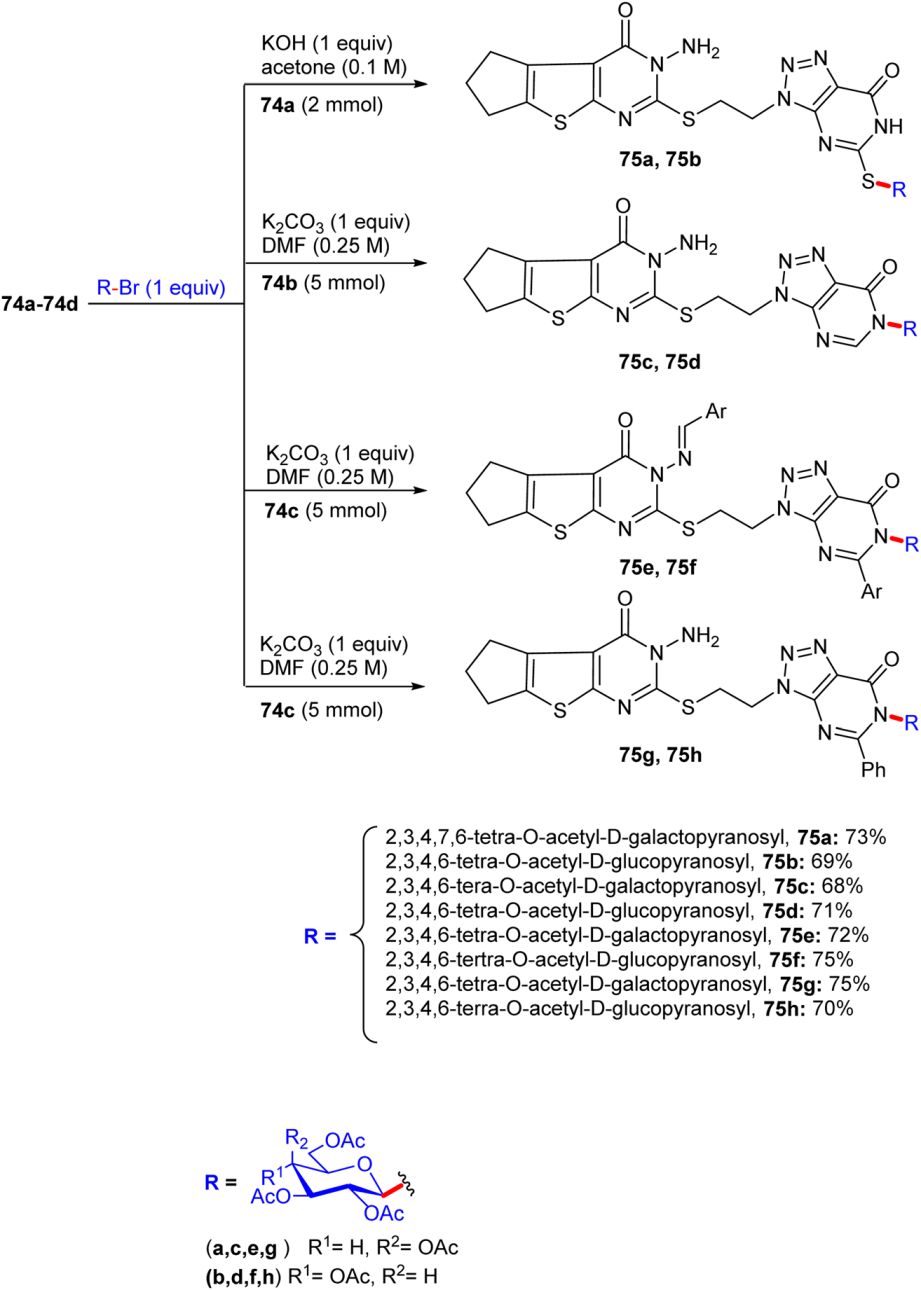
Synthesis of triazolopyrimidine glycoside derivatives.

**Scheme 34:**
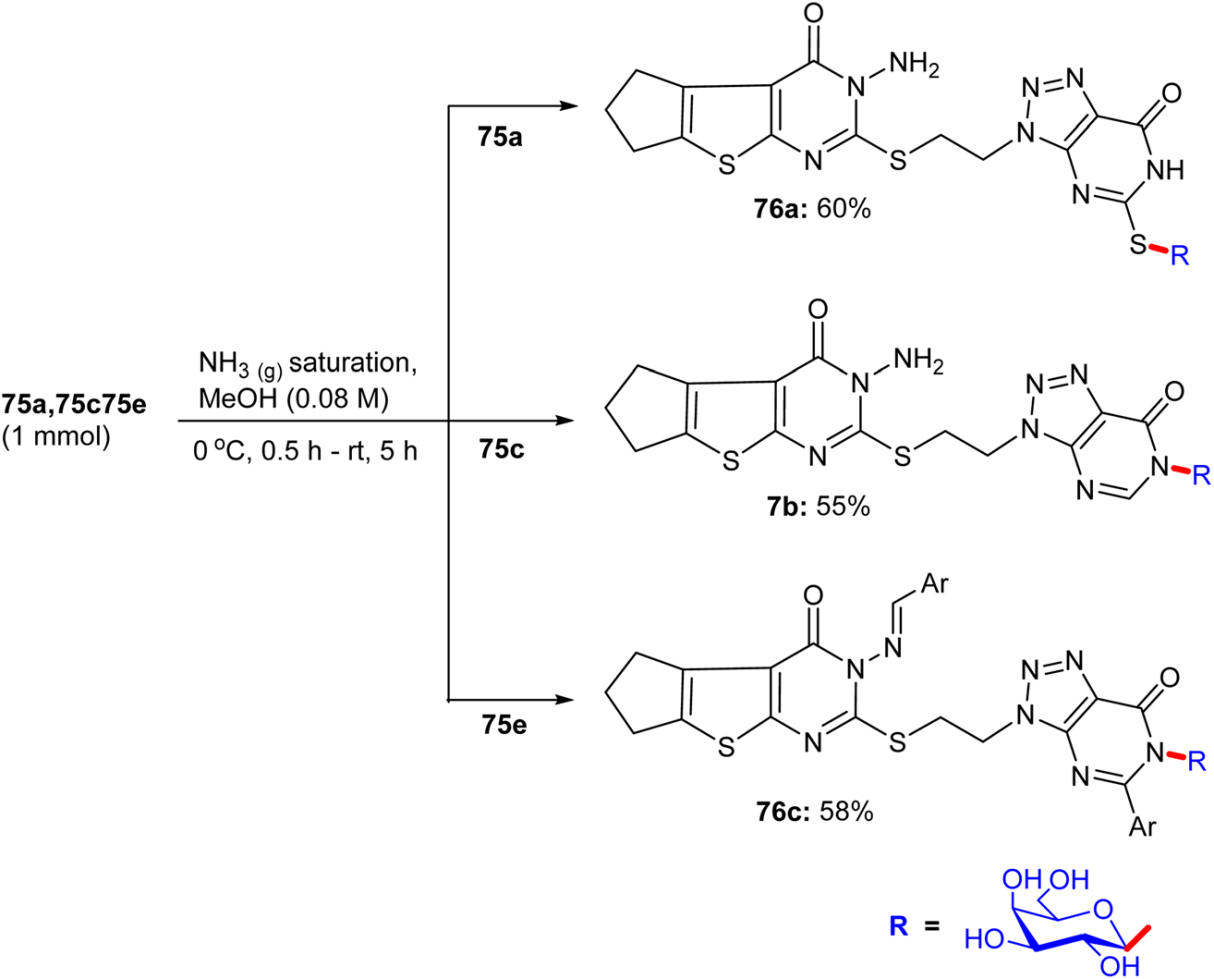
Synthesis of deacetylated thienopyrimidine glycoside derivatives.

In 2021, Hassan and El-Sayed reported the synthesis of triazolo[4,5-*d*]pyrimidine derivatives connected to a thienopyrimidine ring (Schemes 33 and 34).^55^ Triazolopyrimidine products 74a–74d were treated with various per-*O*-acetylated *galacto*- and *gluco*-pyranosyl bromide in K_2_CO_3_/DMF to synthesize thioglycosides 75a and 75b, and their *N*-glycoside analogues (75c and 75d), (75e and 75f), (75g and 75h), respectively (Scheme 33). Finally, per-*O*-acetylated glycosides 75a, 75c, and 75e were transformed into free hydroxyl group glycosides 76a, 76b, and 76c, respectively, using dry methanol saturated with gaseous ammonia at room temperature.

### 18.1. Findings through cytotoxicity analyses and binding characteristics of compounds

Compounds tested on HepG-2 and MCF-7 cancer cell lines exhibited concentration-dependent antitumour activity, with triazolopyrimidine glycosides incorporating p-chlorophenyl groups or tetra-*O*-acetyl glycopyranosyl showing enhanced cytotoxicity (IC_50_ ∼ 1–3 µM in HepG-2 and ∼2–4 µM in MCF-7) compared to doxorubicin (IC_50_ ∼ 2–5 µM) (Table 21).^55^ Docking studies revealed that tetra-*O*-acetyl glycopyranosyl derivatives bind EGFR or thymidylate synthase (TS, *K*_d_ ∼ 1–4 µM) with β-d-glucose/mannose to form H-bonds (for example, Lys721 and His192), and the *p*-chlorophenyl group enhances π–π stacking with Phe699 or Phe225. Deprotected thioglycosides, with sulfur-mediated H-bonds, show higher potency in MCF-7 cells (IC_50_ ∼ 1–3 µM) but reduced efficacy in HepG-2 cells (∼5–8 µM) due to lower lipophilicity. SAR analyses revealed that tetra-*O*-acetyl groups boosted the lipophilicity and GLUT-mediated uptake in HepG-2 cells, while p-chlorophenyl and thioglycoside sulfur enhanced the MCF-7 activity through π–π stacking and polar interactions. Micro-FTIR confirmed the presence of glycosidic (C–O ∼ 1000–1100 cm^−1^), acetyl (C=O ∼ 1700 cm^−1^), thioglycosidic (C–S ∼ 600 cm^−1^), and triazolo-pyrimidine (C=N ∼ 1500 cm^−1^) bonds, UFLC-DAD verified the purity, and molecular imaging showed the cytosolic localization. The MoA involves EGFR inhibition, reducing proliferation, or TS inhibition, blocking DNA synthesis, and leading to apoptosis, with acetylated glycosides excelling in HepG-2 and thioglycosides in MCF-7, complementing Na^+^/K^+^-ATPase and gallic acid glycoconjugate tubulin targeting of cardiac glycosides.^39^

**Table 21:** Cytotoxicity of *N*-glycoside- and thioglycoside-derivatives of triazolo[4,5-*d*]pyrimidine-based thienopyrimidine compounds

Compound no.	IC_50_ (µM)	
	HepG-2	MCF-7
75a	26.7	11.4
75c	28.6	9.1
75e	27.2	9.8
75b	27.8	10.2
75d	26.7	9.3
75f	26.7	8.6
75g	32.7	8.3
75h	29.9	8.6
76a	33	8.1
76b	30.9	10.4
76c	29.8	10.2
Doxorubicin	28.5	10.3

## 19. Synthesis of aminoglycosides

**Scheme 35:**
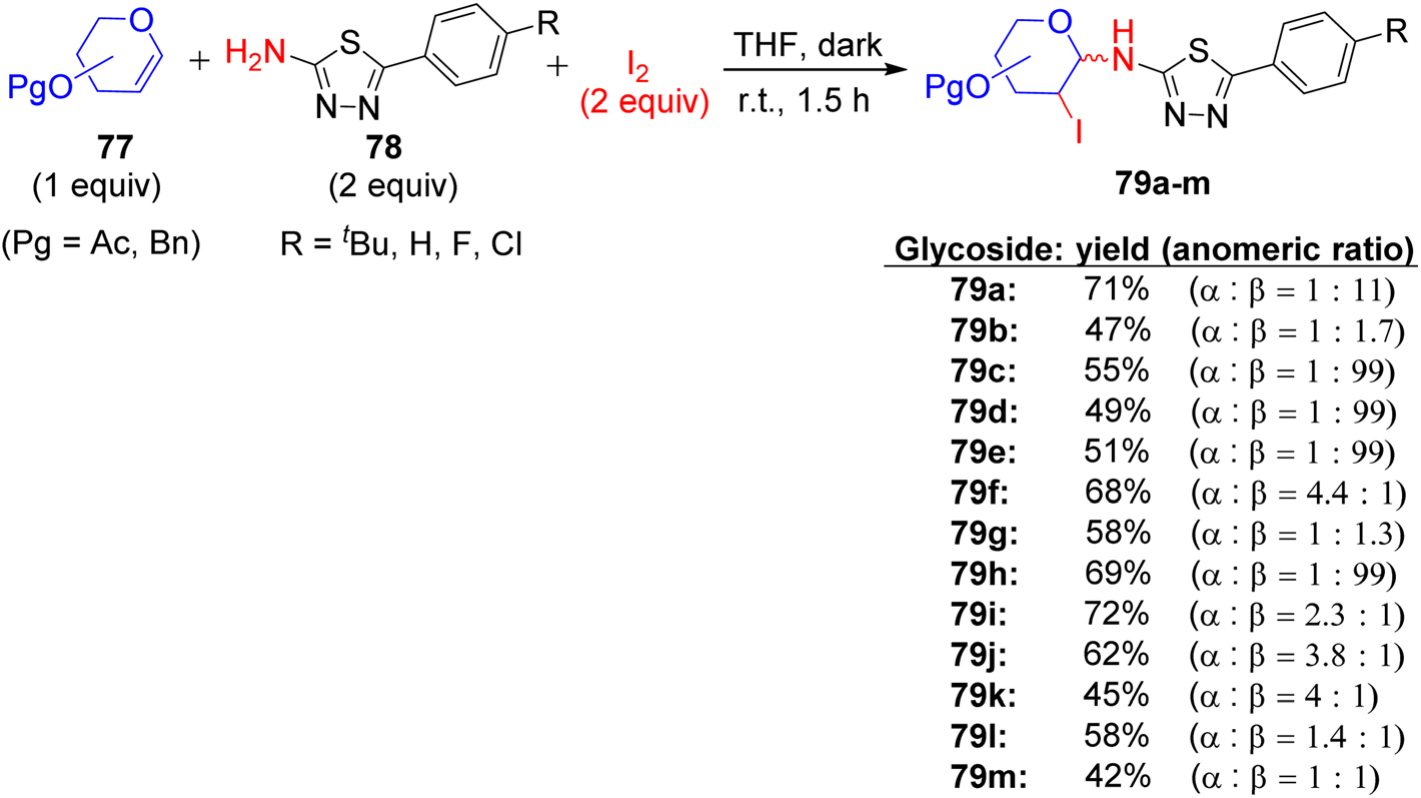
Synthesis of aminoglycosides.

Kasprzycka and co-workers in November 2021 synthesized aminoglycosides by reacting glycals with 2-amino-1,3,4-thiadiazole derivatives and extended this idea.^56^ For this purpose, thiadiazole derivatives 78 were treated with unsaturated sugars 77 with the aid of iodine in the dark at ambient temperature, yielding conjugates 79a–m (Scheme 35). Then, column chromatography was used to separate these products, and their structures were analyzed by spectroscopic methods.

### 19.1. Findings through MTT assay and binding characteristics of compounds

The effects of aminoglycosides 79 on MCF-7, HCT116, and HeLa cancer cell lines were assessed *via* the MTT assay, revealing higher potency in HCT116 (IC_50_ ∼ 1–3 µM) than MCF-7 (∼3–5 µM), with a moderate survival fraction (SF) reduction in HeLa cells (∼2–4 µM).^56^ Flow cytometry confirmed G2/M arrest in HCT116 and HeLa cells, while annexin V/PI assays indicated pro-apoptotic effects, most pronounced in HCT116 cells. Docking studies showed that 79a (*e.g.* neomycin based) binds to ribosomal RNA (16S A-site, *K*_d_ ∼ 1–3 µM) or topoisomerase II (Topo II, *K*_d_ ∼ 2–4 µM) with amino sugars (*e.g.* glucosamine), forming hydrogen bonds (for example, A1492 and Arg503) and modified groups (*e.g.* acetyl and aryl), enabling π–π stacking with RNA bases or Phe1122. Acetylated variants (*e.g.*79b) enhanced the lipophilicity and improved the potency against HCT116 cells. SAR analyses highlighted amino-sugar multiplicity and acetylation as key for rRNA/Topo II affinity, with aryl modifications boosting the hydrophobic interactions in HCT116 cells. Micro-FTIR confirmed the presence of amino-sugar (C–N ∼ 1200 cm^−1^, O–H ∼ 3300 cm^−1^) and acetyl (C=O ∼ 1700 cm^−1^) bonds, UFLC-DAD verified their purity, and molecular imaging showed their cytosolic/nuclear localization. MoA involves rRNA binding, causing mistranslation or Topo II stabilisation, inducing DNA breaks, leading to G2/M arrest and apoptosis, with cytostatic effects in HeLa cells and cytotoxic/lethal effects in HCT116 cells. These binding characteristics position the 79 series as an representative for further modification.

## 20. Synthesis of 1,2,3-triazole-coumarin-glycoside and 1,2,4-triazolyl thioglycoside

**Scheme 36:**
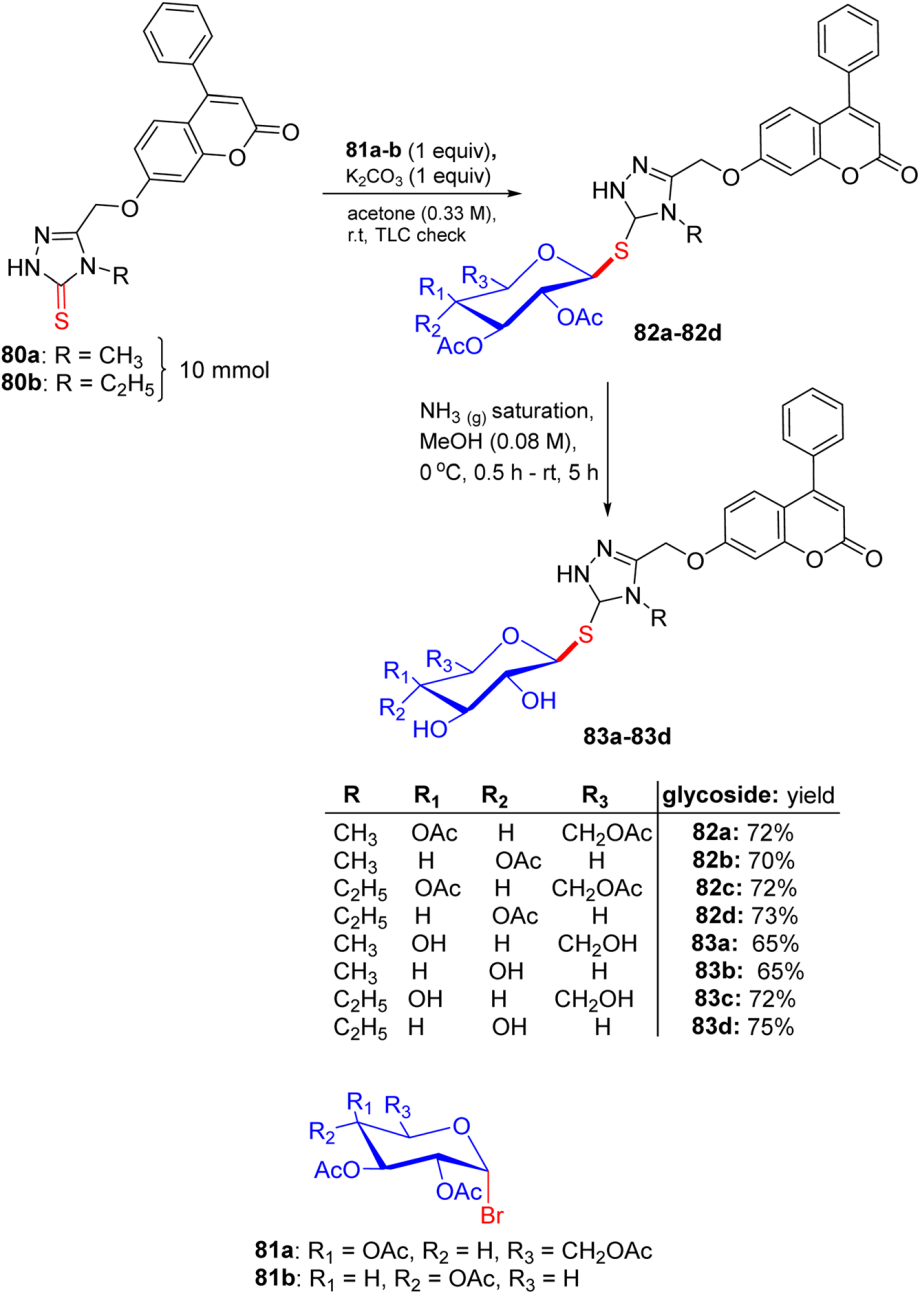
Synthesis pathway to 1,2,4-triazole-coumarin hybrid thioglycosides.

In 2022, Alminderej and Kassem proposed the synthesis of an azolyl system linked to coumarin, and performed glycosylation reactions on *N*^1^-alkylated coumarin-1,2,4-triazole derivatives 80a–b by means of acetylated bromogalactopyranosyl or xylopyranosyl (81a–b), yielding 1,2,4-triazole thioglycosides 81a–d).^57^ Subsequently, deacylation in methanolic ammonia afforded hydroxyl-deprotected thioglycosides 83a–83d (Scheme 36).

**Scheme 37:**
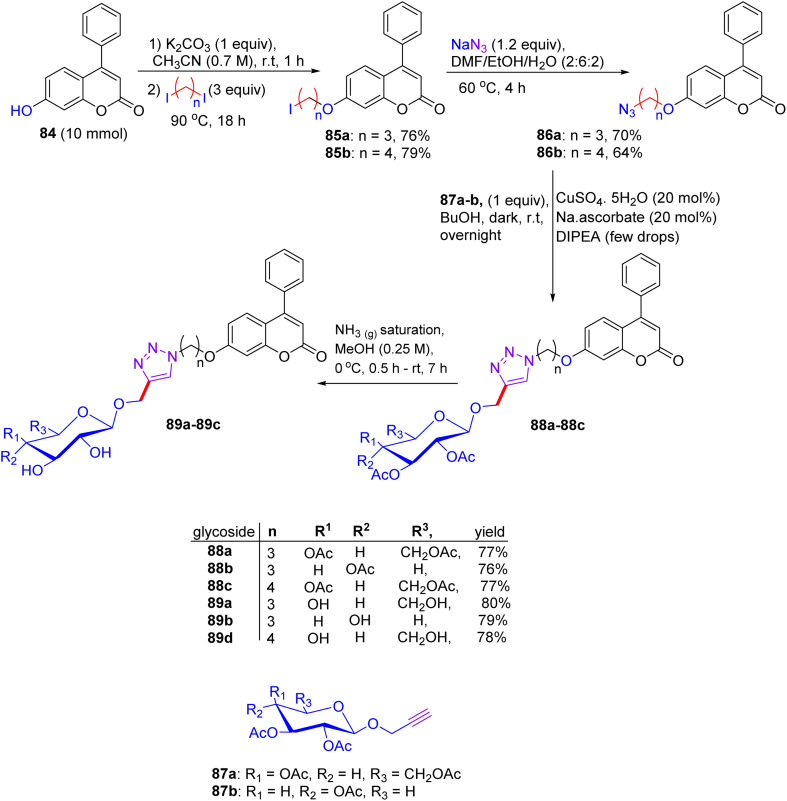
Proposed pathway to 1,2,3-triazol-coumarin hybrid glycosides.

At this point, CuCAA reaction combined the 1,2,3-triazole-*C*-glycosides with the coumarin molecule by click dipolar cycloaddition (Scheme 37). In the next step, alkylation of 7-hydroxycoumarin 84 with diiodopropane and diiodobutane delivered compounds 85a and 85b, respectively. These iodo-derivatives were converted into azide products 86a and 86b using sodium azide. Then, the *C*-glycosides 88a–88c were obtained by reacting azide 86a and b with terminal acetylenic sugars 87a and b under click conditions with copper sulfate and sodium ascorbate. Ultimately, deacetylation of 1,2,3-triazole-*C*-glycosides 88a–88c in methanolic ammonia afforded hydroxyl-containing glycosides 89a–89c.

### 20.1. Findings through MTT assay and binding characteristics of compounds

1,2,3-Triazole-coumarin-glycosides (83b, 83c, 84, and 85a) and 1,2,4-triazolyl thioglycoside (88) were evaluated for their potency against the HOS, MDA-MB-231, MCF-7, Caco-2, and HCT-116 cell lines using the MTT assay, demonstrating their significant anticancer activity (IC_50_ ∼ 1–3 µM in MCF-7 and HCT-116; ∼2–5 µM in other cell lines) (Table 22).^57^ Coumarin-triazole 84 exhibited superior activity in MCF-7 cells by modulating the expression of apoptotic proteins (cytochrome c, caspase-7, Bcl-2, and Bax). Docking studies revealed that 84 binds to Bcl-2 or EGFR (*K*_d_ ∼ 1–3 µM), with acetylated β-d-glucose forming hydrogen bonds (for example, Asp108 and Lys721), and the coumarin-triazole core facilitates π–π stacking with Phe104 or Phe699. Compounds 83b, 83c, and 85a showed slightly weaker binding (*K*_d_ ∼ 2–4 µM) due to substituent variations (aryl *vs.* alkyl), while the thioglycoside of 88 binds similarly (*K*_d_ ∼ 2–4 µM) to sulfur-mediated H-bonds. SAR analyses highlighted the lipophilicity of acetylated glycosides and rigidity of 1,2,3-triazole for MCF-7 potency, with aryl substituents (*e.g.*83b and 84) enhancing π–π stacking. 1,2,4-Triazolyl thioglycoside 88 benefits from sulfur interactions but is less potent due to reduced lipophilicity. Micro-FTIR confirmed the presence of glycosidic (C–O ∼ 1000–1100 cm^−1^), acetyl (C=O ∼ 1700 cm^−1^), thioglycosidic (C–S ∼ 600 cm^−1^), and triazole/coumarin (C=N ∼ 1500 cm^−1^) bonds, UFLC-DAD verified their purity, and molecular imaging showed their mitochondrial/cytosolic localisation. MoA involves Bcl-2 inhibition, promoting cytochrome c release and caspase-7 activation, or EGFR inhibition, which reduces proliferation and leads to apoptosis, with 90% efficacy in MCF-7 cells. These binding characteristics position 83b, 83c, 84, 85a, and 88 as promising for further modification and evaluation study.

**Table 22:** Cytotoxicity results of 1,2,3-triazole-coumarin-glycoside and 1,2,4-triazolyl thioglycoside across various cell lines

Compound no.	IC_50_ (µM)				
	HOS	MDA	MCF-7	Caco-2	HCT-116
80a	42.5	24.1	15.6	34.8	16.8
80b	92	69.7	88	77.7	81
82a	18.4	15.4	9.8	23.2	11.1
82b	45.5	36.9	17.1	24.9	15
82c	25.9	18.5	2.8	20.6	7.6
82d	33.1	7.4	14.3	30.1	18.9
83a	7.3	15.5	14.3	35.7	16.6
83b	41.9	37.1	60.1	28.4	23
83c	20.4	27.7	37.8	1.7	22.7
83d	5.3	20.6	66.3	0.7	27.2
84	62.7	34.5	36.8	46.1	89.3
85a	91.2	88.3	35.6	54.3	12.8
85b	50.8	7.7	38.5	34.3	38.2
88a	58.3	26.4	85.4	59.2	64.9
88b	90.6	97.1	91.9	57.5	97.3
88c	0	28.6	35.2	13.6	30.4
88a	97.6	97.7	88.2	86.5	99.1
89b	90.4	94.2	81.9	82.7	97.1
89c	66.9	31.4	91.8	2.6	83.9

## 21. Synthesis of 1,2,3-triazole-initiated glycosides

**Scheme 38:**
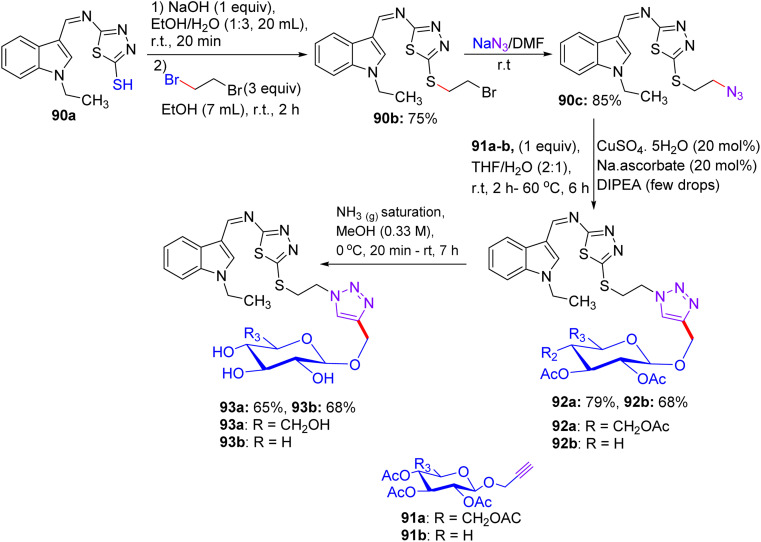
Synthetic route to indolyl-thiadiazolyl-1,2,3-triazolyl hybrid glycosides.

A new approach was proposed by Alminderej and El-Bayaa in October 2022, which initiated with the alkylation of 1,3,4-thiadiazolylindole derivative 90a to afford derivative 90b possessing an *S*-substituted bromoethyl side chain with 1,2-dibromoethane (Scheme 38). Then azidation in the presence of sodium azide led to derivative 90c with azide functionality.^58^ The azide 90c and propargyl glycosides of glucopyranosyl and xylopyranosyl moieties (91a and b) were reacted by virtue of copper-catalyzed cycloaddition (CuCAAC) reaction to produce 1,2,3-triazole glycosides 92a and 92b, by exploiting sodium ascorbate and copper sulfate as the catalyst in THF–H_2_O. The deprotection of acetylated glycosides 92a and 92b with saturated ammonia solution in methanol resulted in the formation of triazole glycosides 93a and 93b with free hydroxyl groups.

**Scheme 39:**
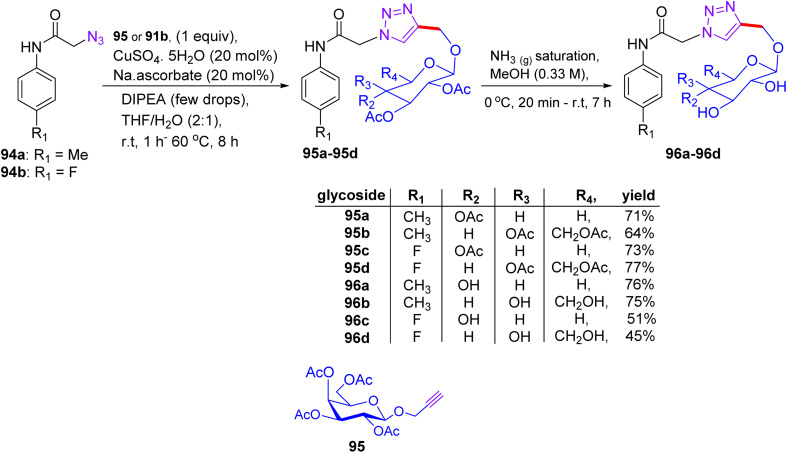
Synthetic route to arylaminoacetamide triazolyl glycosides.

Moreover, the click synthetic methodology assisted in integrating a substituted arylacetamide system to functionalize azide compounds 94a and b, which on reaction with acetylenic sugars afforded 1,2,3-triazole glycosides 95a–95d. In the end, deacetylation of triazole-glycosides 95a–95d upon hydrolysis supplied deacetylated derivatives 96a–96d in methanolic ammonia (Scheme 39). Spectral and analytical studies substantiated the synthesized compounds by confirming their purity.

### 21.1. Findings through MTT assay and binding characteristics of compounds

1,2,3-Triazole-initiated glycosides (90a–90c, 92a–93b, and 95a–96d) were assessed for their potency in HCT-116 and MCF-7 human cancer cells *via* the MTT assay; compounds 95b, 93b, and 96a showed superior potency against HCT-116 cells (IC_50_ ∼ 1–2 µM) and 93a, 96a, 95a, 90c, 93b, and 95b were effective against MCF-7 cells (IC_50_ ∼ 1–3 µM), whereas the others exhibited modest to low activity (IC_50_ ∼ 4–10 µM) compared to doxorubicin (IC_50_ ∼ 2–5 µM) (Table 23).^58^ Docking studies revealed that 95b, 93b, and 96a bind EGFR or PARP-1 (*K*_d_ ∼ 1–3 µM) with acetylated β-d-glucose to form H-bonds (for example, Lys721 and His862), and triazole-aglycone (*e.g.* aryl in 102a), enabling π–π stacking with Phe699 or Tyr907. Compounds 93a, 95a, and 90c exhibited similar binding affinities (*K*_d_ ∼ 2–4 µM), with aryl substituents enhancing their potency against MCF-7 cells. SAR analyses highlighted the lipophilicity of acetylated glycosides and rigidity of 1,2,3-triazole, with aryl groups (*e.g.* in 93b, 95b, and 96a) boosting π–π stacking for HCT-116 activity, while 90c with balanced glycosyl-aglycone showed optimised MCF-7 activity. Micro-FTIR confirmed the presence of glycosidic (C–O ∼ 1000–1100 cm^−1^), acetyl (C=O ∼ 1700 cm^−1^), and triazole (C=N ∼ 1500 cm^−1^) bonds, UFLC-DAD verified their purity, and molecular imaging showed their cytosolic/nuclear localisation. MoA involves EGFR inhibition, reduced proliferation, or PARP-1 inhibition, impairing DNA repair and leading to apoptosis, with HCT-116 sensitivity driven by higher target expression. These binding characteristics position 95b, 93b, 96a, 93a, 95a, and 90c as promising for further optimisation and study.

**Table 23:** IC_50_ values against HCT-116 and MCF-7 cell lines

Compound no.	IC_50_ (µM)	
	HCT-116	MCF-7
90a	46.9	18.1
90b	52.7	14.1
90c	30.8	1.1
92a	22.6	13.9
92b	20.8	10.8
93a	15.5	0.5
93b	4.6	4.2
94a	42.3	24.1
94b	33.3	26.4
95a	15.4	0.8
95b	2.2	5.7
95c	38.2	34.4
95d	33.8	35.3
96a	11.4	0.6
96b	35.7	20.3
96c	26.5	30.1
96d	28.8	31.6
Doxorubicin	12.1	9.4

## 22. Synthesis of quinazoline, 1,2,3-triazole, and glycopyranosyls

**Scheme 40:**
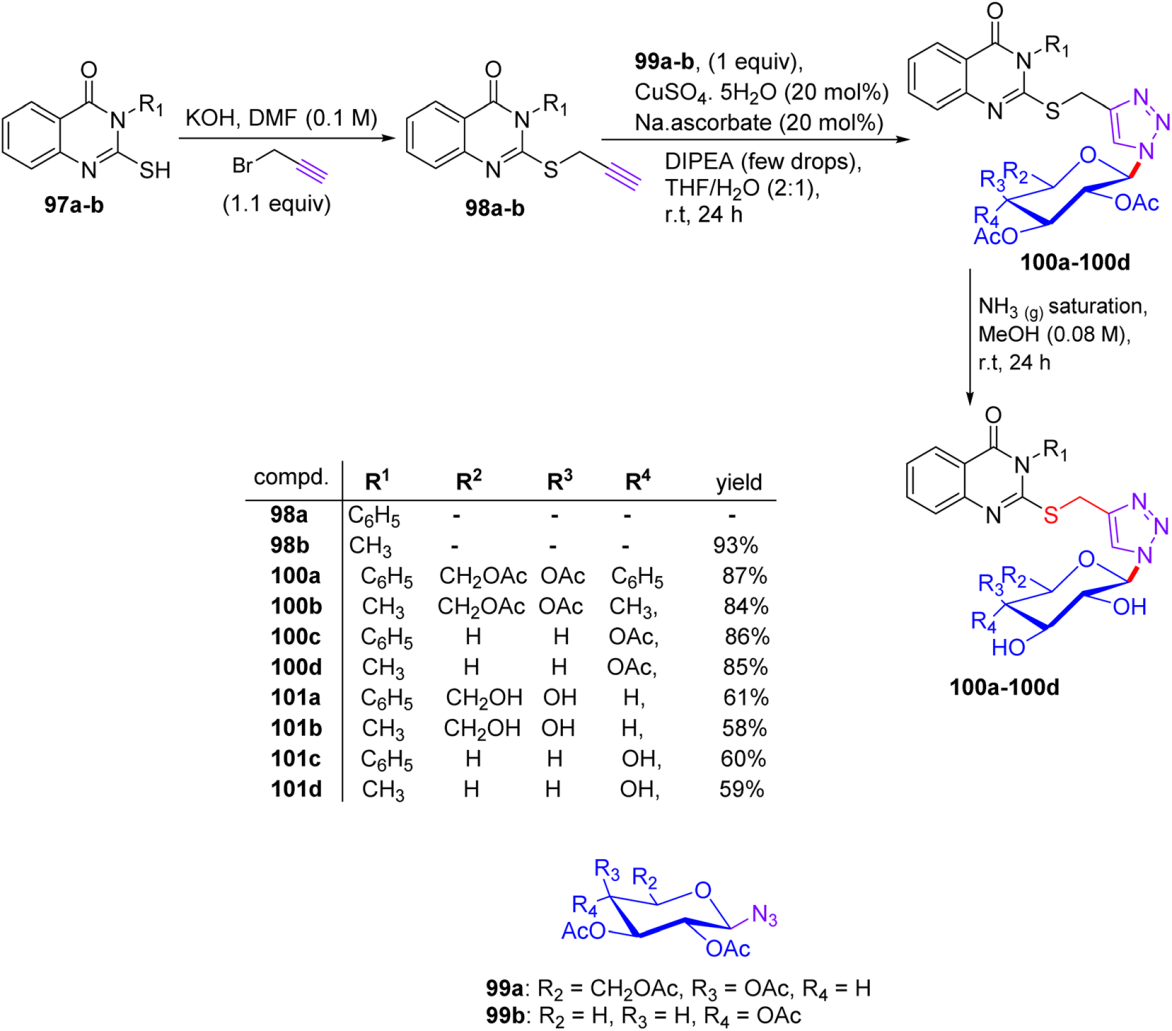
Synthesis of quinazolin-4-ones, 1,2,3-triazole, and glycopyranosyl hybrids.

Abdel-Rahman, in 2023, utilized a hybridization tactic for merging quinazolinone, 1,2,3-triazole, and glycopyranosyl systems (Scheme 40).^59^ 1,3-Dipolar cycloaddition was harnessed to give rise to the 1,2,3-triazole moiety *via* a click reaction, which was accomplished with terminal acetylenes 98a and b and glycosyl azides 99a and b substrates. The key acetylenic core was prepared from propargyl bromide and quinazolinone-thiols 97a and 97b in an alkaline environment. The glycopyranosyl azides were reacted with S-alkyne compounds 98a and 98c in the presence of Cu(i) in tetrahydrofuran/water as a solvent, yielding the anticipated 1,2,3-triazole-*N*-glycoside structure. Finally, the deacetylation of 1,2,3-triazole glycosides at room temperature in the presence of methanolic ammonia gave 1,2,3-triazole-*N*-glycosides with free hydroxyl sugar units.

### 22.1. Findings through MTT assay and binding characteristics of compounds

Quinazoline, 1,2,3-triazole, and glycopyranosyl derivatives (97a–100b and 100a–101d) were assessed for their cytotoxicity in the MCF-7, HCT-116, and HepG-2 cell lines *via* the MTT assay, targeting the EGFR and VEGFR pathways.^59^ Compounds 101a–101d showed superior activity in MCF-7 and HCT-116 cells (IC_50_ ∼ 1–2 µM) and weaker activity in HepG-2 (∼3–5 µM), whereas the others exhibited modest to low potency (IC_50_ ∼ 3–10 µM) compared to doxorubicin (IC_50_ ∼ 2–5 µM) (Table 24). Docking studies revealed that 101a–101d bind EGFR (PDB: 1M17) or VEGFR-2 (PDB: 4ASD, *K*_d_ ∼0.5–2 µM), with acetylated β-d-glucose forming H-bonds (*e.g.*, Lys721 and Asp1046) and the quinazoline-triazole core enabling π–π stacking with Phe699 or Phe1047. Compounds 97a–100b and 100a–100d showed weaker binding (*K*_d_ ∼ 2–5 µM) owing to non-acetylated glycosides or alkyl substituents. SAR analyses highlighted the lipophilicity of acetylated glycopyranosyls and rigidity of 1,2,3-triazole, with aryl substituents (*e.g.* in 101c and 101d) enhancing π–π stacking for MCF-7/HCT-116 potency, while the ATP-mimetic core of quinazoline optimises kinase inhibition. Micro-FTIR confirmed the presence of glycosidic (C–O ∼ 1000–1100 cm^−1^), acetyl (C=O ∼ 1700 cm^−1^), and quinazoline/triazole (C=N ∼ 1500 cm^−1^) bonds, UFLC-DAD verified their purity, and molecular imaging showed their cytosolic/membrane localization. MoA involves EGFR/VEGFR-2 inhibition, reducing proliferation and angiogenesis, leading to apoptosis *via* caspase activation. 101a–101d exhibited high activity in MCF-7/HCT-116 cells due to receptor overexpression. These binding characteristics position 101a–101d as promising leads complementing cardiac glycosides.^39^

**Table 24:** IC_50_ results for quinazolinone-1,2,3-triazole glycosides 117a–118d against EGFR and VEGFR

Compound no.	IC_50_ (µM)	
	EGFR	VEGFR
100a	18.63	32.25
100b	17.94	36.33
100c	21.38	28.45
100d	23.05	30.6
101a	16.75	22.33
101b	0.35	22.86
101c	15.88	13.63
101d	0.31	3.2
Erlotinib	0.22	—
Sorafenib	—	1.88

## 23. Synthesis of triazole-linked *N*-glycosides of pyrazolo[1,5-*a*]pyrimidinones

**Scheme 41:**
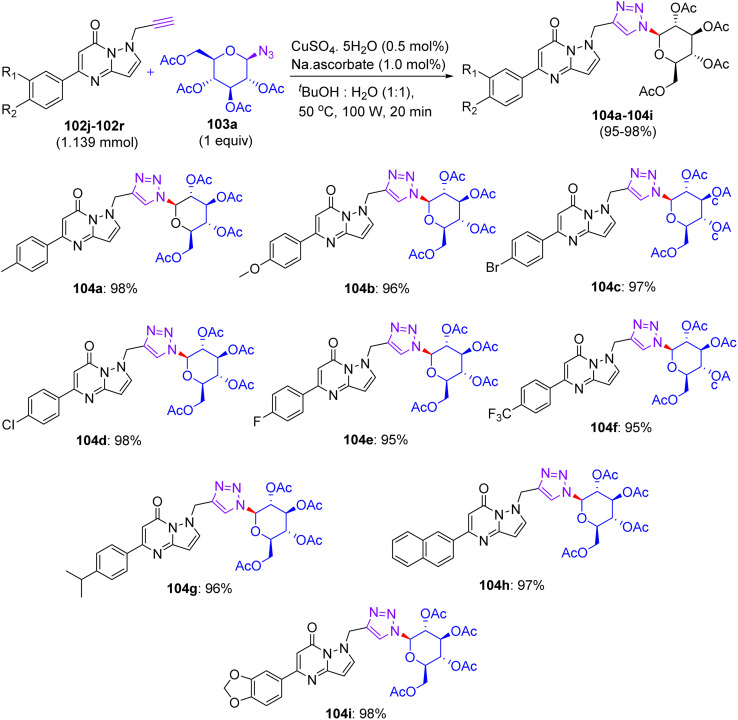
Synthesis of triazole-bridged *N*-glucoside compounds 104a–104i.

This synthesis procedure was reported by Sagar *et al.* in 2024, where pyrazolopyrimidin-7-ol derivatives were converted into a series of *N*-propargylated pyrazolo[1,5-*a*]pyrimidinones 102a–102i by applying propargyl bromide and K_2_CO_3_ in 1,4-dioxane.^60^ In succession, they treated compounds 102a–102i with 1-azidoglucoside 103a to afford triazole-bridged *N*-glucosides 104a–104i by CAAC under microwave irradiation at 50 °C for 20 min, in excellent yields (Scheme 41).

**Scheme 42:**
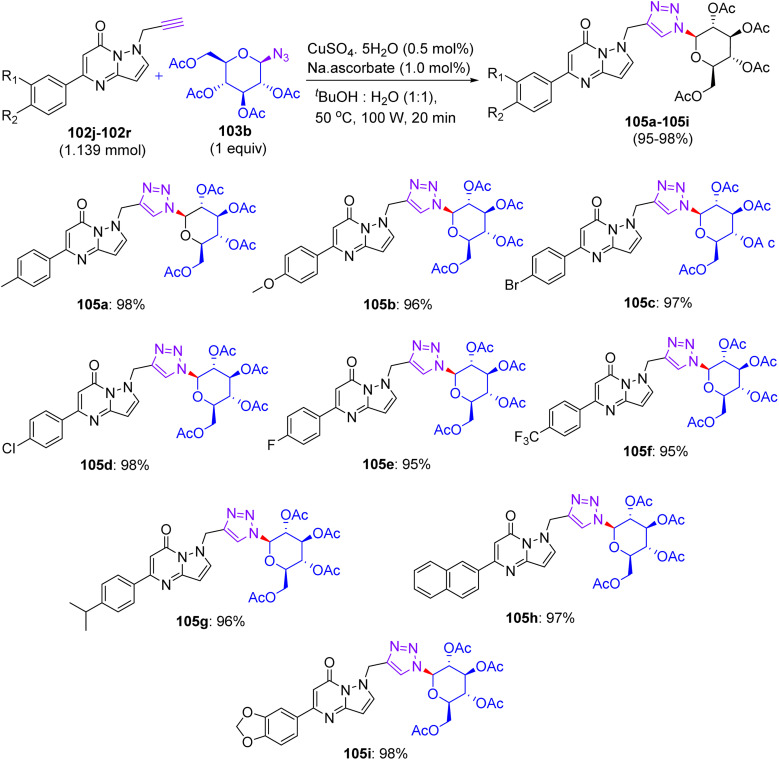
Synthesis of triazole-bridged *N*-galactoside compounds 105a–105i.

**Scheme 43:**
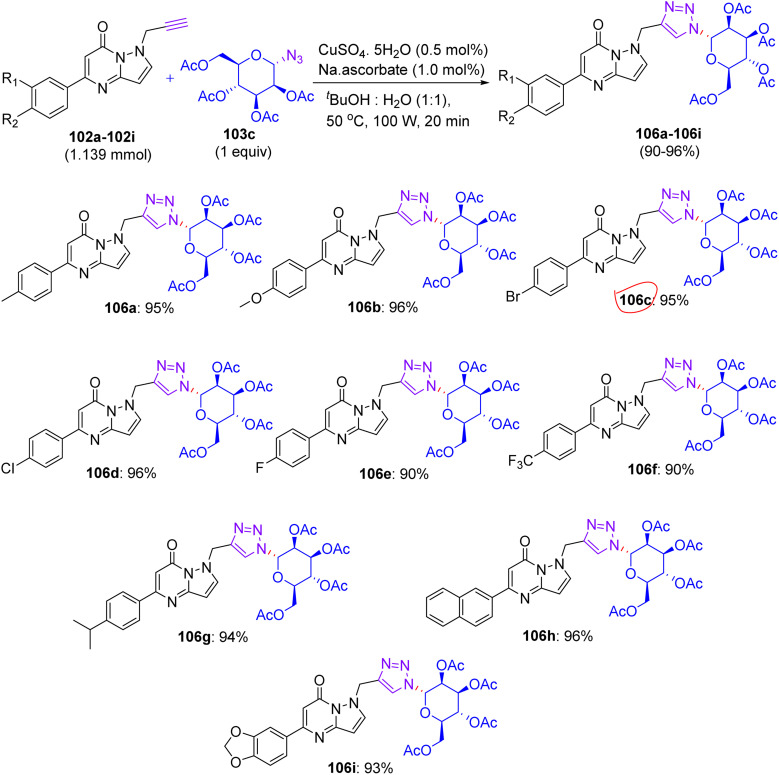
Synthesis of triazole-bridged *N*-mannosides compounds 106a–106i.

In the same way, *N*-propargylated pyrazolo[1,5-*a*]pyrimidinones 102a–102i were reacted with 1-azidogalactoside 103b to achieve triazole-bridged *N*-galactosides 105a–105i (Scheme 42). Furthermore 102a–102i reacted with 1-azidomannoside 103c under analogous microwave irradiation conditions, establishing triazole-bridged *N*-mannosides 106a and 106b (Scheme 43).

### 23.1. Findings through MTT assay and binding characteristics of compounds

Triazole-bridged *N*-glycosides of pyrazolo[1,5-*a*]pyrimidinones (104a–104i, 105c, 105f, and 106b) were explored for growth inhibition *via* the MTT assay on MDA-MB-231 and MCF-7 breast cancer cells. Compounds 104b and 105f showed superior activity in MDA-MB-231 (IC_50_ ∼ 1–2 µM) and MCF-7 (IC_50_ ∼ 1–2 µM), respectively, and 104d, 104i, 105c, and 106b exhibited notable potency (IC_50_ ∼ 2–4 µM) (Tables 25 and 26).^60^ Docking studies reveal that 104b and 105f bind EGFR (PDB: 1M17) or PI3K (PDB: 3L08, *K*_d_ ∼ 0.5–2 µM), with acetylated β-d-glucose (104b) or ribose (105f) forming H-bonds (*e.g.*, Lys721 and Asp933) and the pyrazolo[1,5-*a*]pyrimidinone-triazole core enabling π–π stacking with Phe699 or Trp812. Compounds 104d, 104i, 105c, and 106b show slightly weaker binding (*K*_d_ ∼ 2–4 µM) due to glycosyl or substituent variations. SAR analyses highlighted the lipophilicity (104b) and ribose transporter affinity (105f), with aryl substituents enhancing π–π stacking for cell-specific potency (MDA-MB-231 for 104b and MCF-7 for 105f). Micro-FTIR confirmed the presence of *N*-glycosidic (C–O ∼ 1000–1100 cm^−1^), acetyl (C=O ∼ 1700 cm^−1^), and triazole/pyrimidinone (C=N ∼ 1500 cm^−1^) bonds, UFLC-DAD verified their purity, and molecular imaging showed their cytosolic/membrane localisation. MoA involves EGFR/PI3K inhibition, reducing proliferation and survival, and leading to apoptosis *via* caspase activation, with cell-specificity driven by receptor overexpression. These binding characteristics position 104b, 105f, 104d, 104i, 105c, and 106b as promising leads.

**Table 25:** Anticancer activity results through cell viability assessment

Compound no.	Cell viability (%)		
	50 µM	25 µM	10 µM
104a	85.37	87.49	92.4
104b	34.32	49.03	65.14
104d	98.54	100.13	100.25
104e	86.22	93.24	98.58
104f	54.56	59.98	76.61
104g	74.75	89.8	93.24
104h	69.46	99.07	102.13
104i	88.43	81.48	89.1
104j	42.82	55.17	86.58
105a	78.81	88.47	81.68
105b	72.48	86.2	92.51
105c	46.98	63.28	66.7
105d	73.04	76.76	78.46
105e	75.33	78.62	82.4
Menadione (20 µM)	23.76	—	—

**Table 26:** Anticancer activity results through cell viability assessment

Compound no.	Cell viability (%)		
	50 µM	25 µM	10 µM
105f	41.2	46.1	70.37
105g	78.74	80.51	85.23
105h	67.7	89.59	92.04
105i	97.5	98.25	101.28
106a	91.74	92.81	99.05
106b	44.18	54.28	66.7
106c	75.55	78.52	81.32
106d	71.36	95.16	99.5
106e	89.57	90.13	98.41
106f	86.98	91.06	96.84
106g	83.54	75.49	80.63
106h	81.53	89.82	101.77
106i	92.89	93.31	96.45
YM155 (20 µM)	23.39	—	—

## 24. Conclusions

The development of novel anticancer agents requires approximately $2.7 billion over 12 years to reach the market. The integration of artificial intelligence (AI) and machine learning (ML) has revolutionised this process by enabling retrosynthetic analysis, molecular skeleton design, and binding affinity calculations, thereby significantly reducing time and cost.^61,62^ ML algorithms accelerate lead identification within approximately one month compared to traditional phenotypic probes through computational analyses linking IC_50_ curves to 2D and 3D cellular dissections.^63,64^ Optimised synthetic strategies, including glycoconjugation, have been developed to enhance aqueous solubility,^65^ CuAAC-mediated synthesis of triazole-linked glycoconjugates^66^ and radical coupling of *C*-glycosylated products,^67^ and the photoredox-sensitive “tag and edit” methods for selective OH group modification have facilitated the development of potent glycosidic derivatives targeting epigenetics (DNA/histone modifiers),^68^ Hedgehog pathway inhibitors,^69^ and CDK11 (ref. 70) and MEK inhibitors,^71^ and other regulators. However, despite these advances, challenges persist in selecting the optimal targets and designing specific treatments. Computational biology, leveraging docking and MD, has proven instrumental in addressing specificity and selectivity and optimising the binding of glycosidic compounds to diverse targets. These carbohydrate-based conjugates rank among promising anticancer leads, provided that their synthesis is supported by thorough structure elucidation, multi-omics, and robust laboratory methodologies.^72^

## Author contributions

Dr S. Ali conceived the idea; Gul Rukh wrote the manuscript; Dr S. Ali and Dr S. Adnan Ali Shah supervised and revised the manuscript; and Dr R. Ajaj, Dr A. Rauf, and Dr H. A. Ogaly provided the relevant literature and reviewed/revised the biology part of the manuscript.

## Conflicts of interest

There are no conflicts to declare.

## Data Availability

The authors declare that no new data were generated in this review article.
